# Polyfunctionalized *N*-Arylsulfonyl Indoles: Identification of (*E*)-*N*-Hydroxy-3-{3-[(5-(3-(piperidin-1-yl)propoxy]-1*H*-indol-1-yl)sulfonyl]phenyl}acrylamide (MTP150) for the Epigenetic-Based Therapy of Parkinson’s Disease

**DOI:** 10.3390/ijms27073135

**Published:** 2026-03-30

**Authors:** Mireia Toledano-Pinedo, Alicia Porro-Pérez, Linda Schäker-Hübner, Daniel Diez-Iriepa, Isabel Iriepa, Agata Siwek, Małgorzata Wolak, Grzegorz Satała, Andrzej J. Bojarski, Agata Doroz-Płonka, Jadwiga Handzlik, Justyna Godyń, Patrick Dallemagne, Christophe Rochais, Audrey Davis, Marc Since, Belén Pérez, Aina Bellver-Sanchis, Alba Irisarri, Mercè Pallàs, Cristina Solana-Manrique, Francisco López-Muñoz, Lhassane Ismaili, Christian Griñán-Ferré, Nuria Paricio, Finn K. Hansen, Anna Więckowska, José Marco-Contelles

**Affiliations:** 1 Institute of General Organic Chemistry (CSIC), C/Juan de la Cierva 3, 28006 Madrid, Spain; mireia.toledano@iqog.csic.es (M.T.-P.); aliporro@ucm.es (A.P.-P.); daniel.diezi@uah.es (D.D.-I.); 2 Pharmaceutical Institute, University of Bonn, An der Immenburg 4, 53121 Bonn, Germany; lschaeke@uni-bonn.de (L.S.-H.); finn.hansen@uni-bonn.de (F.K.H.); 3 Departamento de Química Orgánica y Química Inorgánica, Instituto de Investigación Química “Andrés M. del Río” (IQAR), Universidad de Alcalá, 28805 Alcalá de Henares, Spain; isabel.iriepa@uah.es; 4 Grupo DISCOBAC, Instituto de Investigación Sanitaria de Castilla-La Mancha (IDISCAM), 45071 Toledo, Spain; 5 Department of Pharmacobiology, Faculty of Pharmacy, Medical College, Jagiellonian University, 9 Medyczna St., 30-688 Krakow, Poland; agata.siwek@uj.edu.pl (A.S.); malgorzata.dybala@uj.edu.pl (M.W.); 6 Maj Institute of Pharmacology, Polish Academy of Sciences, 12 Smętna Street, 31-343 Krakow, Poland; satala@if-pan.krakow.pl (G.S.); bojarski@if-pan.krakow.pl (A.J.B.); 7 Department of Technology and Biotechnology of Drugs, Medical College, Jagiellonian University, 9 Medyczna Street, 30-688 Krakow, Poland; a.doroz-plonka@uj.edu.pl (A.D.-P.); j.handzlik@uj.edu.pl (J.H.); 8 Department of Physicochemical Drug Analysis, Faculty of Pharmacy, Medical College, Jagiellonian University, 9 Medyczna Street, 30-688 Krakow, Poland; justyna.godyn@uj.edu.pl (J.G.); anna.wieckowska@uj.edu.pl (A.W.); 9 Université de Caen Normandie, Normandie University, CERMN UR4258, F-14000 Caen, France; patrick.dallemagne@unicaen.fr (P.D.); christophe.rochais@unicaen.fr (C.R.); 10 Université de Caen Normandie, Normandie University, CERMN UR4258, Druid Platform, F-14000 Caen, France; audrey.davis@unicaen.fr (A.D.); marc.since@unicaen.fr (M.S.); 11 Department of Pharmacology, Therapeutic and Toxicology, Universitat Autònoma de Barcelona, 08193 Barcelona, Spain; belen.perez@uab.cat; 12 Pharmacology Section, Department of Pharmacology, Toxicology and Therapeutic Chemistry, Faculty of Pharmacy and Food Sciences, Institute of Neuroscience, Universitat de Barcelona (NeuroUB), Av. Joan XXIII 27–31, 08028 Barcelona, Spain; abellversanchis@gmail.com (A.B.-S.); albairisarrim@gmail.com (A.I.); pallas@ub.edu (M.P.); christian.grinan@ub.edu (C.G.-F.); 13 Institut de Neurociències, Universitat de Barcelona (NeuroUB), 08193 Barcelona, Spain; 14 Spanish Biomedical Research Center in Neurodegenerative Diseases (CIBERNED), Instituto de Salud Carlos III, 28029 Madrid, Spain; 15 Departamento de Genética, Facultad CC Biológicas, Universidad de Valencia, 46100 Burjassot, Spain; cristina.solana@uv.es; 16 Instituto Universitario de Biotecnología y Biomedicina (BIOTECMED), Universidad de Valencia, 46100 Burjassot, Spain; 17 Faculty of Health Sciences–HM Hospitals, Camilo José Cela University, 28692 Madrid, Spain; flopez@ucjc.edu; 18 HM Hospitals Health Research Institute, 28015 Madrid, Spain; 19 Neuropsychopharmacology Unit, “Hospital 12 de Octubre” Research Institute, 28041 Madrid, Spain; 20 Laboratoire de Recherches Intégratives en Neurosciences et Psychologie Cognitive de Besançon, Groupe Chimie Médicinale, Université de Franche-Comté, INSERM, UMR 1322 LINC, F-25000 Besançon, France; lhassane.ismaili@univ-fcomte.fr

**Keywords:** Biological evaluation, ChE enzymes, *Drosophila* in vivo models, 5-HT_6_ receptor, MTP150, neurodegenerative diseases, Parkinson’s disease, synthesis

## Abstract

Herein, we have identified the polyfunctionalized 1-(phenylsulfonyl)-1*H*-indole-2-carboxylic acid derivative MTP150 for the treatment of neurodegenerative diseases owing to its efficacy in reducing protein aggregation, modulating matrix metalloproteinase activity, mitigating neuroinflammation, and enhancing DNA damage repair pathways across in vivo *Caenorhabditis elegans* models of Alzheimer’s disease, Parkinson’s disease (PD), and Huntington’s disease. Further experiments in an in vivo *Drosophila* model of PD showed that MTP150 increased motor performance, reduced oxidative stress levels, and restored mitochondrial function in model flies. In addition, MTP150 exhibited neuroprotective effects in PD model cells, thereby supporting its therapeutic potential for this disease.

## 1. Introduction

**Figure 1:**
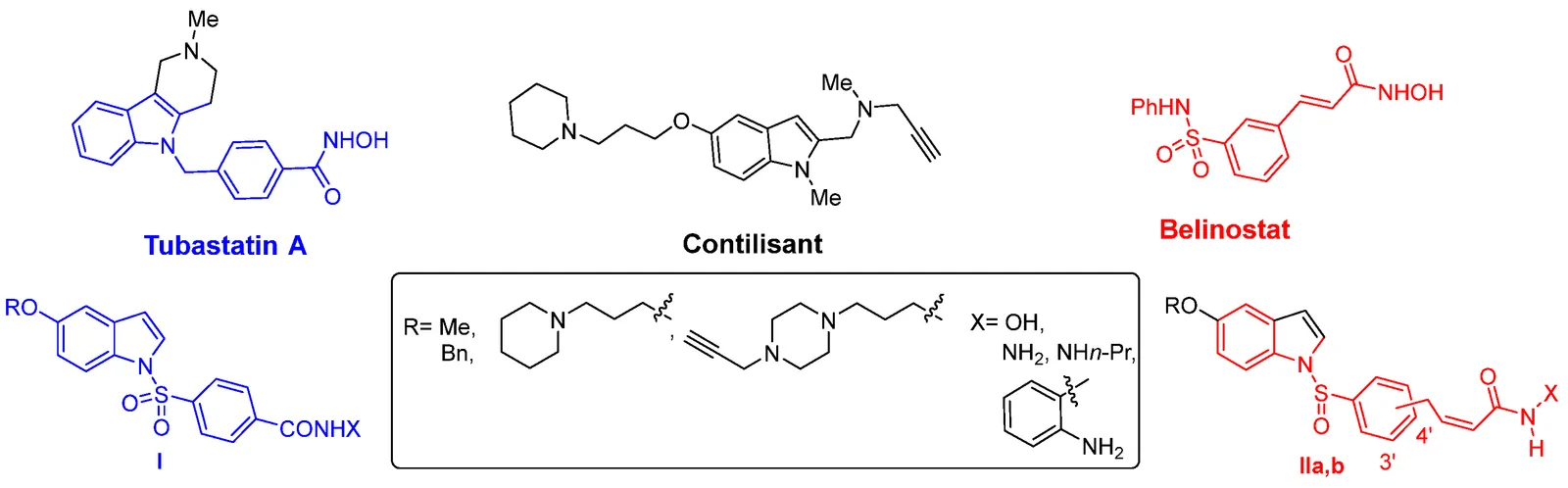
Structures of Contilisant, Tubastatin A, and Belinostat, and the general structure of the polyfunctionalized *N*-arylsulfonyl indoles **I** and **IIa**,**b**.

In projects targeting the identification of new multitarget small molecules (MSMs) for the treatment of neurodegenerative diseases [1,2], we have recently described Contilisant+Tubastain A (**I**) [3] and Contilisant+Belinostat (**II**) [4] hybrids resulting from the juxtaposition of selected pharmacophoric groups present in Tubastatin A [5] and Belinostat [6], respectively (Figure 1), reporting also their biological evaluation on cholinesterase (ChE), monoamine oxidase (MAO), and histone deacetylase (HDAC) enzymes.

Contilisant [7] (Figure 1) was the first described permeable, antioxidant, neuroprotective multitarget MSM showing high affinity for the human histamine 3 receptor (*h*H_3_R) (K*_i_* = 10.8 nM) and human sigma 1 receptor (*h*σ_1_R) (K*_i_* = 65 nM), as well as significant inhibition of *h*MAO B (IC_50_ = 78 nM) and *h*AChE (IC_50_ = 530 nM). At a dose of 1 mg/kg i.p., Contilisant retrieved the memory deficit induced by lipopolysaccharide in a recognition test in mice and significantly restored the cognitive deficit caused by A*β*_1–42_ in the radial maze assay in an in vivo Alzheimer’s disease (AD) test, comparing very favorably with donepezil [7].

On the other hand, Tubastatin A (**4**) (Figure 1) is a highly selective class IIb HDAC inhibitor (HDACi) that is able to suppress the degeneration of cultivated neurons from the cerebral cortex under oxidative stress conditions [5]. As overexpressed HDACs have been associated with diverse types of cancer, the efficiency of HDACis, in particular nonselective pan-HDACis, as anticancer drugs has been primarily documented. This is the case of Belinostat (Figure 1), one of four FDA-approved HDACis for the treatment of relapsed/refractory peripheral T-cell lymphoma [6].

**Figure 2:**
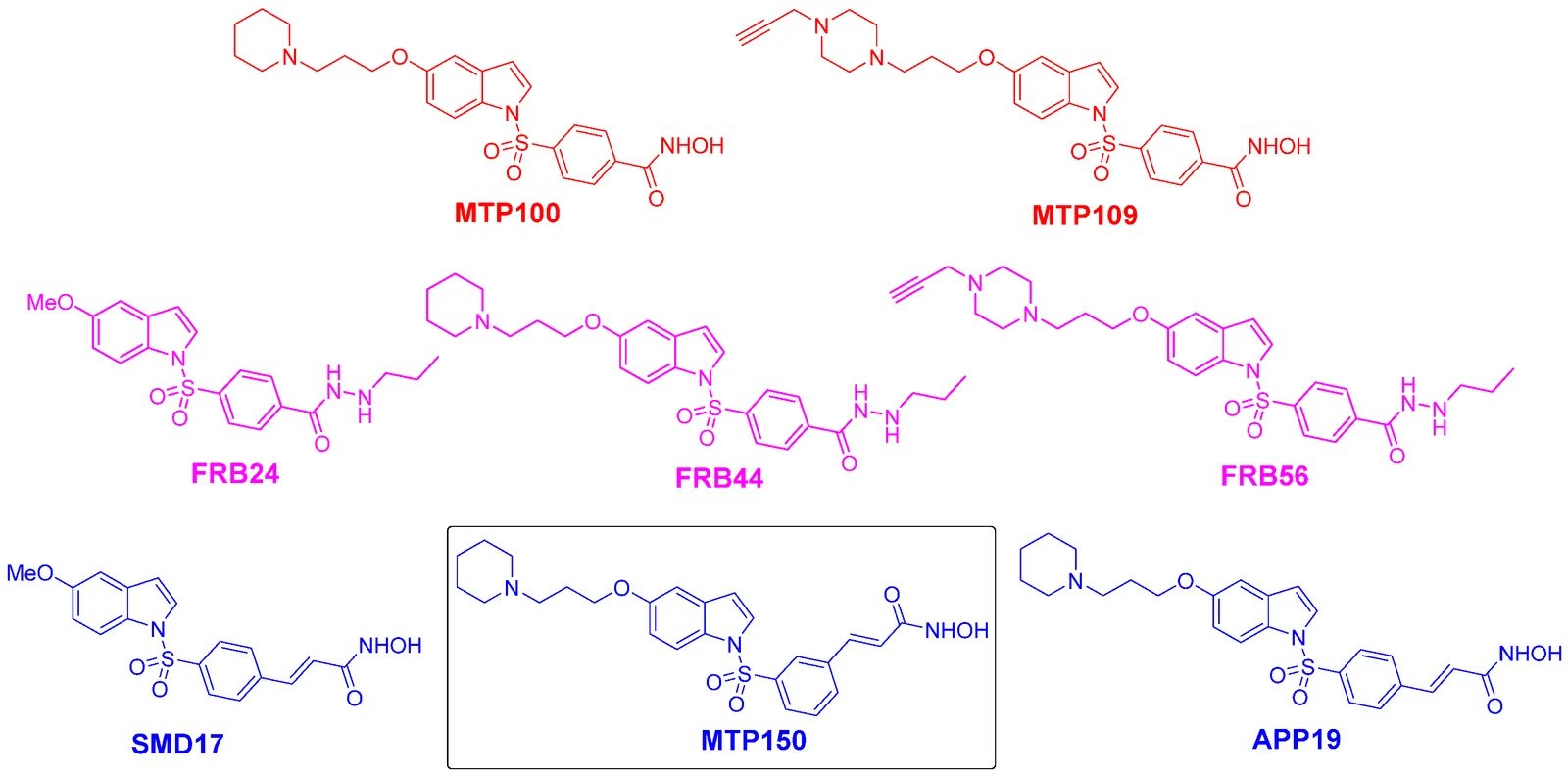
Structures of selected Contilisant+Tubastatin A hybrids and Contilisant+Belinostat hybrids.

Among Contilisant+Tubastatin A hybrids of type **I** (Figure 1), compounds MTP100/MTP109 (Figure 2) were identified as potent HDAC6 inhibitors (HDAC6is), showing IC_50_ values of 0.012 μM and 0.035 μM, respectively. Consequently, they were further evaluated in *Drosophila* and human cell models of Parkinson’s disease (PD), with both compounds attenuating PD-like phenotypes, including motor defects, oxidative stress, and mitochondrial dysfunction, in PD model flies [3]. Compounds MTP100 and MTP109 (Figure 2) were also studied in the transgenic *Caenorhabditis elegans* (*C. elegans*) CL2006 model of AD. Both compounds did not induce undesirable animal functional changes but inhibited age-related paralysis and improved cognition in the thrashing assay. Furthermore, Contilisant+Tubastatin A hybrids FRB24, FRB44, and FRB56 (Figure 2), being potent and selective HDAC1 inhibitors (HDAC1is), are promising agents for the treatment of glioblastoma [8].

Finally, among the Contilisant+Belinostat hybrids of type **II** (Figure 1), compounds SMD17 (HDAC1 IC_50_ = 0.086 μM; HDAC6 IC_50_ = 0.017 μM) (Figure 2), MTP150 (Figure 2) (HDAC1 IC_50_ = 0.019 μM; HDAC6 IC_50_ = 0.040 μM; AChE IC_50_ = 20.06 μM; BuChE IC_50_ = 17.10 μM; MAO-B IC_50_ = 2.14 μM), and APP19 (Figure 2) (HDAC1 IC_50_ = 0.126 μM; HDAC6 IC_50_ = 0.020 μM; AChE IC_50_ = 2.73 μM; BuChE IC_50_ = 4.03 μM; MAO-B IC_50_ = 1.18 μM) emerged as the most promising candidates as potential treatments for AD owing to their unique inhibition profiles and favorable mode of inhibition [4].

In the search for efficient MSMs for the treatment of AD [9,10], a growing body of evidence highlights the promising therapeutic potential of 5-HT_6_R antagonists, which enhance memory and learning processes [11]. 5-HT_6_R is expressed in different brain areas that are responsible for cognitive function [12]. Results of phase II clinical trials showed that a combination therapy using idalopirdine, a 5-HT_6_R antagonist, and donepezil, an AChE inhibitor (AChEi), resulted in a superior therapeutic effect in AD patients compared to monotherapy [13].

Previous studies have shown that the 5-HT_6_ receptor (5-HT_6_R) is associated not only with cognitive impairment but also with the behavioral and psychological symptoms of AD, and it therefore represents an interesting biological target in the search for AD therapies [14]. The pro-cognitive activity of 5-HT_6_R antagonists has been demonstrated in animal models of cognitive dysfunction, learning, and memory [15], as well as in phase II clinical trials in patients with moderate AD [16]. Combination therapy using idalopirdine or intepirdine (5-HT_6_R antagonists) [17] together with donepezil showed enhanced effects on cognitive function compared with therapy using donepezil alone [18,19]. Although neither idalopirdine nor intepirdine demonstrated efficacy in phase III clinical trials, the search for new, effective 5-HT_6_R antagonists for the treatment of AD continues. Latrepirdine, SAM-760, and masupirdine (SUVN-502) have also reached phase II clinical trials, with masupirdine currently in phase III clinical trials evaluating its efficacy in the treatment of agitation (psychomotor agitation) in AD-type dementia [20,21,22].

A number of 5-HT_6_ antagonists have been developed, including compounds incorporating the *N*(1)-benzenesulfonamide motif into an indole template [23]. In this context, compounds of type I and II have been functionalized with the *N*(1)sulfonamideindole motif to specifically focus on their pharmacological profile on 5-HT_6_R, thereby improving and enhancing their potential capacity to act as therapeutic agents for NDs such as AD or PD. The discovery of non-basic 5-HT_6_R ligands [24,25] with high affinity and additional advantages—namely, no risk of hERG channel blockade and increased selectivity—has spurred further research in this direction. In our project, we also obtained and investigated such a group of compounds.

Until now, we have seldom addressed the ability of these compounds to modulate this receptor, which is most likely one of the key factors explaining the in vivo reported results for these compounds [3].

**Figure 3:**
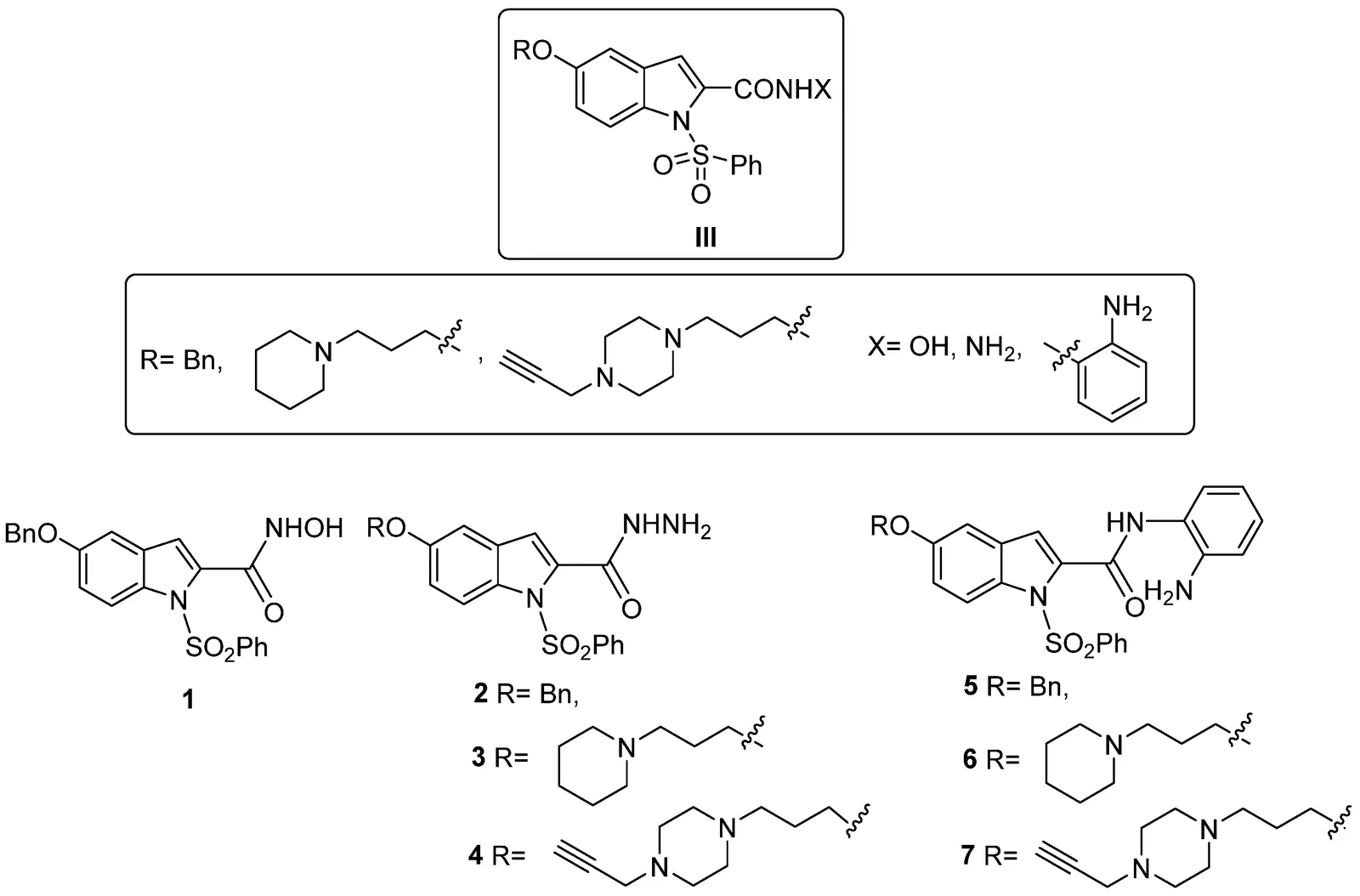
Structures of the Contilisant analogs of type **III** and hybrids **1**–**7**.

Thus, prompted by these results, in this study, we describe the capacity of selected hybrids of types **I** and **II** (Figure 1) to modulate 5-HT_6_R. Based on the obtained results of a new structure–activity relationship (SAR) project, we designed a novel group of Contilisant analogs of type **III** (Figure 3), the synthesis and biological evaluation (ChEs, MAOs, HDAC, and 5-HT_6_R) of which are also reported here. Very interestingly, we finally identified a non-toxic and permeable ligand MTP150 (Figure 2) as a very efficient MSM in selected biological targets (ChEs, MAOs, HDAC1,6, and 5-HT_6_R). Furthermore, compound MTP150 was shown to promote neuroprotective capacity across distinct neurodegenerative mechanisms in in vivo *C. elegans.* Furthermore, in the PD *Drosophila* in vivo model, MTP150 proved to be efficient in increasing motor performance, reducing oxidative stress levels, and restoring mitochondrial function in model flies exhibiting neuroprotective effects in PD model cells.

## 2. Results & Discussion

### 2.1. Analysis of the Affinity of Hybrids of Type I and II for 5-HT_6_R

#### 2.1.1. Contilisant+Tubastatin A Hybrids of Type **I**

The analysis of the affinity of Contilisant+Tubastatin A hybrids of type **I** for 5-HT_6_R, performed as previously described [26], afforded the K_i_ values shown in Table 1. As shown, most of the compounds acted as affine 5-HT_6_R ligands in the low micromolar range, the most potent being *o*-aminoanilide MTP165 (K_i_ = 1462 nM), bearing a piperidinepropoxy motif at C5, and the least potent being *o*-aminoanilide MTP155 (K_i_ = 57960 nM), bearing a benzyloxy motif at C5. Among the ligands with two-digit micromolar power, the order of modulation from the most to least potent ligands was MTP99 (K_i_ = 16220 nM), MTP96 (K_i_ = 17360 nM), FRB21 (K_i_ = 19370 nM), FRB56 (K_i_ = 30650 nM), MTP90 (K_i_ = 31510 nM), and MTP155 (K_i_ = 57960 nM). Very interestingly, among these six ligands, there were three hydrazides (MTP99, MTP96, MTP90), two substituted hydrazides (FRB21, FRB56), and one *o*-aminoanilide (MTP155), combined with diverse substituents at C5 on the indole core. Among the ligands with one-digit micromolar power, the three most potent ligands were MTP165 (K_i_ = 1462 nM), MTP98 (K_i_ = 1543 nM), and MTP100 (K_i_ = 3392 nM), bearing a *o*-aminoanilide/piperidinepropoxy at C5, a hydroxamate/BnO at C5, and a hydroxamate/piperidinepropoxy at C5, respectively. Consequently, although no clear SAR could be drawn, some interesting trends could be proposed, indicating that hydroxamates bearing a piperidinepropoxy at C5 are preferred 5-HT_6_R modulators.

It is interesting to highlight that, regarding HDAC1/6 inhibition, among the Contilisant+Tubastatin A hybrids of type I, modest affine 5-HT_6_R ligands FRB24 (Ki = 5820 nM) and FRB44 (Ki = 4307 nM) displayed the most potent HDAC1 inhibition [FRB24 (IC_50_ = 0.087 ± 0.017 μM); FRB44 (IC_50_ = 0.112 ± 0.018 μM)] [3]. Similarly, modest affine 5-HT_6_R ligands MTP100 (Ki = 3392 nM) and MTP109 (Ki = 6376 nM) showed the most potent HDAC6 inhibition [MTP100 (IC_50_ = 0.012 ± 0.001 μM); MTP109 (IC_50_ = 0.035 ± 0.002 μM)] [3]. In some cases, the same compounds showed inhibition for *h*MAO B and *eq*BuChE or affinity for *h*H_3_R. This was the case for ligands MTP100 [*h*H_3_R (43%)], FRB44 [*eq*BuChE (IC_50_ = 1.180 ± 0.032 μM)], MTP165 [*eq*BuChE (IC_50_ = 0.263 ± 0.005 μM)], and MTP195 [*eq*BuChE (IC_50_ = 0.896 ± 0.029 μM); MAO B (IC_50_ = 0.562 ± 0.007 μM)] [3]. Note also that compounds MTP96 (K_i_ = 17360 nM), MTP99 (K_i_ = 16220 nM), and FRB56 (K_i_ = 30650 nM), being poor affine 5-HT_6_R ligands, were modest *eq*BuChEis [MTP96 (IC_50_ = 2.271 ± 0.144 μM), FRB56 (IC_50_ = 8.806 ± 0.231 μM)] or potent MAO Bis [MTP99 (IC_50_ = 0.312 ± 0.011 μM), FRB56 (IC_50_ = 0.384 ± 0.023 μM)] [3].

To sum up, based on the values in Table 1 and those previously reported for ChEs, MAOs, and H_3_R binding [3], the three most balanced MSMs were compounds FRB44, MTP195, and MTP165, with ligands FRB44 and MTP195 showing very similar in vitro pharmacological profiles. Compounds FRB44 and MTP195 were potent and selective HDAC1is and modulated 5-HT_6_R in the same range, being the third most potent among all of the tested ligands. Furthermore, both showed strong capacity to inhibit BuChE, but ligand MTP195, not FRB44, was able to inhibit MAO B (IC_50_ = 0.562 ± 0.007 μM). Finally, ligand MTP165, a potent and selective HDAC1i, was the most affine 5-HT_6_R ligand (K_i_ = 1462 nM), able to modulate H_3_R (48 ± 3%), and the most potent BuChEi (IC_50_ = 0.263 ± 0.005 μM). Note that ligands MTP195 and MTP165 are *o*-aminoanilides, bearing *N*-propargylpiperazinepropoxy and piperidinepropoxy motives at C5, respectively, whereas ligand FRB44 is a *N*-propylhydrazide, bearing a piperidinepropoxy group at C5.

#### 2.1.2. Contilisant+Belinostat Hybrids of Type **II**

The affinity of Contilisant+Belinostat hybrids of type **II** for 5-HT_6_R is summarized in Table 2. All ligands bound 5-HT_6_R with modest [compound MTP143: Ki = 2243 nM)] to poor [compound MTP142: Ki = 38610 nM)] affinity, showing Ki values higher than that determined for Belinostat. The most potent was *o*-aminoanilide MTP143, bearing a methoxy group at C5, followed by *o*-aminoanilide APP17, bearing a piperidinepropoxy group at C5. Note also that the hit compounds MTP150 and APP19, previously identified as the most balanced for their in vitro profiles on several biological targets [4], proved to be very modest 5-HT_6_R modulators, showing Ki = 13580 nM and Ki = 8670 nM, respectively. Interestingly, ligand MTP143 showed no activity against the diverse biological targets tested (ChEs, MAOs, and H_3_R) [4], whereas *o*-aminoanilide APP17 was a modest but selective HDAC1i, a moderate and modestly selective BuChEi, and devoid of MAO-inhibition activity [4]. This means that overall, after the 5-HT_6_R analysis, ligands MTP150 and APP19 remained the most balanced compounds for further selection and investigation.

### 2.2. Contilisant Analogs of Type **III**

#### 2.2.1. Synthesis

Based on the described results for hybrids of types **I** and **II**, and to improve their pharmacological profile, we next designed Contilisant analogs of type **III** (Figure 3). The compounds were designed by juxtaposing the *N*-benzenesulfonyl motif present in 5-HT_6_R antagonists [17] with selected pharmacophore groups present in Contilisant (Figure 1) and by eliminating the *N*-methylpropargylmethyl motif at C2 present in Contilisant and installing instead the HDAC pharmacophore group at a carboxylic acid motif attached at C2 of the indole core. This process resulted in compounds **1**–**7** (Figure 3) bearing key and selected Zn-binding groups, such as hydroxamic acid, hydrazide, or *o*-aminoanilide groups. This selection forced us to use, in addition to piperidine, a *piperazine* motif in the western part of the molecules to install the *N-*propargyl motif as the required and necessary MAO pharmacophoric group.

**Scheme 1:**
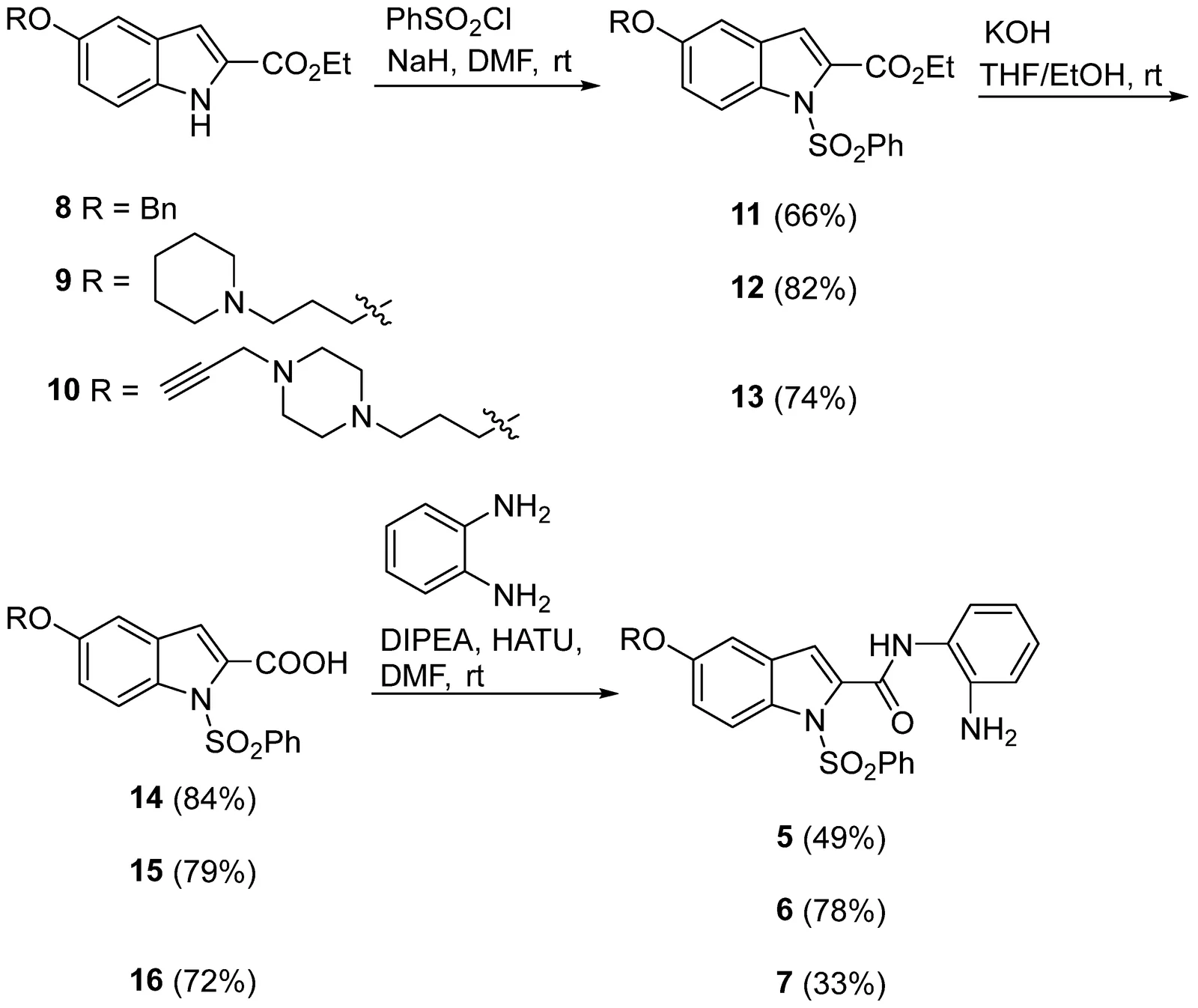
Synthesis of compounds **5**–**7**.

**Scheme 2:**
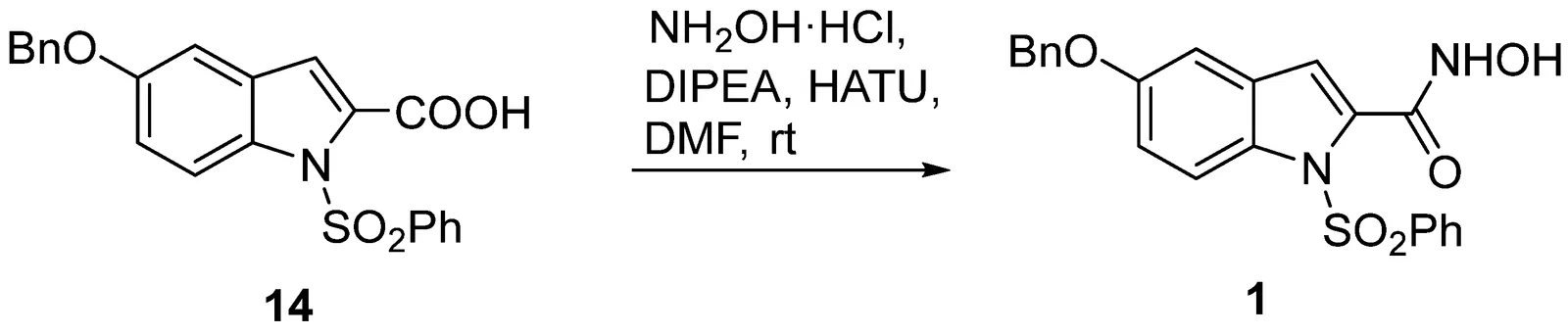
Synthesis of compound **1**.

**Scheme 3:**
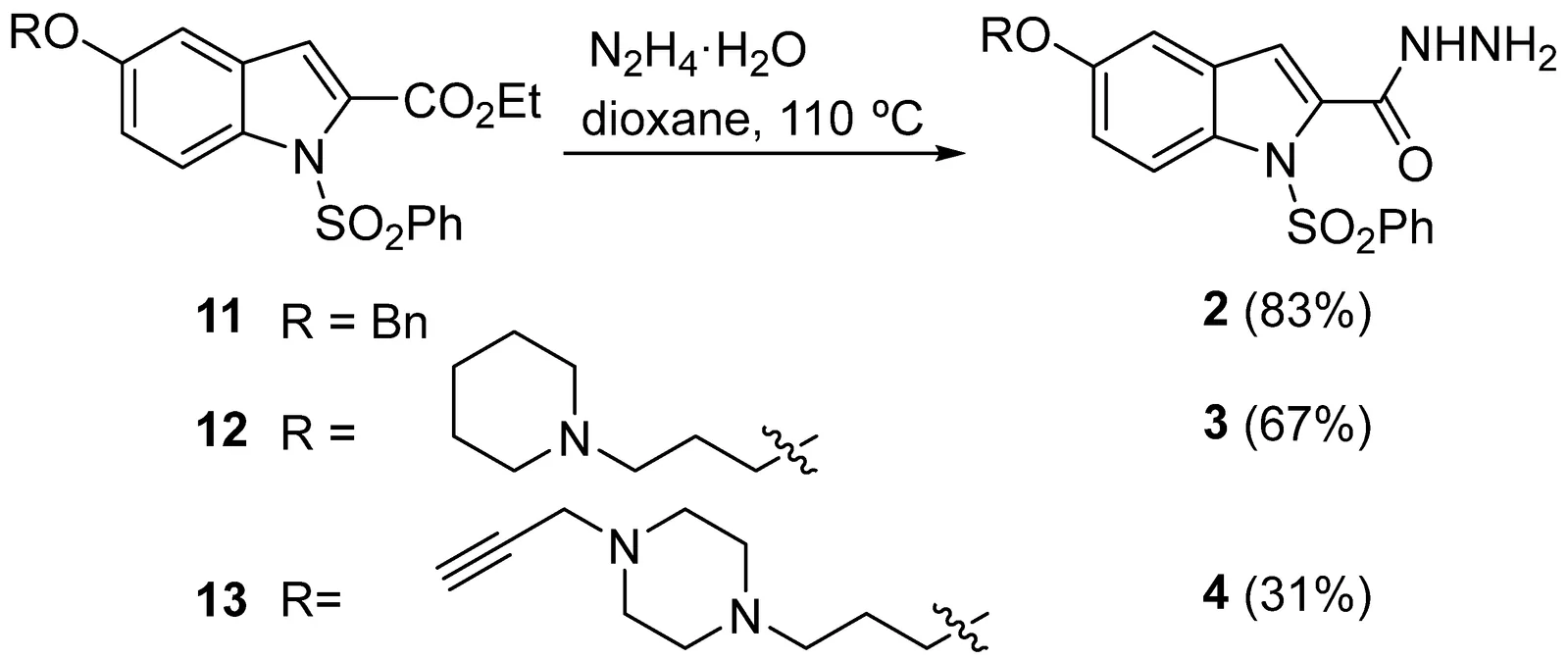
Synthesis of compounds **2**–**4**.

Compounds **1**–**7** were synthesized following the synthetic procedures via intermediates **8**–**16**, as shown in Scheme 1, Scheme 2 and Scheme 3.

Ethyl 5-(3-(piperidin-1-yl)propoxy)-1*H*-indole-2-carboxylate (**9**) and ethyl 5-(3-(4-(prop-2-yn-1-yl)piperazin-1-yl)propoxy)-1*H*-indole-2-carboxylate (**10**) were obtained in 95% and 67% yields, respectively, by *O*-alkylation of commercial ethyl 5-hydroxy-1*H*-indole-2-carboxylate (**8**) with easily available 1-(3-chloropropyl)piperidine chlorhydrate and 1-(3-chloropropyl)-4-(prop-2-yn-1-yl)piperazine, respectively, in the presence of potassium carbonate, in a mixture of CHCl_3_ and water, at 80 °C for 3 days (Scheme 1). Next, commercial ethyl 5-(benzyloxy)-1*H*-indole-2-carboxylate (**8**) and precursors **9** and **10** were treated with benzenesulfonyl chloride and NaH in dry DMF to afford the corresponding *N*(1)-phenylsulfonyl derivatives **11**–**13** in good yields (Scheme 1). Then, these intermediates were reacted with KOH in THF/EtOH to provide the corresponding acids **14**–**16** in satisfactory yields, which were transformed into hydroxamate **1** (from acid **14**) (Scheme 2) or into *o*-aminoanilides **5–7** (Scheme 1) by reacting them with hydroxylamine hydrochloride or 1,2-phenylenediamine, respectively, in the presence of HATU/DIPEA. Finally, hydrazides **2**–**4** were easily obtained from esters **11**–**13** by reacting them with hydrazine hydrate in refluxing dioxane (Scheme 3).

All new compounds showed good analytical and spectroscopic data, in good agreement with the literature and their structures (see Materials and Methods, Supplementary Materials, Content S3).

#### 2.2.2. Biological Evaluation

Regarding the inhibition of ChEs [27], only *N*-(2-aminophenyl)-1-(phenylsulfonyl)-5-(3-(piperidin-1-yl)propoxy)-1*H*-indole-2-carboxamide (**6**) (Figure 3) was active (*h*BuChE: IC_50_ = 0.97 μM) in the low micromolar range and quite selective vs. *h*AChE (IC_50_ = 26.78 μM) (Table 3).

The analysis of the inhibition of HDAC1/6 enzymes [28] showed that only 5-(benzyloxy)-*N*-hydroxy-1-(phenylsulfonyl)-1*H*-indole-2-carboxamide (**1**) was a modest but selective HDA6i (IC_50_ = 3.51 ± 0.61 μM).

Finally, following the 5-HT_6_R radioligand binding assay [26], tested compounds **2**, **3**, **5**, and **6** (Figure 3) showed no affinity (**2**) or very poor affinity [3 (Ki = 31180 nM); 5 (Ki = 26440 nM); 6 (Ki = 22520 nM)]. These results were totally unexpected and prevented us from testing compounds **1**, **4**, and **7,** but they prompted us to carry out molecular modeling for compound **2** docking on ChEs, HDAC1/6, and 5-HT_6_R to justify why no inhibition/affinity was observed for this ligand, as an example of the observed negative results. On the other hand, regarding the observed positive results, we also carried out molecular modeling of compound **6** docking on BuChE. The results are described in the Supplementary Materials (Figures S1–S5 for compound **2**; Figure S6 for compound **6**).

To sum up, and compared with Contilisant (Figure 1), it seemed that in hybrids of type **III**, C2 substitution at the indole core without the *N*-methylpropargyl motif was detrimental for activity. Thus, based on the unexpected results obtained for Contilisant analogs of type **III** (Figure 3), we reconsidered the Contilisant hybrids of type **I** and **II** (Figure 1) for further investigation.

At this point in our project, based on the results for the modulation of 5-HT_6_R shown above in Table 1 and Table 2, and for a project focused on therapeutic agents acting on the central nervous system (CNS), it was mandatory to assess the ability of selected Contilisant+Tubastatin A and Contilisant+Belinostat hybrids and Vorinostat as a reference ligand to cross the blood–brain barrier (BBB).

### 2.3. Parallel Artificial Membrane Permeation Assay—Blood–Brain Barrier (PAMPA-BBB)

To evaluate the brain–brain barrier penetration ability of the different compounds, a PAMPA for BBB was used, following the method described by Di et al. [29]. The in vitro permeability (Pe) of 14 commercially available drugs through a lipid extract of porcine brain membranes, together with that of the test compounds, was determined. Method validation was performed by correlating the experimentally obtained permeability values with those reported in the literature for the same 14 reference drugs (y = 1.589x − 1.306; R^2^ = 0.9402). Based on this equation and considering the permeability limits proposed by Di et al. for blood–brain barrier (BBB) penetration, we defined the following ranges: compounds with high BBB permeability (CNS+), Pe (10^−6^ cm s^−1^) > 5.050; compounds with low BBB permeability (CNS–), Pe (10^−6^ cm s^−1^) < 1.872; and compounds with uncertain BBB permeability (CNS±), 1.872 < Pe (10^−6^ cm s^−1^) < 5.050.

Table 4 shows the permeability results from assayed Contilisant+Tubastatin A hybrids of type I (three different experiments in triplicate) and predicted penetration in the CNS. As shown, ligands MTP89, FRB24, MTP98, MTP165, and MTP155, in decreasing order, were predicted to cross the BBB, whereas the permeability of compounds MTP86, FRB44, MTP109, and MTP195 was borderline, and compound MTP100 was predicted not to be able to cross the BBB.

In Table 4, we also combined the permeability prediction with the relevant in vitro pharmacological data shown in Table 1, or previously reported by us [3], to provide a clear picture of the progress of the project in order to select the ligands to be tested in the next in vivo assays.

Consequently, the most attractive and interesting ligands for further appropriate evaluation were:

1. Hydroxamate MTP89, as the most permeable, predicted to cross the BBB (Exp value: 23.3 ± 4.2, CNS+), being an MSM on HDAC and 5-HT_6_R biological targets [HDAC1 (3.394 μM) [3]; HDAC6 (0.16 μM) [3]; 5-HT_6_R (6.305 μM)].

2. *N*-*n*-Propyl hydrazide FRB24, predicted to cross the BBB (Exp value: 14.5 ± 1.0, CNS+), being an MSM on HDAC and 5-HT_6_R biological targets [HDAC1 (0.087 μM); 5-HT_6_R (5.820 μM)].

3. Hydroxamate MTP98, predicted to cross the BBB (Exp value: 7.5 ± 0.25, CNS+), being an MSM on HDAC and 5-HT_6_R biological targets [HDAC1 (3.99 μM) [3]; HDAC6 (0.299 μM) [3]; 5-HT_6_R (1.543 μM)].

4. *o*-Aminoanilide MTP165, predicted to cross the BBB (Exp value: 6.65 ± 0.3, CNS+), being an MSM on HDAC, *eq*BuChE, H_3_R, and 5-HT_6_R biological targets [HDAC1 (0.24 μM) [3]; *eq*BuChE (0.263 μM); *h*H_3_R (48%); 5-HT_6_R (1.462 μM)], and taking all of these results into consideration, compounds FRB24 and MTP165 were the most balanced.

Table 5 shows the permeability results for assayed Contilisant+Belinostat hybrids of type **II** (three different experiments in triplicate) and the predicted penetration into the CNS. As shown, ligands MTP156 and MTP150, in decreasing order, were predicted to cross the BBB, whereas the permeability of compounds SMD10, MTP142, MTP167, and APP17 was borderline, and compound APP19 was predicted not to be able to cross the BBB.

In Table 5, we combined the permeability prediction with the relevant in vitro pharmacological data shown in Table 2 or previously reported [4].

Consequently, we selected the following ligands for further appropriate in vivo biological evaluation:

1. Hydroxamate MTP156 (Table 2), as the most permeable, predicted to cross the BBB (Exp value: 7.1 ± 0.15, CNS+), being an MSM on HDAC and 5-HT_6_R biological targets [HDAC1 (0.218 μM); HDAC6 (0.335 μM) [4]; 5-HT_6_R (6.122 μM)].

2. Hydroxamate MTP150 (Figure 2), predicted to cross the BBB (Exp value: 6.5 ± 0.7, CNS+), being an MSM on HDAC, *eq*BuChE, and 5-HT_6_R biological targets [HDAC1 (0.019 μM); HDAC6 (0.04 μM); *h*AChE (19.1 μM); *h*BuChE (27.1 μM) [4]; 5-HT_6_R (13.580 μM)].

Overall, comparing the most significant results of Contilisant+Tubastatin A hybrids FRB24 (Figure 2) and MTP165 (Table 1), with those of Contilisant+Belinostat hybrids MTP156 and MTP150, the most balanced seemed to be hydroxamate MTP150.

Very interestingly, molecular docking of compound MTP150 on HDAC1/6 confirmed the experimental results (Supplementary Materials, Figures S7–S10), and in silico ADME predictions suggested its druggability (Supplementary Materials, Table S1). Consequently, ligand MTP150 was selected for further in vivo tests in suitable ND models.

### 2.4. Evaluation of the Therapeutic Potential of Compound MTP150 in C. elegans in an In Vivo Models of AD, PD, and Huntington’s Disease

To explore the neuroprotective potential of MTP150 across neurodegenerative diseases, we examined its effects on *C. elegans* transgenic models of PD [30,31], Huntington’s disease (HD) [32], and AD. HD involves altered serotonin receptor expression and HDAC dysfunction, both of which contribute to neurodegeneration [33,34].

These strains are well-established for studying neurodegeneration, as PD models exhibit α-synuclein aggregation and dopaminergic neuron loss, HD models express polyglutamine (polyQ) expansions linked to Huntington’s pathology, and AD models display Aβ aggregation and paralysis.

**Figure 4:**
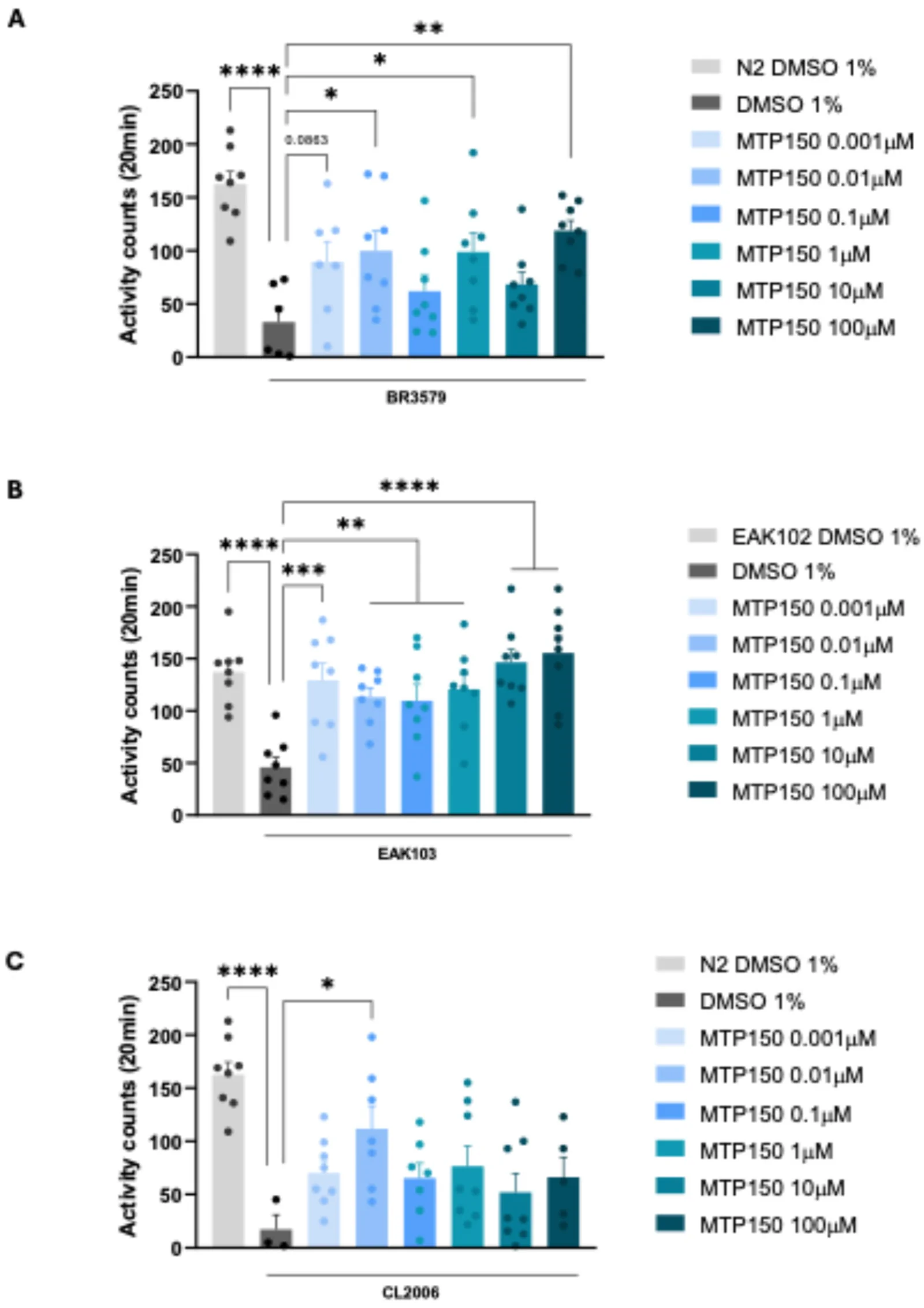
Improvement in the locomotor activity of *C. elegans* transgenic models after MTP150 treatment. (**A**) PD strain model (BR3579), (**B**) HD strain model (EAK103), and (**C**) AD strain model (CL2006) activity counts for 20 min. Values are expressed as the mean  ±  SEM. Groups were statistically compared using one-way ANOVA analysis followed by Dunnett’s post hoc test. n  =  8 wells per group; 25–35 worms/well. * *p*  <  0.05; ** *p*  <  0.01; *** *p* < 0.001; **** *p* < 0.0001.

As shown in Figure 4, we first observed a significant increase in the activity counts of the BR3579 strain at 0.01 µM, 1 µM, and 100 µM doses. This strain is an early-stage PD model characterized by α-syn aggregates in the muscle.

Regarding the HD model, we studied whether the Htt513 polyQ length-dependent reduced motility of the EAK103 strain was improved after MTP150 treatment. We demonstrated a significant dose-dependent response, showing the greatest effects at 10 µM and 100 µM doses.

Finally, we performed an analysis of the CL2006 strain of AD, which expresses human Aβ_1–42_ under a muscle-specific promoter and develops progressive adult-onset paralysis in response to Aβ_1–42_ aggregation [35]. In this case, we found a significant increase in activity counts at 0.01 µM.

Overall, MTP150 treatment enhanced locomotor activity in *C. elegans*, showing substantial effects across different disease models and concentrations. These results demonstrate MTP150’s broad neuroprotective capacity across distinct neurodegenerative mechanisms in *C. elegans*, highlighting its potential to mitigate protein aggregation and neuronal dysfunction in PD, HD, and AD.

Based on the promising results obtained in *C. elegans* models of several NDs, and to further investigate the neuroprotective activity of MTP150, we evaluated its therapeutic potential in *Drosophila* and cell models of PD based on the inactivation of the *DJ-1* gene, the mutations of which are causative for a recessive familial form of PD [36]. Both PD models have been extensively used in the identification of potential therapeutic compounds for this disease [37].

### 2.5. Evaluation of the Therapeutic Potential of MTP150 in Drosophila and Human Cell Models of PD

Flies mutant for the *DJ-1β* gene, the fly ortholog of human *DJ-1,* display PD-like symptoms, including motor deficits, high oxidative stress levels, and mitochondrial dysfunction [3,37,38].

To assess the therapeutic potential of MTP150 in the in vivo *Drosophila* model of PD, *DJ-1β* mutant flies were cultured in medium supplemented with either 1 μM or 10 μM MTP150 during larval development, and flies were collected 5 days after eclosion. Several experiments were carried out to determine whether MTP150 administration was able to suppress PD-like phenotypes in model flies.

**Figure 5:**
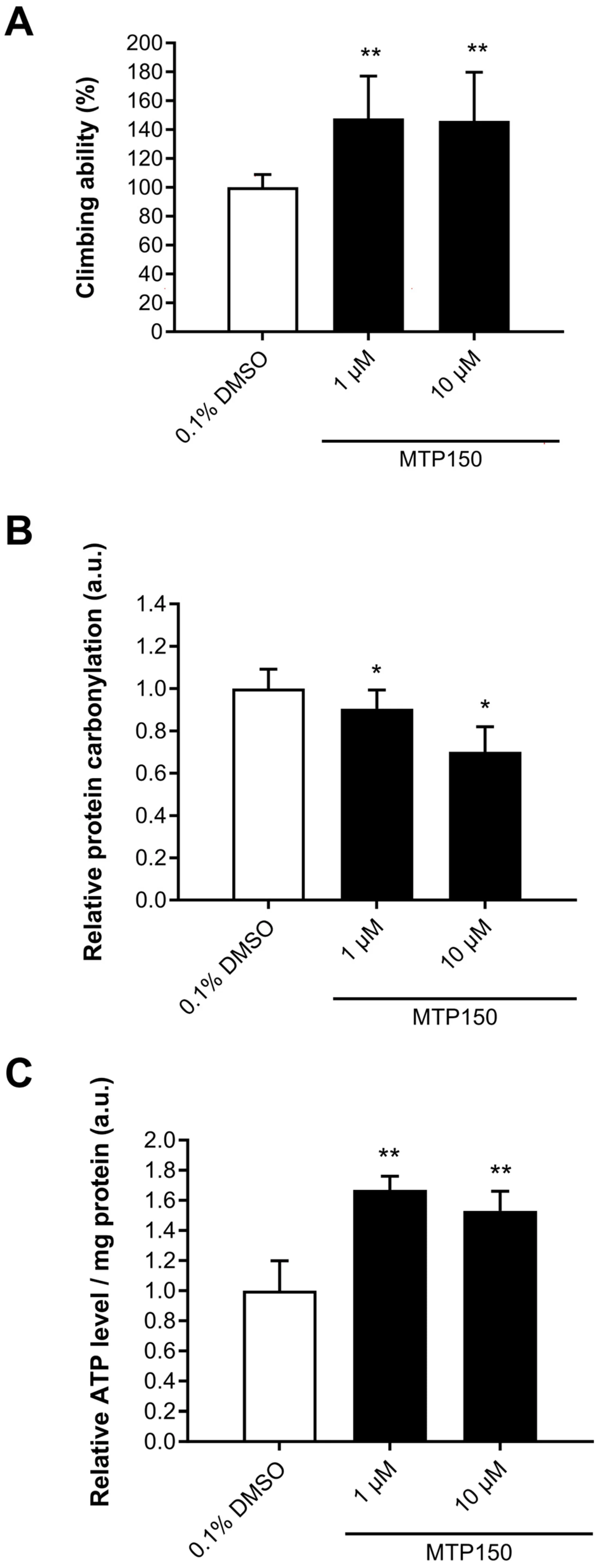
Effect of MTP150 treatment on phenotypes exhibited by *DJ-1β* mutant flies. (**A**) Climbing ability, (**B**) protein carbonylation levels, and (**C**) ATP levels of 5-day-old *DJ-1β* mutant flies treated with 1 and 10 μM MTP150. In all cases, results were referred to data obtained in *DJ-1β* mutant flies treated with vehicle (0.1% DMSO). Values are expressed as the mean  ±  SD from three independent experiments, in which three biological replicates were used. Groups were statistically compared using one-way ANOVA analysis followed by Dunnet’s post hoc test (* *p* < 0.05, ** *p* < 0.01).

Climbing assays were performed to evaluate the motor function of *DJ-1β* mutant flies after MTP150 treatment. A significant improvement in motor performance was found in 5-day-old *DJ-1β* mutant flies treated with MTP150 at both doses when compared to flies treated with vehicle (0.1% DMSO) (Figure 5A).

As an oxidative stress marker, we measured protein carbonylation levels in PD model flies treated with MTP150. It was shown that this post-translational oxidative modification had a causative role in motor deficits exhibited by PD model flies [37]. Our results showed that 5-day-old *DJ-1β* mutant flies supplemented with either 1 or 10 μM MTP150 displayed a significant reduction in protein carbonylation levels when compared to vehicle-treated flies (Figure 5B).

Finally, we analyzed ATP levels in 5-day-old *DJ-1β* mutant flies supplemented with MTP150 during development. Flies treated with both MTP150 doses exhibited a significant increase in ATP levels when compared to those treated with vehicle (Figure 5C), demonstrating that MTP150 supplementation was also able to restore mitochondrial function.

Taken together, our results confirmed the therapeutic potential of MTP150 in PD model flies. Among the concentrations used, 10 μM appeared to be the most effective since it was able to ameliorate all PD-like phenotypes evaluated in *DJ-1β* mutant flies.

Although *Drosophila* has been shown to be an excellent model organism for identifying potential treatments for human diseases, it was essential to confirm whether candidate compound was also beneficial in mammalian cell systems [39].

**Figure 6:**
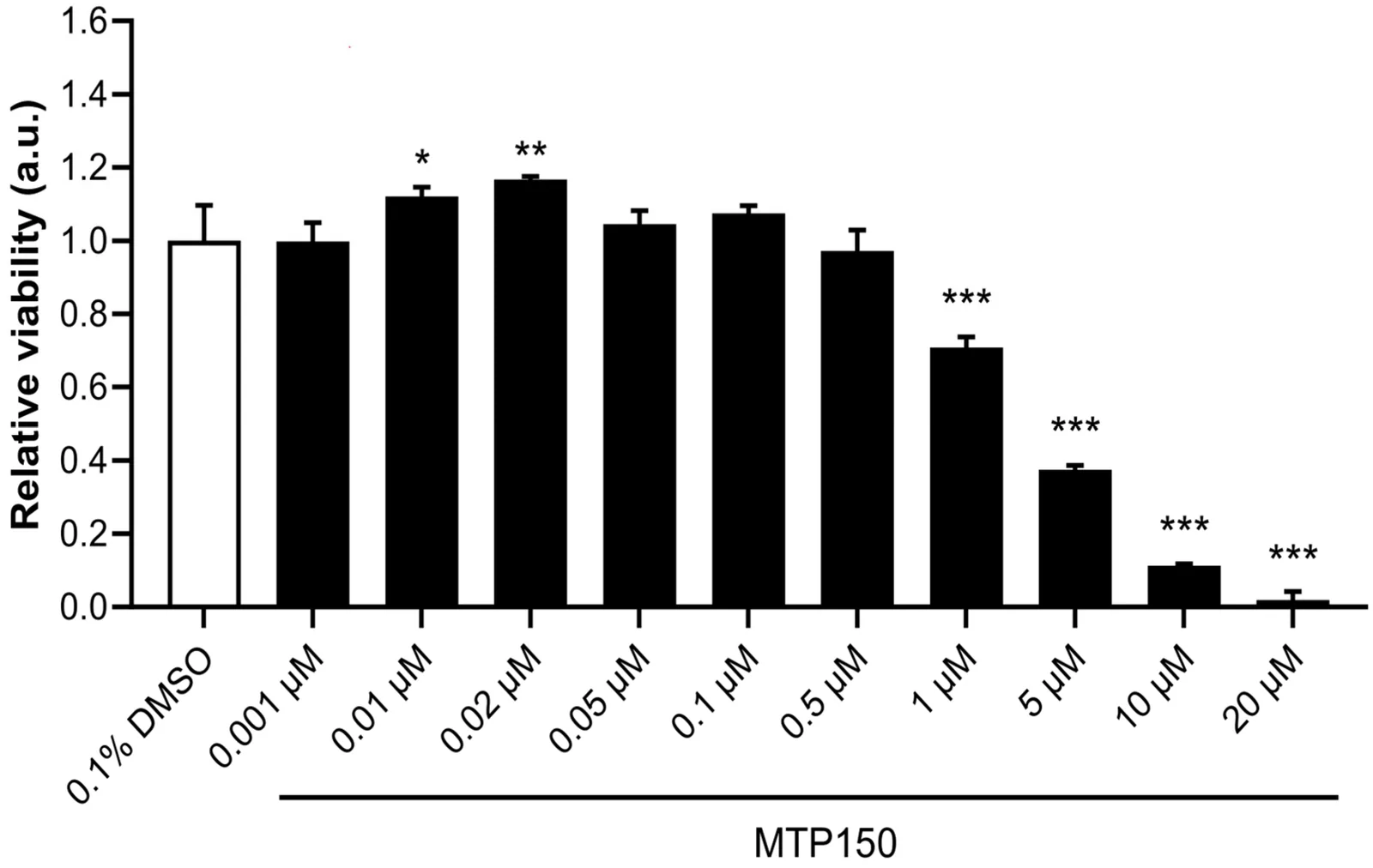
Effect of MTP150 on the viability of control SH-SY5Y cells. The MTT assay was performed to measure the viability of *pLKO.1* control cells either treated with vehicle (0.1% DMSO) or with ten different concentrations of the compound. Results were normalized to data obtained in vehicle-treated cells. Values are expressed as the mean  ±  SD from three independent experiments in which three biological replicates were used. Groups were statistically compared using one-way ANOVA analysis followed by Dunnet’s post hoc test (* *p* < 0.05, ** *p* < 0.01, *** *p* < 0.001).

Thus, we assessed the therapeutic potential of MTP150 in *DJ-1-*deficient human SH-SY5Y cells, which exhibit reduced viability when cultured under oxidative stress conditions [37]. To demonstrate the neuroprotective effect of MTP150, we needed to show that this compound was able to increase the viability of PD model cells under such conditions. To do so, we first performed a toxicity assay by pretreating SH-SY5Y control cells (*pLKO.1* cells) with different concentrations of MTP150 from 0.001 to 20 μM to investigate the possible cytotoxicity of this compound. Our results showed that cell viability was only compromised at concentrations higher than >1 μM (Figure 6).

**Figure 7:**
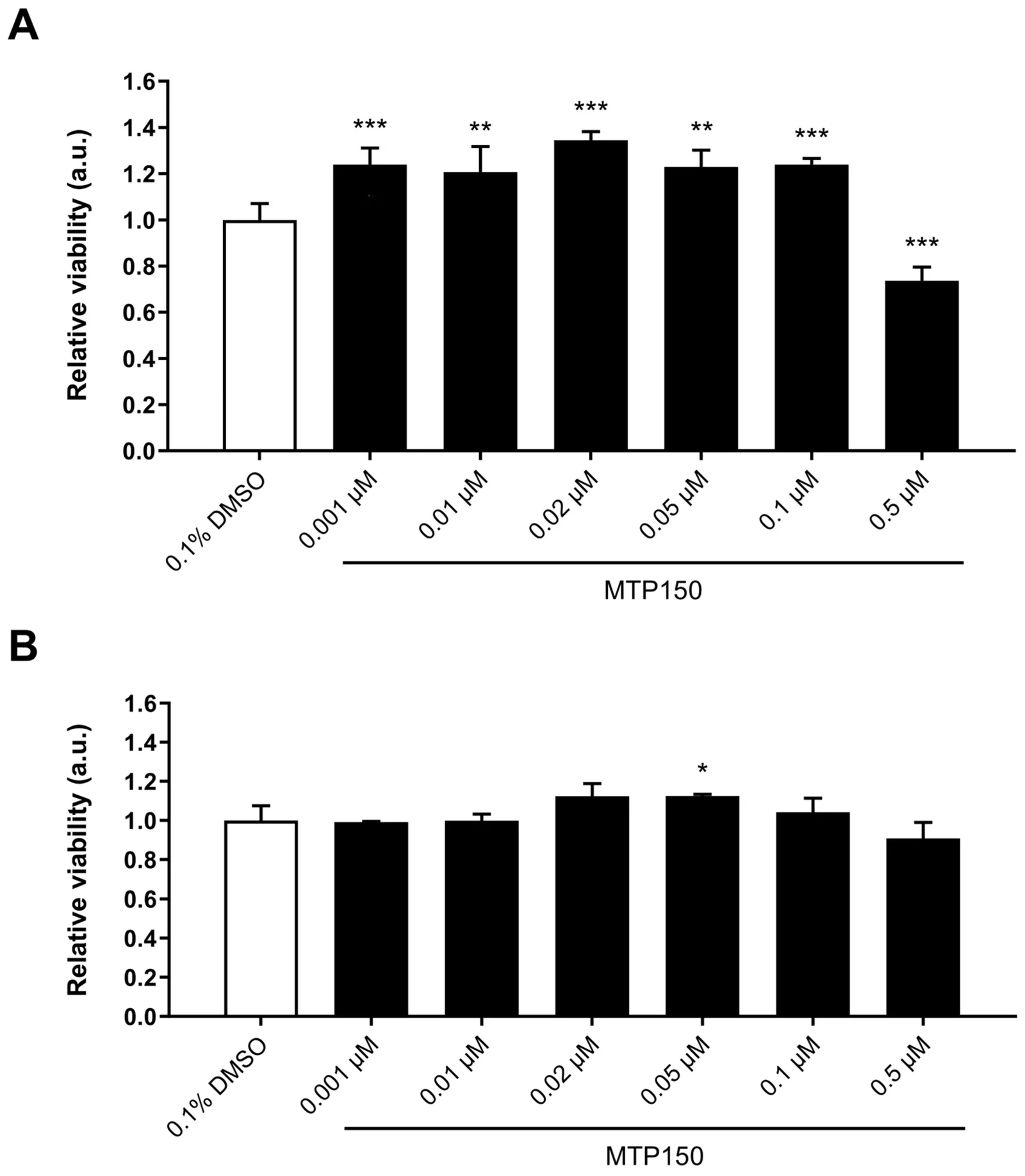
Effect of MTP150 on viability of *DJ-1*-deficient and control SH-SY5Y cells. The MTT assay was performed to measure the viability of (**A**) *DJ-1*-deficient and (**B**) pLKO.1 control cells grown under oxidative stress conditions (induced with 100 µM H_2_O_2_) either treated with vehicle (0.1% DMSO) or with different concentrations of the compound. Results were normalized to data obtained in vehicle-treated cells. Values are expressed as the mean  ±  SD from three independent experiments in which three biological replicates were used. Groups were statistically compared using one-way ANOVA analysis followed by Tukey’s post hoc test (* *p* < 0.05, ** *p* < 0.01, *** *p* < 0.001).

Thus, to determine whether MTP150 was able to exert a neuroprotective effect in our human cell PD model, *DJ-1-*deficient cells were pretreated with nontoxic concentrations of MTP150 before being cultured under oxidative stress conditions induced with 100 µM H_2_O_2_. Our results showed that pretreatments with 0.001, 0.01, 0.02, 0.05, and 0.1 μM were able to significantly suppress H_2_O_2_-induced cell death in this in vitro model of PD (Figure 7A). As shown in Figure 7B, the same concentrations of MTP150 did not have any effect on the viability of *pLKO.1* control cells cultured under the same oxidative conditions (except for 0.05 μM), demonstrating the clear specificity of its effect in PD model cells.

Taken together, our results supported the neuroprotective effect of MTP150 in *Drosophila* and human cell models of PD based on *DJ-1* deficiency. However, the difference in beneficial MTP150 concentrations observed between human cells and *Drosophila* represents a limitation of this study, as it likely reflects the inherent distinction between an in vitro system and a whole organism.

## 3. Materials and Methods

### 3.1. Synthesis General Methods

Reactions were monitored via TLC using precoated silica gel aluminum plates containing a fluorescent indicator (Merck, 5539, Madrid, Spain). Detection was performed under UV light (254 nm), followed by charring with sulfuric-acetic acid spray, 1% aqueous potassium permanganate solution, or 0.5% phosphomolybdic acid in 95% EtOH. Anhydrous Na_2_SO_4_ was used to dry organic solutions during work-ups, and the removal of solvents was carried out under vacuum with a rotary evaporator. Flash column chromatography was performed using silica gel 60 (230–400 mesh, Merck). Melting points were determined on a Kofler block and are uncorrected. IR spectra were obtained on a Perkin-Elmer Spectrum One spectrophotometer (Madrid, Spain). ^1^H NMR spectra were recorded using a Varian VXR-200S spectrometer (Madrid, Spain), with tetramethylsilane as the internal standard, and ^13^C NMR spectra were recorded using a Bruker WP-200-SY (Madrid, Spain). All assignments for protons and carbons were in agreement with 2D COSY, HSQC, HMBC, and 1D NOESY spectra. The purity of compounds was verified through elemental analysis conducted on a Carlo Erba EA 1108 apparatus (Madrid, Spain) and confirmed to be ≥95%.

*General Procedure for Alkylation Reaction (A).* A mixture of substituted indole (1.0 equiv) and appropriate amounts of amine hydrochloride (1.5 equiv) and potassium carbonate (3 equiv) was dissolved in CHCl_3_ (2.5 mL) and water (1.0 mL). Then, the reaction mixture was stirred at 80 °C for 3 d. After completion of the reaction (TLC analysis), the mixture was extracted with DCM (3 × 20 mL). The combined organic extracts were washed with sodium bicarbonate (15 mL), dried over anhydrous magnesium sulfate, and evaporated. The product was purified using column chromatography. *General Method for the Synthesis of N-Sulfonamides (B).* To a solution of the corresponding indole (1.0 mmol) in anhydrous DMF (6 mL), cooled at 0 °C, sodium hydride (2.0 mmol, 60% in oil) was added and the mixture was stirred for 30 min. Then, the arylsulfonyl chloride (1.1 mmol) was added and the mixture was stirred at room temperature (rt) for 24 h. After completion of the reaction (TLC analysis), distilled water (20 mL) was added and the mixture was extracted with AcOEt (3 × 20 mL). The combined organic extracts were washed with water (3 × 20 mL), dried over anhydrous magnesium sulfate, and evaporated. The crude product was purified using column chromatography. *General Method for Acid Synthesis (C).* A mixture of the ester (1.0 equiv), 2N KOH (4.0 equiv), THF (10 mL), and ethanol (10 mL) was stirred at rt for 4 h, then it was quenched on crushed ice and made acidic with 37% HCl. Ethyl acetate (3 × 20 mL) was added, and the organic layer was separated, washed with brine (3 × 20 mL), and dried over anhydrous MgSO_4_. After the removal of MgSO_4_ by filtration, the filtrate was concentrated *under vacuum.* The residue was purified via flash chromatography over silica gel. *General Method for Amide Synthesis (D). N*,*N*-Diisopropylethylamine (DIPEA) (2.5–3.5 mmol) and 1-[bis(dimethylamino)methylene]-1*H*-1,2,3-triazolo[4,5-b]pyridinium 3-oxid hexafluorophosphate (HATU) (1.0 mmol) were sequentially added at room temperature to a solution of the corresponding acid (1.0 mmol) in dry DMF (4 mL). The reaction was stirred for 15 min and then hydroxylamine hydrochloride or 1,2-phenylenediamine was added (1.0 mmol). After completion of the reaction (TLC analysis), the crude product was precipitated in a solution of water/brine (4:1), filtered, and dried. Then, the solid was purified using column chromatography. *General Procedure for Hydrazide Synthesis (E).* To a solution of hydrazine monohydrate (5.0 equiv) in dioxane (5 mL), substituted indole (1.0 equiv) was added. The resulting mixture was stirred at 110 °C overnight. After completion of the reaction (TLC analysis), the residue was added to brine solution and extracted with DCM (3 × 20 mL). The combined organic layer was dried over anhydrous magnesium sulfate, filtered, and concentrated in vacuum. The product was purified using column chromatography.

#### 3.1.1. Ethyl 5-(3-(Piperidin-1-yl)propoxy)-1H-indole-2-carboxylate (**9**)

Following *General Method A*, the reaction of a solution of commercial ethyl 5-hydroxy-1H-indole-2-carboxylate (**8**) (102.61 mg, 0.5 mmol) in CHCl_3_ (2.5 mL) and water (0.5 mL) with commercial 1-(3-chloropropyl)piperidine chlorhydrate (148.6 mg, 0.75 mmol) and potassium carbonate (414.6 mg, 3 mmol), after flash chromatography over silica gel using DCM/methanol (2%), gave product **9** (150.4 mg, 95%) as a white solid: IR (cm^−1^) ν 3319 (N-H), 1231 (C-O-C); ^1^H NMR (300 MHz, CDCl_3_) δ 8.89 (s, 1H), 7.30 (dt, J = 8.9, 0.8 Hz, 1H), 7.13 (dd, J = 2.1, 1.0 Hz, 1H), 7.08 (dt, J = 2.4, 0.7 Hz, 1H), 6.91 (dd, J = 8.9, 2.4 Hz, 1H), 4.33 (q, J = 7.1 Hz, 2H), 3.96 (t, J = 6.3 Hz, 2H), 2.55 (t, J = 7.8 Hz, 2H), 2.51–2.38 (m, 4H), 2.12–1.95 (m, 2H), 1.64 (p, J = 5.5 Hz, 4H), 1.51–1.44 (m, 2H), 1.34 (t, J = 7.1 Hz, 3H); ^13^C NMR (75 MHz, CDCl_3_) δ 160.9, 153.0, 131.2, 126.8, 116.3, 111.6, 107.1, 102.7, 66.0, 59.9, 55.1, 53.6, 25.7, 24.8, 23.3, 13.4; HRMS (ESI): Calcd for C_19_H_26_N_2_O_3_^+^ [M + H]^+^: 331.2016. Found: 331.2012.

#### 3.1.2. Ethyl 5-(3-(4-(Prop-2-yn-1-yl)piperazin-1-yl)propoxy)-1H-indole-2-carboxylate (**10**)

Following *General Method A*, the reaction of a solution of commercial ethyl 5-hydroxy-1*H*-indole-2-carboxylate (**8**) (600 mg, 2.92 mmol) in CHCl_3_ (7.3 mL) and water (3 mL) with commercial 1-(3-chloropropyl)-4-(prop-2-yn-1-yl)piperazine (800 mg, 4.39 mmol) and potassium carbonate (1.2 g, 8.77 mmol), after flash chromatography over silica gel using DCM/methanol (2%), gave product **10** (726 mg, 67%) as a white solid: IR (cm^−1^) ν3304 (N-H), 1672 (C=O), 1214 (C-O-C); ^1^H NMR (400 MHz, CDCl_3_) δ 9.14 (s, 1H), 7.30 (dt, *J* = 9.0, 0.8 Hz, 1H, H7), 7.13 (dd, *J* = 2.1, 0.9 Hz, 1H, H3), 7.08 (d, *J* = 2.4 Hz, 1H, H4), 6.99 (dd, *J* = 8.9, 2.4 Hz, 1H, H6), 4.41 (q, *J* = 7.1 Hz, 2H), 4.04 (t, *J* = 6.4 Hz, 2H), 3.31 (d, *J* = 2.5 Hz, 2H), 2.91–2.44 (m, 10H), 2.25 (t, *J* = 2.4 Hz, 1H), 2.11–1.88 (m, 2H), 1.41 (t, *J* = 7.1 Hz, 3H); ^13^C NMR (101 MHz, CDCl_3_) δ 162.0, 153.9, 132.2, 127.8, 127.8, 117.3 (C6), 112.7 (C7), 108.1 (C3), 103.6 (C4), 78.8, 73.2 (CH), 66.8 (CH_2_), 60.9 (CH_2_), 55.2 (2CH_2_), 53.0 (2CH_2_), 51.8 (CH_2_), 46.8 (CH_2_), 26.8 (CH_2_), 14.4 (CH_3_); HRMS (ESI): Calcd for C_21_H_28_N_3_O_3_^+^ [*M* + H]^+^: 370.2125. Found: 370.2126.

#### 3.1.3. Ethyl 5-(Benzyloxy)-1-(phenylsulfonyl)-1H-indole-2-carboxylate (**11**)

Following *General Method B*, the reaction of a solution of commercial ethyl 5-(benzyloxy)-1*H*-indole-2-carboxylate (**8**) (700 mg, 2.37 mmol) in dry DMF (33 mL) with NaH (190 mg, 4.74 mmol) and benzenesulfonyl chloride (0.33 mL, 2.61 2.58 mmol), after flash chromatography of the residue using hexane/AcOEt (10%) as the eluent, afforded compound **11** (680 mg, 66%) as a yellow solid: mp 101–103 °C; IR (cm^−1^) ν 1726 (C=O), 1368 (O=S=O), 1205 (C-O-C); ^1^H NMR (400 MHz, CDCl_3_) δ 8.02 (d, *J* = 9.2 Hz, 1H, H7), 8.00–7.96 (m, 1H), 7.61–7.29 (m, 9H), 7.13 (dd, *J* = 9.2, 2.5 Hz, 1H, H6), 7.09 (d, *J* = 0.8 Hz, 1H, H3), 7.04 (d, *J* = 2.5 Hz, 1H, H4), 5.08 (s, 2H), 4.40 (q, *J* = 7.2 Hz, 2H), 1.39 (t, *J* = 7.2 Hz, 3H); ^13^C NMR (101 MHz, CDCl_3_) δ 161.2, 155.9, 138.5, 136.7, 133.7 (CH_Ar_), 133.1, 132.5, 129.2, 128.9 (2CH_Ar_), 128.6 (2CH_Ar_), 128.1 (CH_Ar_), 127.5 (2CH_Ar_), 127.2 (2CH_Ar_), 117.2 (C6), 117.0 (C3), 116.4 (C7), 105.4 (C4), 70.5 (CH_2_), 61.9 (CH_2_), 14.1 (CH_3_); HRMS (ESI): Calcd for C_24_H_22_NO_5_S^+^ [*M* + H]^+^: 436.1213. Found: 436.1208.

#### 3.1.4. Ethyl 1-(Phenylsulfonyl)-5-(3-(piperidin-1-yl)propoxy)-1H-indole-2-carboxylate (**12**)

Following *General Method B*, the reaction of a solution of ethyl 5-(3-(piperidin-1-yl)propoxy)-1*H*-indole-2-carboxylate (**9**) (380 mg, 1.15 mmol) in dry DMF (16 mL) with NaH (92 mg, 2.30 mmol) and benzenesulfonyl chloride (0.2 mL, 1.27 mmol), after flash chromatography of the residue using DCM/methanol (2%) as the eluent, provided compound **12** (450 mg, 82%) as a yellow oil: IR (cm^−1^) ν 1726 (C=O), 1368 (O=S=O), 1209 (C-O-C); ^1^H NMR (400 MHz, CDCl_3_) δ 8.00 (d, *J* = 9.2 Hz, 1H, H7), 7.98–7.94 (m, 2H), 7.60–7.52 (m, 1H), 7.49–7.44 (m, 2H), 7.09 (d, *J* = 0.8 Hz, 1H, H3), 7.02 (dd, *J* = 9.2, 2.5 Hz, 1H, H6), 6.95 (d, *J* = 2.5 Hz, 1H, H4), 4.40 (q, *J* = 7.2 Hz, 2H), 4.03 (t, *J* = 6.1 Hz, 2H), 2.79–2.70 (m, 6H), 2.19–2.15 (m, 2H), 1.82–1.76 (m, 4H), 1.56–1.54 (m, 2H), 1.39 (t, *J* = 7.2 Hz, 3H); ^13^C NMR (101 MHz, CDCl_3_) δ 161.2, 155.9, 138.3, 133.7 (CH_Ar_), 133.0, 132.5, 129.2, 128.9 (2CH_Ar_), 127.2 (2CH_Ar_), 117.0 (C3), 116.9 (C6), 116.4 (C7), 104.8 (C4), 66.3 (CH_2_), 61.9 (CH_2_), 55.7 (CH_2_), 54.2 (2CH_2_), 25.6 (CH_2_), 24.6 (2CH_2_), 23.5 (CH_2_), 14.1 (CH_3_). HRMS (ESI): Calcd for C_25_H_31_N_2_O_5_S^+^ [*M* + H]^+^: 471.1948. Found: 471.1949.

#### 3.1.5. Ethyl 1-(Phenylsulfonyl)-5-(3-(4-(prop-2-yn-1-yl)piperazin-1-yl)propoxy)-1H-indole-2-carboxylate (**13**)

Following *General Method B*, the reaction of a solution of ethyl 5-(3-(4-(prop-2-yn-1-yl)piperazin-1-yl)propoxy)-1*H*-indole-2-carboxylate (**10**) (580 mg, 1.57 mmol) in dry DMF (22 mL) with NaH (126 mg, 3.14 mmol) and benzenesulfonyl chloride (0.2 mL, 1.73 mmol), after flash chromatography of the residue using DCM/methanol (2%) as the eluent, produced compound **13** (590 mg, 74%) as a yellow solid: mp 71–73 °C; IR (cm^−1^) ν 1726 (C=O), 1368 (O=S=O), 1209 (C-O-C); ^1^H NMR (400 MHz, CDCl_3_) δ 8.00 (d, *J* = 9.2 Hz, 1H, H7), 7.98–7.93 (m, 2H), 7.59–7.52 (m, 1H), 7.50–7.40 (m, 2H), 7.08 (s, 1H, H3), 7.04 (dd, *J* = 9.2, 2.5 Hz, 1H, H6), 6.97 (d, *J* = 2.5 Hz, 1H, H4), 4.40 (q, *J* = 7.2 Hz, 2H), 4.02 (t, *J* = 6.3 Hz, 2H), 3.31 (d, *J* = 2.5 Hz, 2H), 2.65–2.56 (m, 10H), 2.26 (t, *J* = 2.5 Hz, 1H), 2.01 (p, *J* = 6.7 Hz, 2H), 1.39 (t, *J* = 7.2 Hz, 3H); ^13^C NMR (101 MHz, CDCl_3_) δ 161.2, 156.2, 138.4, 133.7 (CH_Ar_), 132.9, 132.5, 129.2, 128.9 (2CH_Ar_), 127.2 (2CH_Ar_), 117.0 (C6), 117.0 (C3), 116.4 (C7), 104.9 (C4), 78.7, 73.3 (CH), 66.6 (CH_2_), 61.9 (CH_2_), 55.0 (2CH_2_), 53.0 (2CH_2_), 51.7 (CH_2_), 46.8 (CH_2_), 26.6 (CH_2_), 14.1 (CH_3_); HRMS (ESI): Calcd for C_27_H_32_N_3_O_5_S^+^ [*M* + H]^+^: 510.2057. Found: 510.2057.

#### 3.1.6. 5-(Benzyloxy)-1-(phenylsulfonyl)-1H-indole-2-carboxylic Acid (**14**)

Following *General Method C*, the reaction of a solution of ethyl 5-(benzyloxy)-1-(phenylsulfonyl)-1*H*-indole-2-carboxylate (**11**) (200 mg, 0.46 mmol) in a mixture of THF/EtOH (1:1) (1.8 mL) with 2N KOH (0.85 mL, 1.84 mmol), after flash chromatography of the residue using DCM/methanol/NH_4_OH (90:9:1) as the eluent, gave compound **14** (157 mg, 84%) as a white solid: mp 132-4 °C; IR (cm^−1^) ν 2925 (COO-H), 1688 (C=O), 1371 (O=S=O), 1212 (C-O-C); ^1^H NMR (300 MHz, DMSO-d_6_) δ 8.25–8.22 (m, 2H), 7.78 (d, *J* = 9.1 Hz, 1H, H7), 7.62 (d, *J* = 7.3 Hz, 1H), 7.56–7.50 (m, 2H), 7.46–7.28 (m, 5H), 7.07 (d, *J* = 2.6 Hz, 1H, H4), 6.91 (dd, *J* = 9.1, 2.6 Hz, 1H, H6), 6.45 (s, 1H, H3), 5.06 (s, 2H) (the signal for “COO*H*” were not detected); ^13^C NMR (101 MHz, DMSO-d_6_) δ 163.7, 154.9, 138.7, 137.2, 133.6 (CH_Ar_), 130.6, 129.7, 128.9 (2CH_Ar_), 128.4 (2CH_Ar_), 128.4, 127.8 (CH_Ar_), 127.7 (2CH_Ar_), 127.5 (2CH_Ar_), 115.1 (C7), 113.1 (C6), 106.9 (C3), 104.7 (C4), 69.5 (CH_2_); HRMS (ESI): Calcd for C_22_H_18_NO_5_S^+^ [*M* + H]^+^: 408.0900. Found: 408.0904.

#### 3.1.7. 1-(Phenylsulfonyl)-5-(3-(piperidin-1-yl)propoxy)-1H-indole-2-carboxylic Acid (**15**)

Following *General Method C*, the reaction of a solution of ethyl 1-(phenylsulfonyl)-5-(3-(piperidin-1-yl)propoxy)-1*H*-indole-2-carboxylate (**12**) (510 mg, 1.08 mmol) in a mixture of THF/EtOH (1:1) (4.4 mL) with 2N KOH (2 mL, 4.34 mmol), after flash chromatography of the residue using DCM/methanol/NH_4_OH (90:9:1) as the eluent, afforded compound **15** (380 mg, 79%) as a white solid: mp 125-7 °C; IR (cm^−1^) ν 2947 (COO-H), 1600 (C=O), 1361 (O=S=O), 1213 (C-O-C); ^1^H NMR (400 MHz, DMSO-d_6_) δ 8.04–7.86 (m, 3H), 7.76–7.65 (m, 1H), 7.65–7.51 (m, 2H), 7.31 (s, 1H, COO*H*), 7.25 (s, 1H, H3), 7.17 (d, *J* = 2.6 Hz, 1H, H4), 7.07 (dd, *J* = 9.2, 2.6 Hz, 1H, H6), 4.06 (t, *J* = 5.9 Hz, 2H), 3.50–3.47 (m, 2H), 3.30–3.18 (m, 2H), 2.99–2.81 (m, 2H), 2.13 (dq, *J* = 11.4, 5.9 Hz, 2H), 1.84–1.81 (m, 2H), 1.74–1.33 (m, 4H); ^13^C NMR (101 MHz, DMSO-d_6_) δ 161.9, 155.5, 137.3, 134.6 (CH_Ar_), 133.7, 131.9, 129.5 (2CH_Ar_), 129.4, 127.0 (2CH_Ar_), 116.6 (C6), 116.2 (C3), 116.1 (C7), 105.4 (C4), 65.3 (CH_2_), 53.4 (CH_2_), 52.5 (2CH_2_), 23.5 (CH_2_), 22.7 (2CH_2_), 21.3 (CH_2_) (signal for 158.4 (q, *J* = 38.2 Hz) and 115.2 (q, *J* = 288.7 Hz) correspond to “*C*F_3_*C*OOH” added doing to preparation of the sample); HRMS (ESI): Calcd for C_23_H_26_N_2_O_5_S^+^ [*M* + H]^+^: 443.1635. Found: 443.1635.

#### 3.1.8. 1-(Phenylsulfonyl)-5-(3-(4-(prop-2-yn-1-yl)piperazin-1-yl)propoxy)-1H-indole-2-carboxylic Acid (**16**)

Following *General Method C*, the reaction of a solution of ethyl 1-(phenylsulfonyl)-5-(3-(4-(prop-2-yn-1-yl)piperazin-1-yl)propoxy)-1*H*-indole-2-carboxylate (**13**) (610 mg, 1.30 mmol) in a mixture of THF/EtOH (1:1) (5.2 mL) with 2N KOH (2.4 mL, 5.29 mmol), after flash chromatography of the residue using DCM/methanol/NH_4_OH (85:13.5:1.5) as the eluent, provided compound **16** (447 mg, 72%) as a white solid: mp > 230 °C; IR (cm^−1^) ν 3285 (COO-H), 1609 (C=O), 1354 (O=S=O), 1223 (C-O-C); ^1^H NMR (400 MHz, DMSO-d_6_) δ 8.00–7.88 (m, 3H), 7.73–7.65 (m, 1H), 7.62–7.58 (m, 2H), 7.25 (s, 1H, H3), 7.17 (d, *J* = 2.5 Hz, 1H, H4), 7.07 (dd, *J* = 9.2, 2.5 Hz, 1H, H6), 4.06 (t, *J* = 5.9 Hz, 2H), 3.72 (d, *J* = 2.5 Hz, 2H), 3.64–2.97 (m, 10H), 3.16 (s, 1H), 2.16–2.09 (m, 2H) (signal for “COO*H*” was not detected); ^13^C NMR (101 MHz, DMSO-d_6_) δ 161.9, 155.4, 137.3, 134.5 (CH_Ar_), 133.7, 131.8, 129.5 (2CH_Ar_), 129.3, 127.0 (2CH_Ar_), 116.5 (C6), 116.2 (C3), 116.1 (C7), 105.4 (C4), 78.6 (CH), 76.2, 65.1 (CH_2_), 53.1 (CH_2_), 50.0 (2CH_2_), 47.7 (2CH_2_), 44.9 (CH_2_), 23.5 (CH_2_) (signal for 158.4 (q, *J* = 37.6 Hz) and 115.4 (q, *J* = 289.8 Hz) correspond to “*C*F_3_*C*OOH” added doing to preparation of the sample); HRMS (ESI): Calcd for C_25_H_28_N_3_O_5_S^+^ [*M* + H]^+^: 482.1744. Found: 482.1736.

#### 3.1.9. 5-(Benzyloxy)-N-hydroxy-1-(phenylsulfonyl)-1H-indole-2-carboxamide (**1**)

Following *General Method D*, the reaction of a solution of 5-(benzyloxy)-1-(phenylsulfonyl)-1*H*-indole-2-carboxylic acid (**14**) (150 mg, 0.37 mmol) in dry DMF (1.5 mL) with DIPEA (0.2 mL, 1.29 mmol, 3.5 equiv), HATU (140 mg, 0.37 mmol), and NH_2_OH·HCl (26 mg, 0.37 mmol), after flash chromatography of the residue using DCM/methanol/NH_4_OH (90:9:1) as the eluent, gave compound **1** (10 mg, 6%) as a white solid: mp 108–110 °C; IR (cm^−1^) ν 3302 (N-H), 3031 (O-H), 1651 (C=O), 1377 (O=S=O), 1161 (C-O-C); ^1^H NMR (500 MHz, DMSO-d_6_) δ 8.13–8.09 (m, 2H), 7.87 (d, *J* = 9.1 Hz, 1H, H7), 7.73–7.66 (m, 1H), 7.63–7.55 (m, 2H), 7.47–7.35 (m, 4H), 7.34–7.27 (m, 1H), 7.22 (d, *J* = 2.5 Hz, 1H, H4), 7.08 (dd, *J* = 9.1, 2.5 Hz, 1H, H6), 6.90 (d, *J* = 0.8 Hz, 1H, H3), 5.09 (s, 2H) (signal for “N*H*” and “O*H*” were not detected); ^13^C NMR (126 MHz, DMSO-d_6_) δ 158.2, 155.3, 137.3, 137.0, 134.5 (CH_Ar_), 134.1, 130.3, 129.4 (2CH_Ar_), 129.3, 128.4 (2CH_Ar_), 127.9 (CH_Ar_), 127.7 (2CH_Ar_), 127.3 (2CH_Ar_), 115.7 (C6), 115.2 (C7), 112.5 (C3), 105.4 (C4), 69.6 (CH_2_); HRMS (ESI): Calcd for C_22_H_19_N_2_O_5_S^+^ [*M* + H]^+^: 423.1009. Found: 423.1016.

#### 3.1.10. 5-(Benzyloxy)-1-(phenylsulfonyl)-1H-indole-2-carbohydrazide (**2**)

Following *General Procedure E*, the reaction of a solution of ethyl 5-(benzyloxy)-1-(phenylsulfonyl)-1*H*-indole-2-carboxylate (**11**) (100 mg, 0.23 mmol) in dioxane (1.1 mL) with N_2_H_4_·H_2_O (0.06 mL, 1.15 mmol), after flash chromatography of the residue using DCM/methanol (1%) as the eluent, gave compound **2** (80 mg, 83%) as a brown solid: mp 101-3 °C; IR (cm^−1^) ν 3352 (N-H), 1679 (C=O), 1379 (O=S=O), 1169 (C-O-C); ^1^H NMR (400 MHz, CDCl_3_) δ 7.99–7.95 (m, 2H), 7.60–7.30 (m, 9H), 7.10 (dd, *J* = 9.1, 2.5 Hz, 1H, H6), 6.99 (d, *J* = 2.5 Hz, 1H, H4), 6.89 (s, 1H, H3), 5.04 (s, 2H) (signal for “N*H*” and “N*H_2_*” were not detected); ^13^C NMR (101 MHz, CDCl_3_) δ 162.9, 156.2, 137.0, 136.6, 134.1 (CH_Ar_), 133.9, 132.1, 129.7, 129.0 (2CH_Ar_), 128.6 (2CH_Ar_), 128.1 (CH_Ar_), 127.5 (2CH_Ar_), 127.4 (2CH_Ar_), 116.8 (C6), 116.4 (C7), 115.3 (C3), 105.3 (C4), 70.5 (CH_2_); HRMS (ESI): Calcd for C_22_H_20_N_3_O_4_S^+^ [*M* + H]^+^: 422.1169. Found: 422.1165.

#### 3.1.11. 1-(Phenylsulfonyl)-5-(3-(piperidin-1-yl)propoxy)-1H-indole-2-carbohydrazide (**3**)

Following *General Procedure E*, the reaction of a solution of ethyl 1-(phenylsulfonyl)-5-(3-(piperidin-1-yl)propoxy)-1H-indole-2-carboxylate (**12**) (100 mg, 0.21 mmol) in dioxane (1.1 mL) with N_2_H_4_·H_2_O (0.05 mL, 1.07 mmol), after flash chromatography of the residue using DCM/methanol (2%) as the eluent, afforded compound **3** (65 mg, 67%) as a white solid: mp 114-6 °C; IR (cm^−1^) ν 3211 (N-H), 1667 (C=O), 1376 (O=S=O), 1169 (C-O-C); ^1^H NMR (400 MHz, CDCl_3_) δ 7.98–7.96 (m, 2H), 7.61 (s, 1H, N*H*), 7.57–7.51 (m, 1H), 7.48–7.40 (m, 2H), 7.37–7.36 (m, 1H), 6.98 (dd, *J* = 9.1, 2.5 Hz, 1H, H6), 6.90 (d, *J* = 2.5 Hz, 1H, H4), 6.89 (d, *J* = 0.8 Hz, 1H, H3), 4.25–3.55 (m, 2H, N*H*_2_), 4.00 (t, *J* = 6.0 Hz, 2H), 2.93–2.65 (m, 6H), 2.19–2.08 (m, 2H), 1.81 (s, 4H), 1.60–1.50 (m, 2H); ^13^C NMR (101 MHz, CDCl_3_) δ 162.8, 156.0, 137.1, 134.1 (CH_Ar_), 132.0, 129.7, 129.0 (2CH_Ar_), 127.4 (2CH_Ar_), 125.9, 116.3 (C7), 116.3 (C6), 115.1 (C3), 104.8 (C4), 66.2 (CH_2_), 55.6 (CH_2_), 54.1 (2CH_2_), 25.4 (CH_2_), 24.3 (2CH_2_), 23.3 (CH_2_); HRMS (ESI): Calcd for C_23_H_29_N_4_O_4_S^+^ [*M* + H]^+^: 457.1904. Found: 457.1899.

#### 3.1.12. 1-(Phenylsulfonyl)-5-(3-(4-(prop-2-yn-1-yl)piperazin-1-yl)propoxy)-1H-indole-2-carbohydrazide (**4**)

Following *General Procedure E*, the reaction of a solution of ethyl 1-(phenylsulfonyl)-5-(3-(4-(prop-2-yn-1-yl)piperazin-1-yl)propoxy)-1*H*-indole-2-carboxylate (13) (150 mg, 0.29 mmol) in dioxane (1.5 mL) and N_2_H_4_·H_2_O (0.07 mL, 1.47 mmol), after flash chromatography of the residue using DCM/methanol (3%) as the eluent, provided compound **4** (39 mg, 31%) as a white solid: mp 122–124 °C; IR (cm^−1^) ν 3286 (N-H), 1664 (C=O), 1368 (O=S=O), 1169 (C-O-C); ^1^H NMR (400 MHz, CDCl_3_) δ 7.98–7.95 (m, 2H), 7.66 (s, 1H, N*H*), 7.58–7.50 (m, 1H), 7.48–7.39 (m, 2H), 6.99 (dd, *J* = 9.2, 2.5 Hz, 1H, H6), 6.91 (d, *J* = 2.5 Hz, 1H, H4), 6.89 (d, *J* = 0.8 Hz, 1H, H3), 4.46–3.55 (m, 2H, N*H*_2_), 4.00 (t, *J* = 6.1 Hz, 2H), 3.32 (d, *J* = 2.5 Hz, 2H), 2.83–2.50 (m, 10H), 2.28 (t, *J* = 2.5 Hz, 1H), 2.13–1.99 (m, 2H); ^13^C NMR (101 MHz, CDCl_3_) δ 162.8, 156.2, 137.1, 134.1 (CH_Ar_), 131.9, 129.7, 129.0, 129.0 (2CH_Ar_), 127.4 (2CH_Ar_), 116.4 (C7), 116.3 (C6), 115.1 (C3), 104.8 (C4), 78.4, 73.6 (CH), 66.3 (CH_2_), 55.0 (2CH_2_), 52.8 (2CH_2_), 50.9 (CH_2_), 46.6 (CH_2_), 29.7 (CH_2_); HRMS (ESI): Calcd for C_25_H_30_N_5_O_4_S^+^ [*M* + H]^+^: 496.2013. Found: 496.2016.

#### 3.1.13. N-(2-Aminophenyl)-5-(benzyloxy)-1-(phenylsulfonyl)-1H-indole-2-carboxamide (**5**)

Following *General Method D*, the reaction of a solution of 5-(benzyloxy)-1-(phenylsulfonyl)-1*H*-indole-2-carboxylic acid (**14**) (170 mg, 0.42 mmol) in dry DMF (1.7 mL) with DIPEA (0.2 mL, 1.04 mmol, 2.5 equiv), HATU (159 mg, 0.42 mmol), and 1,2-phenylenediamine (45 mg, 0.42 mmol), after flash chromatography of the residue using DCM/methanol (1%) as the eluent, afforded compound **5** (101 mg, 49%) as a brown solid: mp 129–131 °C; IR (cm^−1^) ν 3356 (N-H), 1663 (C=O), 1366 (O=S=O), 1151 (C-O-C); ^1^H NMR (400 MHz, DMSO-d_6_) δ 10.10 (s, 1H, N*H*), 8.10–8.08 (m, 2H), 7.90 (d, *J* = 9.1 Hz, 1H, H7), 7.72–7.68 (m, 1H), 7.61–7.57 (m, 2H), 7.45–7.32 (m, 5H), 7.29–7.21 (m, 2H), 7.17 (s, 1H, H3), 7.12–7.10 (m, 1H, H6), 6.99 (d, *J* = 7.6 Hz, 1H), 6.78 (d, *J* = 8.1 Hz, 1H), 6.61 (t, *J* = 7.6 Hz, 1H), 5.12 (s, 2H), 5.03 (s, 2H, N*H*_2_); ^13^C NMR (101 MHz, DMSO-d_6_) δ 159.7, 155.5, 143.1, 137.1, 137.0, 136.5, 134.6 (CH_Ar_), 130.3, 129.7 (CH_Ar_), 129.5 (2CH_Ar_), 128.4 (2CH_Ar_), 127.9 (CH_Ar_), 127.7 (2CH_Ar_), 127.2 (2CH_Ar_), 126.9, 126.4 (CH_Ar_), 122.1, 116.0 (CH_Ar_), 115.8 (CH_Ar_), 115.7 (C6), 115.5 (C7), 112.6 (C3), 105.6 (C4), 69.6 (CH_2_); HRMS (ESI): Calcd for C_28_H_24_N_3_O_4_S^+^ [*M* + H]^+^: 498.1482. Found: 498.1482.

#### 3.1.14. N-(2-Aminophenyl)-1-(phenylsulfonyl)-5-(3-(piperidin-1-yl)propoxy)-1H-indole-2-carboxamide (**6**)

Following *General Method D*, the reaction of a solution of 1-(phenylsulfonyl)-5-(3-(piperidin-1-yl)propoxy)-1H-indole-2-carboxylic acid (**15**) (200 mg, 0.45 mmol) in dry DMF (1.8 mL) with DIPEA (0.2 mL, 1.13 mmol, 2.5 equiv), HATU (172 mg, 0.45 mmol), and 1,2-phenylenediamine (49 mg, 0.45 mmol), after flash chromatography of the residue using DCM/methanol (2%) as the eluent, provided compound **6** (189 mg, 78%) as a brown solid: mp 123-5; IR (cm^−1^) ν 3335 (N-H), 1662 (C=O), 1377 (O=S=O), 1158 (C-O-C); ^1^H NMR (500 MHz, CDCl_3_) δ 8.03 (d, *J* = 9.1 Hz, 1H, H7), 7.92–7.86 (m, 2H), 7.80 (s, 1H, N*H*), 7.55–7.48 (m, 1H), 7.46 (dd, *J* = 8.3, 1.5 Hz, 1H), 7.42–7.34 (m, 2H), 7.16–7.09 (m, 2H), 7.03 (dd, *J* = 9.1, 2.5 Hz, 1H, H6), 6.93 (d, *J* = 2.5 Hz, 1H, H4), 6.89–6.82 (m, 2H), 4.17 (s, 2H, N*H*_2_), 4.02 (t, *J* = 6.2 Hz, 2H), 2.73–2.46 (m, 6H), 2.18–2.02 (m, 3H), 1.77–1.68 (m, 4H), 1.54–1.49 (m, 2H); ^13^C NMR (126 MHz, CDCl_3_) δ 160.0, 156.6, 141.5, 136.3, 136.0, 134.2 (CH_Ar_), 132.3, 130.4, 128.9 (2CH_Ar_), 127.7 (CH_Ar_), 127.5 (2CH_Ar_), 126.1 (CH_Ar_), 122.9, 119.1 (CH_Ar_), 117.5 (CH_Ar_), 117.4 (C3), 117.0 (C7), 116.5 (C6), 105.0 (C4), 66.6 (CH_2_), 55.8 (CH_2_), 54.4 (2CH_2_), 26.0 (CH_2_), 25.1 (2CH_2_), 23.8 (CH_2_); HRMS (ESI): Calcd for C_23_H_33_N_4_O_4_S^+^ [*M* + H]^+^: 533.2217. Found: 533.2212.

#### 3.1.15. N-(2-Aminophenil)-1-(phenylsulfonyl)-5-(3-(4-(prop-2-yn-1-yl)piperazin-1-yl)propoxy)-1H-indole-2-carboxamide (**7**)

Following *General Method D*, the reaction of a solution of 1-(phenylsulfonyl)-5-(3-(4-(prop-2-yn-1-yl)piperazin-1-yl)propoxy)-1*H*-indole-2-carboxylic acid (**16**) (180 mg, 0.37 mmol) in dry DMF (1.5 mL) with DIPEA (0.2 mL, 0.93 mmol, 2.5 equiv), HATU (142 mg, 0.37 mmol), and 1,2-phenylenediamine (40 mg, 0.37 mmol), after flash chromatography of the residue using DCM/methanol (4%) as the eluent, produced compound **7** (70 mg, 33%) as a yellow solid: mp 70-2 °C; IR (cm^−1^) ν 3287 (N-H), 1664 (C=O), 1367 (O=S=O), 1153 (C-O-C); ^1^H NMR (500 MHz, CDCl_3_) δ 8.03 (d, *J* = 9.1 Hz, 1H, H7), 7.93–7.83 (m, 2H), 7.76 (s, 1H, N*H*), 7.53–7.48 (m, 1H), 7.46 (dd, *J* = 8.5, 1.5 Hz, 1H), 7.38 (dd, *J* = 8.5, 7.6 Hz, 2H), 7.16–7.08 (m, 2H), 7.04 (dd, *J* = 9.1, 2.5 Hz, 1H, H6), 6.94 (d, *J* = 2.5 Hz, 1H, H4), 6.88–6.78 (m, 2H), 4.02 (t, *J* = 6.3 Hz, 2H), 3.31 (d, *J* = 2.5 Hz, 2H), 2.79–2.46 (m, 9H), 2.26 (t, *J* = 2.5 Hz, 1H), 2.06–1.93 (m, 2H) (signal for “N*H_2_*” was not detected); ^13^C NMR (126 MHz, CDCl_3_) δ 160.1, 156.8, 141.5, 136.3, 135.9, 134.2 (CH_Ar_), 132.3, 130.4, 128.9 (2CH_Ar_), 127.7 (CH_Ar_), 127.4 (2CH_Ar_), 126.1 (CH_Ar_), 122.9, 119.1 (CH_Ar_), 117.7 (C3), 117.4 (CH_Ar_), 117.1 (C7), 116.6 (C6), 105.0 (C4), 78.7, 73.3 (CH), 66.7 (CH_2_), 55.1 (CH_2_), 53.1 (2CH_2_), 51.8 (2CH_2_), 46.8 (CH_2_), 26.7 (CH_2_); HRMS (ESI): Calcd for C_31_H_34_N_5_O_4_S^+^ [*M* + H]^+^: 572.2326. Found: 572.2324.

### 3.2. Biological Assays—General Methods

#### 3.2.1. 5-HT_6_R Binding Assay

As described in ref. [26], 10 mM stock solutions of the tested compounds were prepared in DMSO. Serial dilutions of the compounds were prepared in 96-well microplates in assay buffer using the automated pipetting system, epMotion 5070 (Eppendorf, Hamburg, Germany). Each compound was tested at 8 concentrations from 10^−5^ to 10^−12^ M (final concentration). Radioligand binding was performed using membranes from CHO-K1 cells stably transfected with human 5-HT_6_R (PerkinElmer, Shelton, CT, USA). All assays were carried out in duplicate. Briefly, 50 µL of working solution of the tested compound, 50 µL of [^3^H]-LSD (final concentration 1.3 nM), and 150 µL of diluted membranes (2.5 µg protein per well) prepared in assay buffer (50 mM Tris, pH 7.4, 10 mM MgCl_2_, and 0.1 mM EDTA) were transferred to a 96-well polypropylene microplate using the 96-well pipetting station, Rainin Liquidator (MettlerToledo, Greifensee, Switzerland). Methiothepin (10 μM) was used to define nonspecific binding. The microplate was covered with sealing tape, mixed, and incubated for 60 min at 27 °C. The reaction was terminated by rapid filtration through a GF/A filter presoaked with 0.5% polyethyleneimine for 30 min. Ten rapid washes with 200 µL of 50 mM Tris buffer (4 °C, pH 7.4) were performed using the automated harvester system Harvester-96 MACH III FM (Tomtec, Unterschleißheim, Germany). The filters were dried at 37 °C in a forced-air fan incubator, and then solid scintillator MeltiLex was melted on the filters at 90 °C for 5 min. Radioactivity was counted using a MicroBeta2 scintillation counter (PerkinElmer, Shelton, CT, USA). Data were fitted to a one-site curve-fitting equation using Prism 6 (GraphPad Software, San Diego, CA, USA), and Ki values were estimated using the Cheng−Prusoff equation [40].

#### 3.2.2. In Vitro Inhibitory Activity Toward Human Recombinant AChE and Human Serum BuChE

Ellman’s spectrophotometric assay [27], with small modifications, was applied to test the inhibitory activities of the synthesized compounds against human cholinesterases. The reagents used to perform the experiments were purchased from Sigma-Aldrich (Steinheim, Germany); only human butyrylcholinesterase (*h*BuChE) isolated from human plasma was from Vivonics (Bedford, MA, USA). The protocol described below was followed. Briefly, 5 U/mL aqueous stock solutions of the enzymes (*h*AChE and *h*BuChE) were diluted before use to a final concentration of 0.384 U/mL. Stock solutions of the tested compounds were prepared in DMSO and diluted (in demineralized water) to the desired concentration prior to use. First, 25 μL of the target compound (or water or mixture of DMSO/water at an appropriate ratio; i.e., blank samples) was added to 200 μL of 0.1 M phosphate buffer (pH = 8.0) and incubated for 5 min in 36 °C with DTNB (20 μL; 0.0025M) and the enzyme (20 μL; *h*AChE or *h*BuChE). The final reaction was initiated by adding 20 μL of ATC (0.00375M) or BTC (0.00375M) solution (depending on the enzyme used). After 5 min, the change in absorbance was measured at 412 nm using a microplate reader (EnSpire Multimode; PerkinElmer, Waltham, MA, USA). The tested compounds were tested at a screening concentration of 10 µM. Based on the equation 100 − (S/B) × 100 (where S and B are the respective enzyme activities with and without the test sample, respectively), the percentage of inhibition of each enzyme by the tested compounds was calculated. Owing to the enzyme inhibitory activities at 10 μM being better than 30%, the tested compounds were further evaluated to obtain their IC_50_ values. The IC_50_ value was determined based on the inhibited enzyme activity at six different concentrations of the tested compound, resulting in an inhibition between 5% and 95%. Calculations were performed using nonlinear regression (GraphPad Prism 9; GraphPad Software, San Diego, CA, USA) by plotting the residual enzyme activity against the applied inhibitor concentration. Tacrine was used as the reference compound. All experiments were performed in triplicate.

#### 3.2.3. In Vitro HDAC Inhibition Assay

In vitro inhibitory activity against HDAC1 and HDAC6 was determined using a modified protocol based on our previously published assay [28]. For compounds and controls, 3-fold serial dilutions of the respective DMSO-stock solution (DMSO: Carl Roth GmbH, Karlsruhe, Germany) in assay buffer [50 mM Tris−HCl, pH 8.0, 137 mM NaCl, 2.7 mM KCl, 1.0 mM MgCl_2_•6xH_2_O, 0.1 mg/mL BSA (all components: Sigma-Aldrich/Merck KGaA, Darmstadt, Germany)], were prepared and 5.0 µL of these serial dilutions were transferred into OptiPlate-96 black microplates (PerkinElmer, Waltham, MA, USA). Then, 25 µL of assay buffer and 10 µL of enzyme solution (human recombinant HDAC1 [BPS Bioscience (San Diego, CA, USA), Catalog# 50051]; HDAC6 [BPS Bioscience (San Diego, CA, USA), Catalog# 50006)] were added. In the case of *o*-aminoanilide and hydrazide-based inhibitors, which are known for their slow binding characteristics [41,42], inhibitor and enzyme (HDAC1) were preincubated at 25 °C for 60 min. Afterward, the fluorogenic substrate ZMAL (Z-Lys(Ac)-AMC [28]; 10 µL; 75 µM in assay buffer) was added. The total assay volume (50 µL, max. 1% DMSO) was incubated at 37 °C for 90 min. Subsequently, 50 µL of trypsin solution (0.4 mg/mL trypsin (Sigma-Aldrich/Merck KGaA, Darmstadt, Germany) in buffer: 50 mM Tris−HCl, pH 8.0, 100 mM NaCl (all components: Sigma-Aldrich/Merck KGaA, Darmstadt, Germany) was added, followed by an additional 30 min of incubation at 37 °C. Fluorescence (excitation: 355 nm, emission: 460 nm) was measured using a FLUOstar OPTIMA microplate reader (BMG LABTECH, Ortenberg, Germany). IC_50_ values were determined by generating normalized dose–response curves using the built-in “log(inhibitor) vs. response (three parameters)” equation provided by GraphPad Prism (GraphPad Prism 9.0, San Diego, CA, USA). All compounds were tested in duplicate, reported mean IC_50_ values, including the standard deviation, were calculated from at least two independent experiments.

### 3.3. Parallel Artificial Membrane Permeation Assay—Blood–Brain Barrier (PAMPA-BBB)

To evaluate the brain–barrier penetration ability of the different compounds, a parallel artificial membrane permeation assay for the blood–brain barrier was used, following the method described by Di et al. [29]. The in vitro permeability (P_e_) of 14 commercial drugs through a lipid extract of porcine brain membranes, together with that of the test compounds, was determined. Commercial drugs and assayed compounds were tested using a mixture of PBS and EtOH (70:30). Assay validation was performed by comparing the experimental permeability with the reported values of the commercial drugs in the literature, and the linear correlation between the experimental and reported permeabilities of the 14 commercial drugs using the parallel artificial membrane permeation assay was evaluated (y = 1590 × − 1161; R^2^ = 0.9419). From this equation and taking into account the limits established by Di et al. for BBB permeation, we established the ranges of permeability as compounds of high BBB permeation (CNS+): *P*e (10^−6^ cm s^−1^) > 5201; compounds of low BBB permeation (CNS−): *P*e (10^−6^ cm s^−1^) < 2020; and compounds of uncertain BBB permeation (CNS+/−):5201 *P*e (10^−6^ cm s^−1^) > 2020. Table 2 shows the permeability results from the different commercial and assayed compounds (three different experiments in triplicate) and predicted penetration in the CNS.

### 3.4. Worm Strains, Maintenance, and General Methods

Standard procedures were followed for culturing *C. elegans*, unless otherwise noted. The strains used in this study and their abbreviations are listed in Table 6. The N2 (Bristol) wild-type strain was used as the control in the experiments, except for the experiments with HD strains, in which EAK102 was used, as it presents the same genetic background as the disease model (EAK103). All of the strains used in this study were grown in a temperature-controlled incubator at 16 °C on solid nematode growth medium (NGM) seeded with Escherichia coli (*E. coli*) OP50 as a food source. The bleaching technique was used to obtain an age-synchronized egg population by treating gravid adults with a bleach-containing alkaline hypochlorite solution (0.5 M NaOH, ∼2.6% NaClO) for 5 to 7 min. Fertilized eggs were spun down and resuspended in S-medium for 12 h. In this way, the first larval stage (L1) was able to grow in the absence of nutrients overnight.

#### 3.4.1. Compound Preparation and Treatment

MTP150 was serially diluted to a range between 10 mM and 0.0001 mM with 100% dimethyl sulfoxide (DMSO, Sigma Aldrich, St. Louis, MO, USA). Each concentration was then diluted with MilliQ purified water to obtain a final concentration ranging between 100 µM and 0.001 µM in 1% DMSO per well. The treatment was performed in liquid culture for 4 days at 20 °C. Each well contained a final volume of 60 µL, comprising 25–35 animals in the L1 stage, the compound under study at the appropriate dose, and OP50 inactivated by freeze–thaw cycles and suspended in S-medium complete to a final optical density = 595 nm (OD595) of 0.9–0.8, as measured in the microplate reader (Benchmark™ Plus Microplate Reader, BioRad, Hercules, CA, USA).

#### 3.4.2. Locomotor Activity Assay

Worms were treated as described above in liquid culture for four days, starting at the L1 stage. At day 4, the 96-well microplate was read using the Wmicrotracker^®^ MINI (PhylumTech S. A., Santa Fe, Argentina). Activity counts measured for 20 min were obtained using WMicrotracker MINI Software V1.4 API (PhylumTech S. A., Santa Fe, Argentina).

#### 3.4.3. Statistical Analysis

The data were analyzed using GraphPad Prism Version 9 software (GraphPad Software, San Diego, CA, USA). Groups were compared via one-way analysis of variance (ANOVA), followed by Dunnett’s post hoc test. Results are expressed as the mean ± standard error of the mean (SEM) of n = 8. Statistical significance was assumed when *p* values were <0.05. Statistical outliers were identified using Grubbs’ test and removed from the analysis.

### 3.5. Drosophila and Human Cell Models of PD: Materials and Method

#### 3.5.1. Drosophila Stocks

*DJ-1β* mutant flies from the *DJ-1β^ex54^* strain were used in this study. Flies were maintained at 25 °C and cultured in *Drosophila* standard medium.

#### 3.5.2. Drug Treatments and Climbing Assays in PD Model Flies

To evaluate the effect of MTP150 on the locomotor ability of *DJ-1β* mutant flies, L2 larvae were collected and cultured in standard food containing 0.1% DMSO as vehicle or supplemented with the tested compound at final concentrations of 1 µM and 10 µM. After eclosion, adult female flies were transferred to new tubes, and climbing assays were performed five days later [37]. The experiments were carried out three times.

#### 3.5.3. Quantification of Protein Carbonylation Levels in Fly Extracts

Protein carbonyl groups were quantified in extracts obtained from 5-day-old *DJ-1β* mutant flies treated with MTP150 at 1 µM and 10 μM doses, or 0.1% DMSO, using 2,4-dinitrophenyl hydrazine derivatization [3]. All experiments were carried out using three biological replicates and three technical replicates for each sample.

#### 3.5.4. Quantification of ATP Levels in Fly Extracts

ATP levels were measured in extracts from 5-day-old *DJ-1β* mutants treated with MTP150 at 1 µM and 10 μM doses, or 0.1% DMSO, as previously described [3] using the ATP Determination Kit (Invitrogen, OR, USA), following the manufacturer’s instructions. All experiments were performed in triplicate.

#### 3.5.5. SH-SY5Y Cell Culture and MTP150 Dosage Assays

The pLKO.1 control and *DJ-1*-deficient SH-SY5Y neuron-like cells used in this study were cultured at 37 °C and 5% CO_2_ in selective growth medium consisting of Dulbecco’s modified Eagle’s medium/Nutrient Mixture F-12 (DMEM/F12) (Biowest, Nuaillé, France) and supplemented with 10% (*v*/*v*) fetal bovine serum (Capricorn, Ebsdorfergrund, Germany), 1% non-essential amino acids, 100 mg/mL penicillin/streptomycin (Biowest, Nuaillé, France), and 2 μg/mL puromycin (ApexBio, Hsinchu, Taiwan). Cell viability after supplementation with different concentrations of MTP150 (either in the presence or in the absence of 100 µM H_2_O_2_) was evaluated using the MTT (3-(4, 5-dimethylthiazol-2-yl)-2-5-diphenyltetrazolium bromide) assay, as previously described [37]. All experiments were carried out using three biological replicates and three technical replicates for each sample.

## 4. Conclusions

In this study, we have described the most recent progress on our current project aimed at identifying new MSMs for the treatment of NDs. In the present case, we have concentrated our efforts on the design, synthesis, and biological evaluation of polyfunctionalized *N*-arylsulfonyl indole derivatives designed by juxtaposing the *N*-benzenesulfonyl motif present in 5-HT_6_R antagonists with selected pharmacophore groups present in reference ligands such as Contilisant, Tubastatin A, and Belinostat to act as 5-HT_6_R multitarget small molecules. This effort resulted in several hybrids of types **I**, **II** (Figure 1 and Figure 2), and **III** (Figure 3). The in vitro biological evaluation of selected targets (ChEs, MAOs, HDAC1/6, and 5-HT_6_R), plus the analysis of BBB permeability, allowed us to choose compound MTP150 (Figure 2) for in vivo analysis in suitable ND models. MTP150 reduced protein aggregation, modulated matrix metalloproteinase activity, mitigated neuroinflammation, and enhanced DNA damage repair pathways in *C. elegans* in vivo models of AD, PD, and HD. Furthermore, results showed that hybrid MTP150 enhanced CNS delivery through BBB penetration, leading to disease progression stabilization in AD, PD, and HD models via reduction of oxidative stress and neuroinflammation. We also demonstrated that MTP150 alleviated PD-related phenotypes in both *Drosophila* and human cell PD models based on *DJ-1* inactivation. In PD model flies, MTP150 administration was able to improve motor performance, reduce oxidative stress levels, and restore mitochondrial function (reflected by an increase of ATP levels). Moreover, MTP150 displayed a marked neuroprotective effect by increasing the viability of *DJ-1-*deficient SH-SY5Y human cells cultured under oxidative stress conditions. Collectively, these results highlight the disease-modifying properties of MTP150 and support its potential as a therapeutic candidate for treating PD.

## Figures and Tables

**Table 1:** Results of the affinity of Contilisant+Tubastatin A hybrids of type **I** for 5-HT_6_R ^a^.

Compound	5-HT_6_R Ki ± SD (nM) ^a^
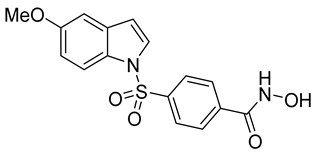 MTP89	6305 ± 1246
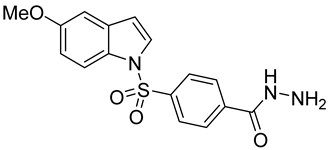 MTP86	6770 ± 1471
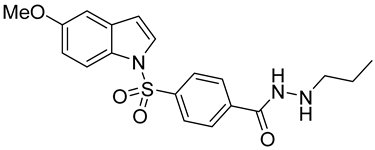 FRB24 [3]	5820 ± 839
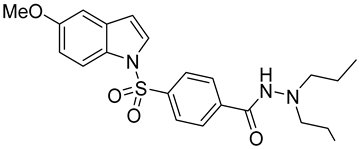 FRB21	19370 ± 3986
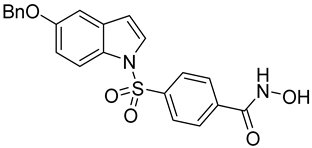 MTP98	1543 ± 272
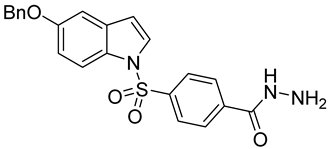 MTP90	31510 ± 4981
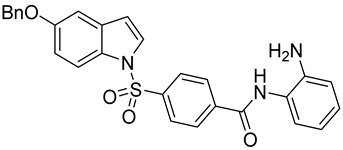 MTP155	57960 ± 13135
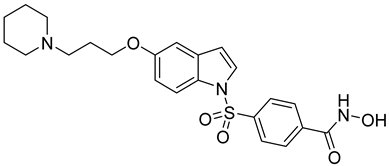 MTP100 [3]	3392 ± 574
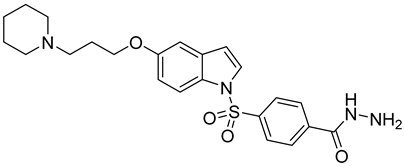 MTP96	17360 ± 2364
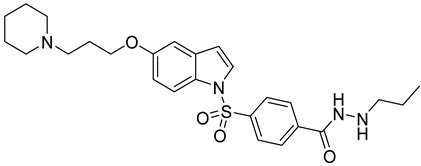 FRB44 [3]	4307 ± 703
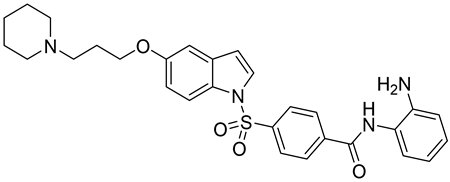 MTP165 [3]	1462 ± 135
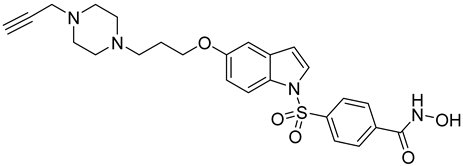 MTP109 [3]	6376 ± 1198
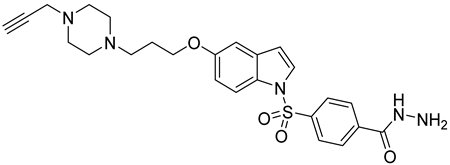 MTP99	16220 ± 2892
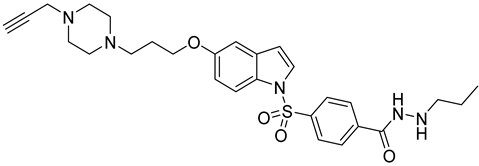 FRB56 [3]	30650 ± 6761
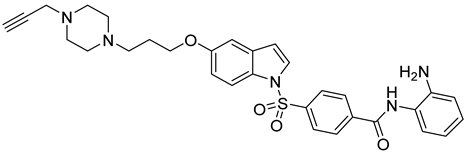 MTP195 [3]	4403 ± 917

^a^ Ki values expressed as the mean value ± SD of two technical replicates.

**Table 2:** Results of the affinity of Contilisant+Belinostat hybrids of type II for 5-HT_6_R ^a^.

Compound	5-HT_6_R Ki ± SD (nM) ^a^
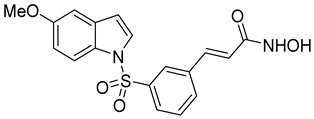 MTP142	38610 ± 9134
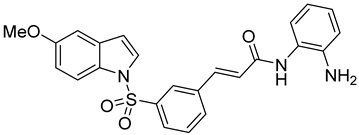 MTP143	2243 ± 361
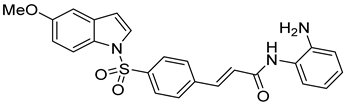 SMD10	34320 ± 5324
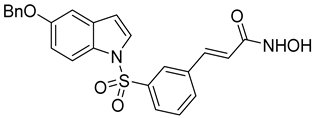 MTP156	6122 ± 891
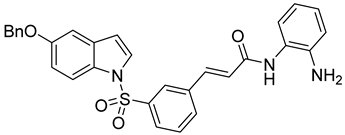 MTP157	8771 ± 1697
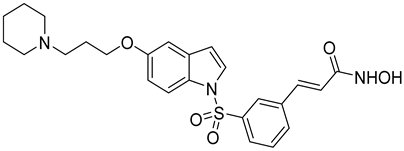 MTP150	13580 ± 2697
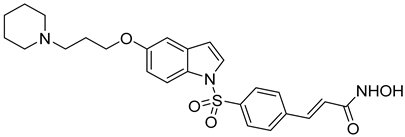 APP19	8670 ± 1921
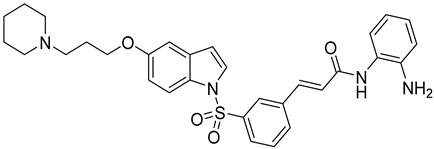 MTP167	6981 ± 983
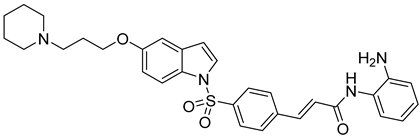 APP17	3576 ± 438
Belinostat	1034 ± 98

^a^ Ki values expressed as the mean value ± SD of two technical replicates.

**Table 3:** ChEs inhibition by compound **6**.

Compound	*h*AChE % inh. (10 µM) ± SD ^a^	*h*AChE IC_50_ [μM] ± SEM ^b^	*h*BuChE % inh. (10 µM) ± SD ^a^	*h*BuChE IC_50_ [μM] ± SEM ^c^
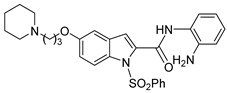 **6**	33.6 ± 1.7	26.78 ± 0.59	91.4 ± 0.1	0.97 ± 0.04
Tacrine	97.1 ± 0.2	0.19 ± 0.01	99.3 ± 0.1	0.03 ± 0.001

^a^ Mean value ± standard deviation (SD) of three independent experiments; ^b^ half maximal inhibitory concentration of the tested compound for human recombinant AChE; mean value ± standard error of the mean (SEM) of triplicates; ^c^ half maximal inhibitory concentration of the tested compound for BuChE from human serum; mean value ± standard error of the mean (SEM) of triplicates.

**Table 4:** Permeability (*Pe* 10^−6^ cm s^−1^) in the PAMPA-BBB assay of selected Contilisant+Tubastatin A hybrids of type **I** and Vorinostat, showing the predicted penetration in the CNS.

Compounds	Biological Targets	Experimental Value (n = 3) ± S.D.	CNS Prediction
MTP89	HDAC1 (3.39 μM); HDAC6 (0.17 μM); 5-HT_6_R (6.305 μM)	23.3 ± 4.2	CNS+
FRB24	HDAC1 (0.087 μM); 5-HT_6_R (5.820 μM)	14.5 ± 1.0	CNS+
MTP98	HDAC1 (3.99 μM); HDAC6 (0.295 μM); 5-HT_6_R (1.543 μM)	7.5 ± 0.25	CNS+
MTP165	HDAC1 (0.24 μM); *eq*BuChE (0.263 μM); *h*H_3_R (48%); 5-HT_6_R (1.462 μM)	6.65 ± 0.3	CNS+
MTP155	HDAC1 (0.42 μM); 5-HT_6_R (57.96 μM)	6.3 ± 0.3	CNS+
MTP86	5-HT_6_R (6.77 μM)	4.2 ± 0.4	CNS+/−
FRB44	HDAC1 (0.11 μM); *eq*BuChE (1.18 μM); 5-HT_6_R (4.30 μM)	3.5 ± 0.35	CNS+/−
MTP109	HDAC1 (4.00 μM); HDAC6 (0.035 μM); 5-HT_6_R (6.37 μM)	2.3 ± 0.5	CNS+/−
MTP195	HDAC1 (0.14 μM); *eq*BuChE (0.89 μM); *h*MAO B (0.56 μM); 5-HT_6_R (4.40 μM)	2.3 ± 0.1	CNS+/−
MTP100 ^a^	HDAC1 (0.74 μM); HDAC6 (0.012 μM); *h*H_3_R (43%); 5-HT_6_R (3.39 μM)	1.6 ± 0.3	CNS−
Vorinostat	HDAC1 (0.089 μM); HDAC6 (0.029 μM)	1.8 ± 0.25	CNS−

^a^ MW: 457.55. Poorly dissolved, turbid at 50% in DMSO.

**Table 5:** Permeability (*Pe* 10^−6^ cm s^−1^) in the PAMPA-BBB assay of selected Contilisant+Belinostat hybrids II, and Vorinostat showing the predictive penetration in the CNS.

Compounds	Biological Targets	Experimental Value (n = 3) ± S.D.	CNS Prediction
MTP156	HDAC1 (0.22 μM); HDAC6 (0.34 μM); MAO A (15.0 μM); 5-HT_6_R (6.122 μM)	7.1 ± 0.15	CNS+
MTP150	HDAC1 (0.019 μM); HDAC6 (0.04 μM); AChE (19.1 μM); BuChE (27.1 μM); MAO A (8.6 μM); 5-HT_6_R (13.580 μM)	6.5 ± 0.7	CNS+
SMD10	HDAC1 (1.24 μM); 5-HT_6_R (34.320 μM)	3.7 ± 0.3	CNS+/−
MTP142	HDAC1 (0.065 μM); HDAC6 (0.112 μM); MAO A (11.9 μM); 5-HT_6_R (38.61 μM)	3.5 ± 0.3	CNS+/−
MTP167	AChE (21.6 μM); BuChE (3.48 μM); 5-HT_6_R (6.98 μM)	3.35 ± 0.2	CNS+/−
APP17	HDAC1 (2.32 μM); AChE (12.4 μM); BuChE (5.48 μM); 5-HT_6_R (3.58 μM)	2.1 ± 0.1	CNS+/−
APP19	HDAC1 (0.126 μM); HDAC6 (0.020 μM); MAO A (8.29 μM); 5-HT_6_R (8.67 μM)	0.7 ± 0.1	CNS−
Vorinostat	HDAC1 (0.089 μM); HDAC6 (0.029 μM)	1.8 ± 0.25	CNS−

**Table 6:** List of *C. elegans* strains used in this study.

Strain	Genotype	Source
N2 (Bristol)	*C. elegans* wild-type	CGC
CL2006	dvIs2 (pCL12 (unc-54/human Abeta peptide 1–42 minigene) + rol-6(su1006))	CGC
BR3579	ced-10(n3246)	Esther Dalfó lab
EAK102	eeeIs1 [unc-54p::Htt513(Q15)::YFP::unc-45 3′UTR]	CGC
EAK103	eeeIs2 [unc-54p::Htt513(Q128)::YFP::unc-45 3′UTR]	CGC

## Data Availability

The original contributions presented in the study are included in the article/Supplementary Materials, further inquiries can be directed to the corresponding authors.

## Supplementary Materials

The following supporting information can be downloaded at: https://www.mdpi.com/article/10.3390/ijms27073135/s1. References [43,44,45,46,47] are cited in the Supplementary Materials.
