# Supplementary material for: Polyfunctionalized N-Arylsulfonyl Indoles: Identification of (E)-N-Hydroxy-3-{3-[(5-(3-(piperidin-1-yl)propoxy]-1H-indol-1-yl)sulfonyl]phenyl}acrylamide (MTP150) for the Epigenetic-Based Therapy of Parkinson’s Disease
Source: Int J Mol Sci. 2026 Mar 30;27(7):3135. doi: 10.3390/ijms27073135 (PMC13073873; doi:10.3390/ijms27073135)
Supplement: Supplementary file 1 [file ijms-27-03135-s001.zip › ijms-4155078-supplementary.pdf]

Supplementary Materials

Polyfunctionalized *N*-Arylsulfonyl indoles: Identification of (*E*)-*N*-Hydroxy-3-{3-[(5-(3-(piperidin-1-yl)propoxy]-1*H*-indol-1-yl)sulfonyl]phenyl}acrylamide (MTP150) for the Epigenetic-based Therapy of Parkinson's Disease

Mireia Toledano-Pinedo,[a] Alicia Porro-Pérez,[†,a] Linda Schäker-Hübner,[†,b] Daniel Diez-Iriepa,[a] Isabel Iriepa,[c,d] Agata Siwek,[e] Małgorzata Wolak,[e] Grzegorz Satała, [f]Andrzej J. Bojarski,[f] Agata Doroz-Płonka,[g] Jadwiga Handzlik,[g] Justyna Godyń,[h] Patrick Dallemagne,[i] Christophe Rochais,[i] Audrey Davis,[j] Marc Since,[j] Belén Pérez,[k] Aina Bellver-Sanchis,[l] Alba Irisarri,[l] Mercè Pallàs,[l,m,n] Cristina Solana-Manrique, [o,p,] Francisco López-Muñoz,[q,r,s] Lhassane Ismaili,[t] Christian Griñán-Ferré,[l,m,n] Nuria Paricio,[o,p,*] Finn K. Hansen,[b] Anna Wieckowska,[h] and José Marco-Contelles[a,*]

[a] Institute of General Organic Chemistry (CSIC), C/ Juan de la Cierva 3, 28006-Madrid, Spain
[b] Pharmaceutical Institute, University of Bonn, An der Immenburg 4, 53121 Bonn, Germany
[c] Universidad de Alcalá, Departamento de Química Orgánica y Química Inorgánica, Instituto de Investigación Química "Andrés M. del Río" (IQAR), 28805-Alcalá de Henares, Madrid, Spain
[d] Grupo DISCOBAC, Instituto de Investigación Sanitaria de Castilla-La Mancha (IDISCAM), Spain
[e] Department of Pharmacobiology, Faculty of Pharmacy, Jagiellonian University Medical College, 9 Medyczna St., 30-688 Krakow, Poland
[f] Maj Institute of Pharmacology Polish Academy of Sciences, 12 Smętna St., 31-343 Kraków, Poland
[g] Department of Technology and Biotechnology of Drugs, Medical College, Jagiellonian University, 9 Medyczna St., 30-688 Krakow, Poland
[h] Department of Physicochemical Drug Analysis, Faculty of Pharmacy, Jagiellonian University Medical College, 9 Medyczna St., 30-688 Krakow, Poland
[i] Université de Caen Normandie, Normandie Univ, CERMN UR4258, F-14000 Caen, France
[j] Université de Caen Normandie, Normandie Univ, CERMN UR4258, Druid Platform, F-14000 Caen, France
[k] Department of Pharmacology, Therapeutic and Toxicology. Universitat Autònoma de Barcelona, E-08193 Barcelona, Spain
[l] Pharmacology Section, Department of Pharmacology, Toxicology and Therapeutic Chemistry, Faculty of Pharmacy and Food Sciences, Institute of Neuroscience, Universitat de Barcelona (NeuroUB), Av. Joan XXIII 27–31, 08028 Barcelona, Spain
[m] Institut de Neurociències, Universitat de Barcelona (NeuroUB), Barcelona, Spain
[n] Spanish Biomedical Research Center inNeurodegenerative Diseases (CIBERNED)-Instituto de Salud Carlos III, Madrid, Spain
[o] Departamento de Genética, Facultad CC Biológicas, Universidad de Valencia, 46100 Burjassot, Spain
[p] Instituto Universitario de Biotecnología y Biomedicina (BIOTECMED), Universidad de Valencia, 46100 Burjassot, Spain
[q] Faculty of Health Sciences–HM Hospitals, Camilo José Cela University, 28692 Madrid, Spain
[r] HM Hospitals Health Research Institute, 28015 Madrid, Spain
[s] Neuropsychopharmacology Unit, "Hospital 12 de Octubre" Research Institute, Madrid, Spain
[t] Université de Franche-Comté, INSERM, UMR 1322 LINC, F-25000 Besançon, France

CONTENT

1. **NMR spectra and HPLC/MS of compounds 1-7……………………S2-S15**
2. **Molecular docking of compound 2 into the *h*AChE, *h*BuChE, *h*HDAC1, *h*HDAC6 and *h*5-HT6R………………………………...S16-S18**
3. **Molecular docking of compound 6 in *h*BuChE…..…………………S19**
4. **Molecular modelling of MTP150 on HDAC1,6…………………….S20-S23**
5. **ADME of MTP150…………………………………………………S24-S26**
6. **References………………………………………………………..S27**

1. NMR spectra and HPLC/MS of compounds 1-7

5-(Benzyloxy)-*N*-hydroxy-1-(phenylsulfonyl)-1*H*-indole-2-carboxamide (1)

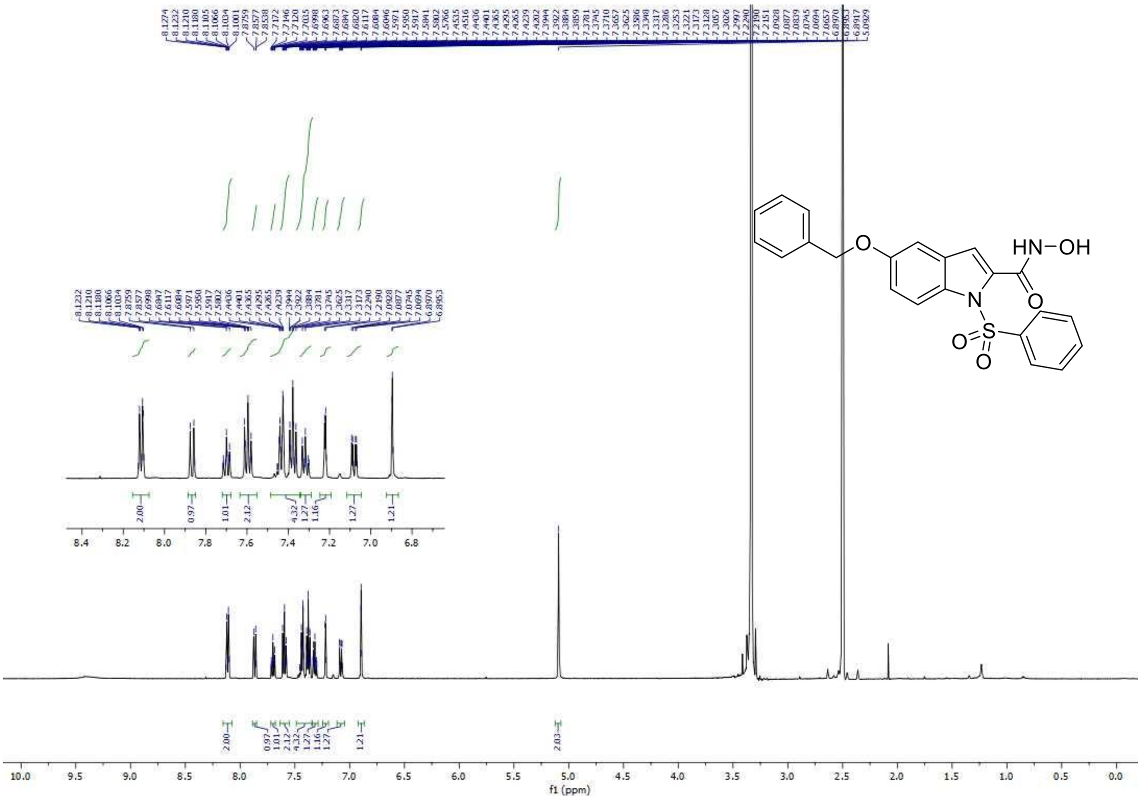

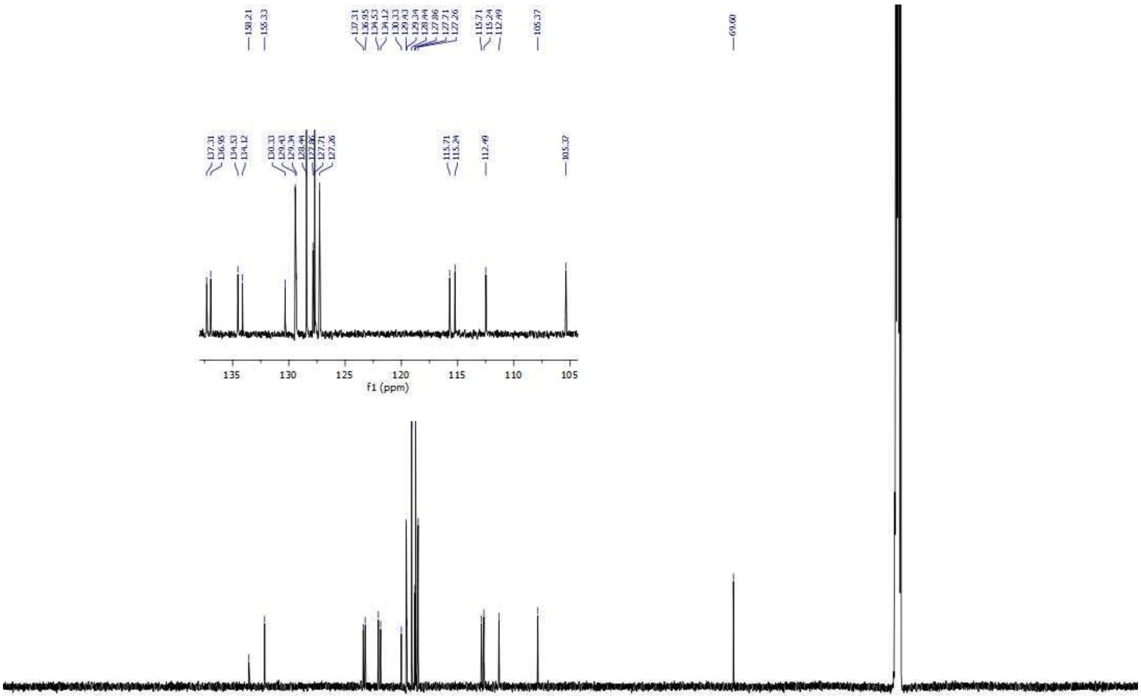

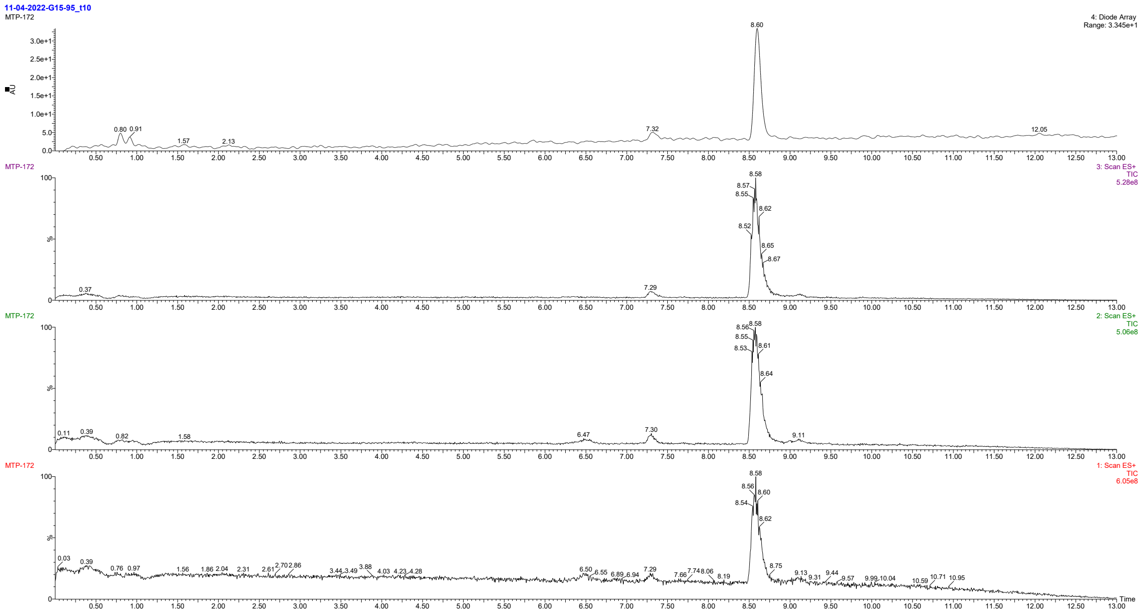

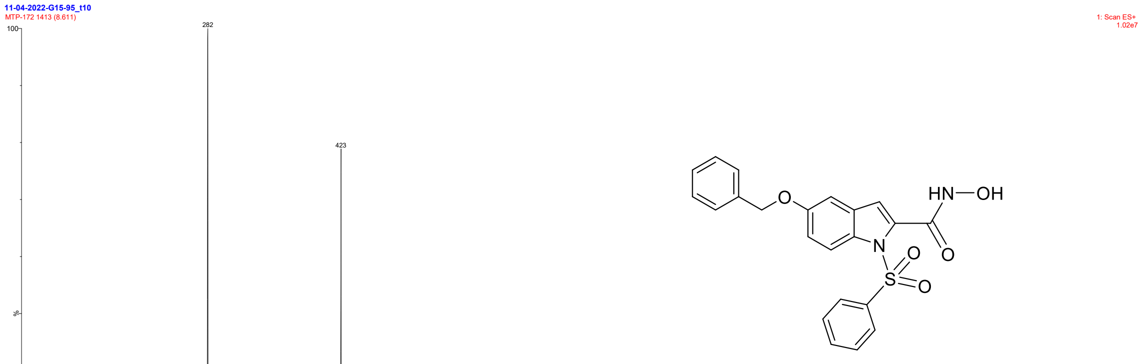

Chemical Formula: $C_{22}H_{18}N_2O_5S$
Molecular Weight: 422,4550

5-(Benzyloxy)-1-(phenylsulfonyl)-1*H*-indole-2-carbohydrazide (2)

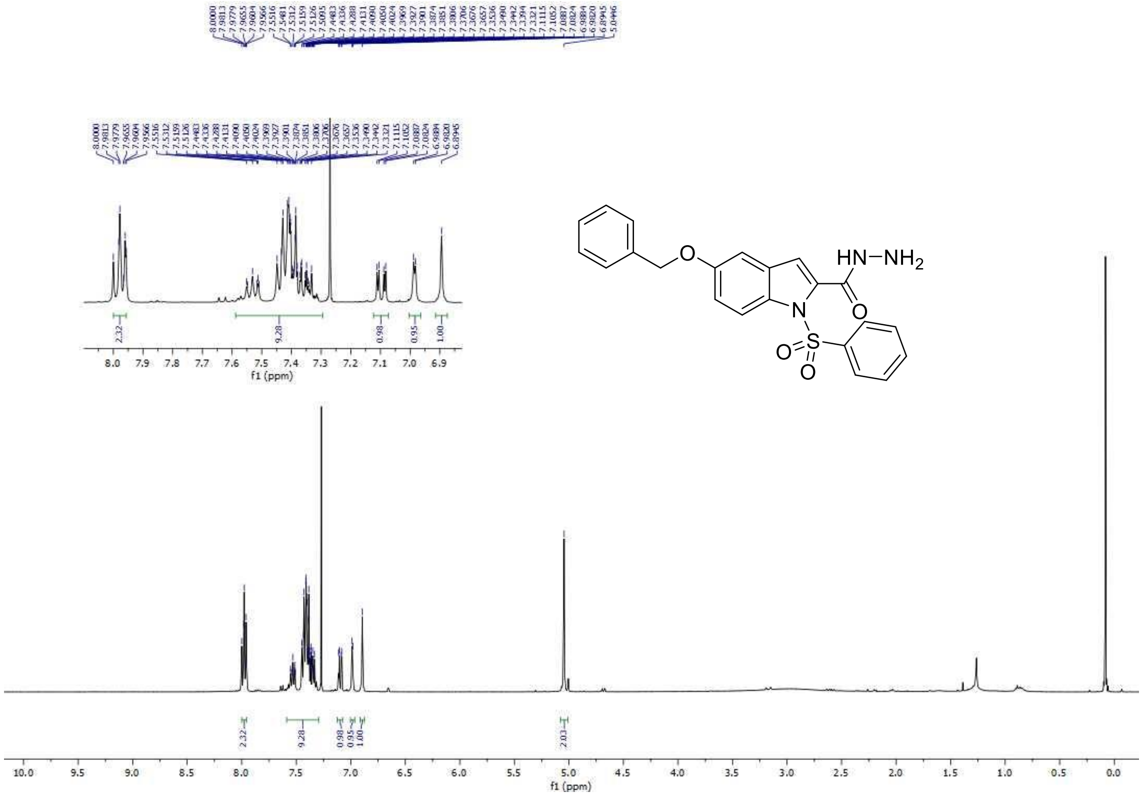

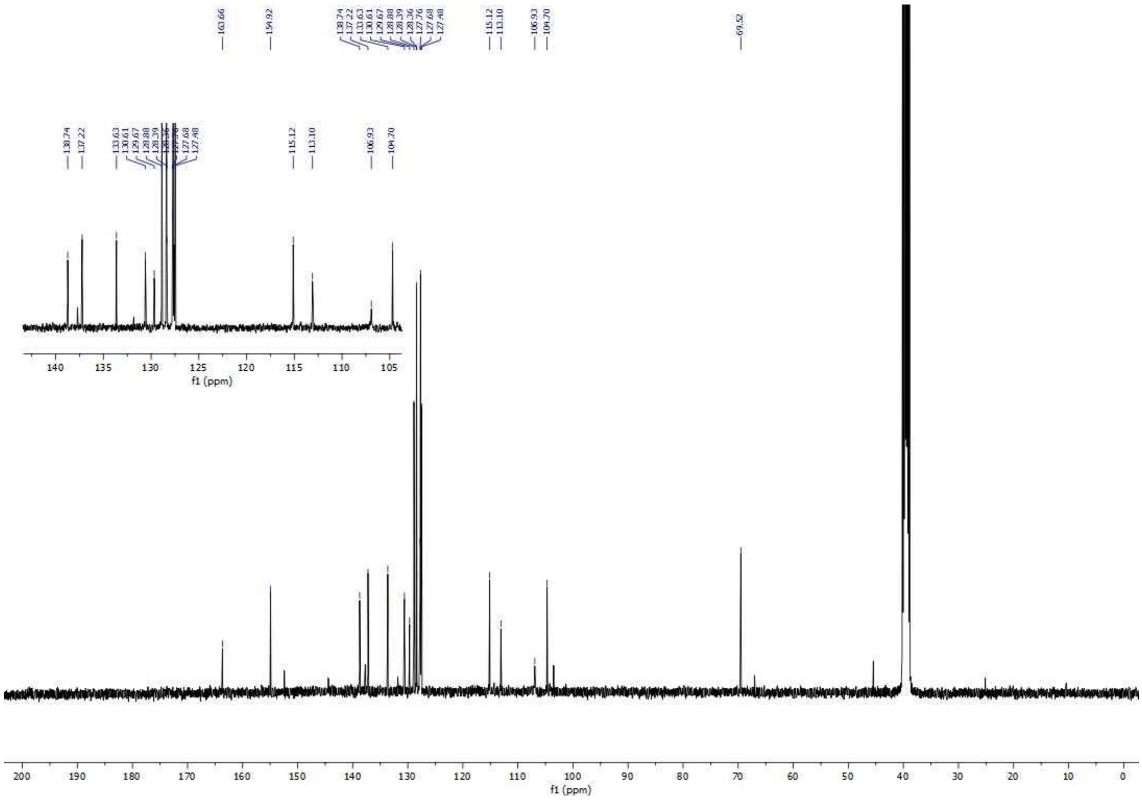

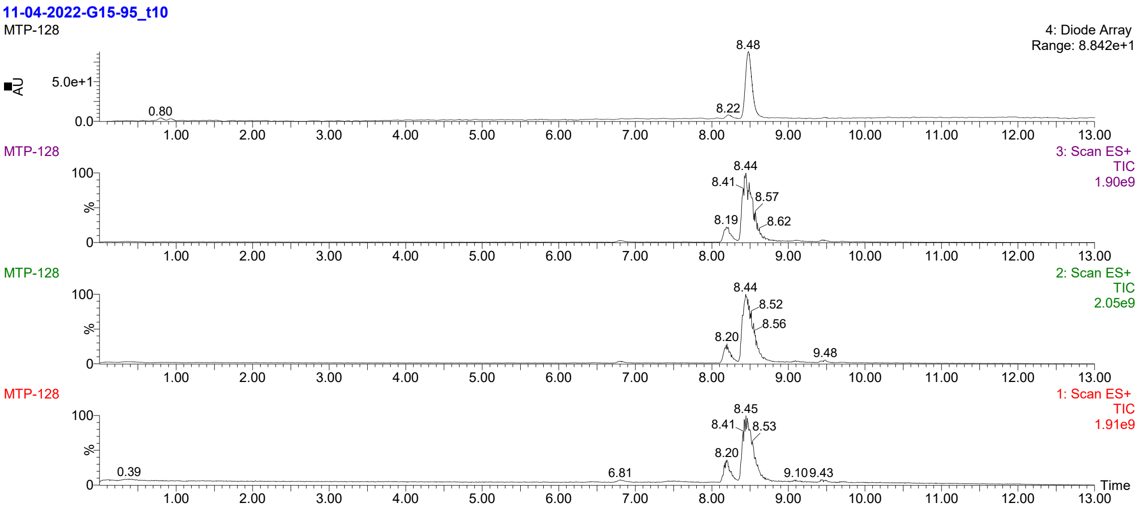
11-04-2022-G15-95_t10
MTP-128
4: Diode Array
Range: 8.842e+1
AU
5.0e+1
0.0
0.80
8.22
8.48
MTP-128
3: Scan ES+
TIC
1.90e9
8.19
8.41
8.44
8.57
8.62
MTP-128
2: Scan ES+
TIC
2.05e9
8.20
8.44
8.52
8.56
9.48
MTP-128
1: Scan ES+
TIC
1.91e9
0.39
6.81
8.20
8.41
8.45
8.53
9.10
9.43
Time

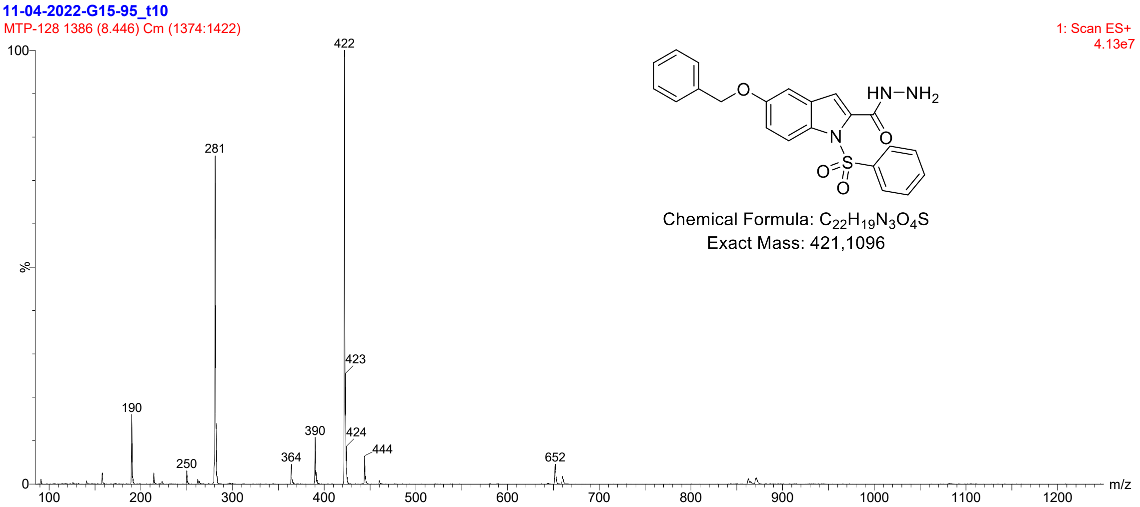
11-04-2022-G15-95_t10
MTP-128 1386 (8.446) Cm (1374:1422)
1: Scan ES+
4.13e7
%
190
250
281
364
390
422
423
424
444
652
m/z
HN−NH2
O
N
O=S
O
Chemical Formula: C22H19N3O4S
Exact Mass: 421,1096

1-(Phenylsulfonyl)-5-(3-(piperidin-1-yl)propoxy)-1*H*-indole-2-carbohydrazide (3)

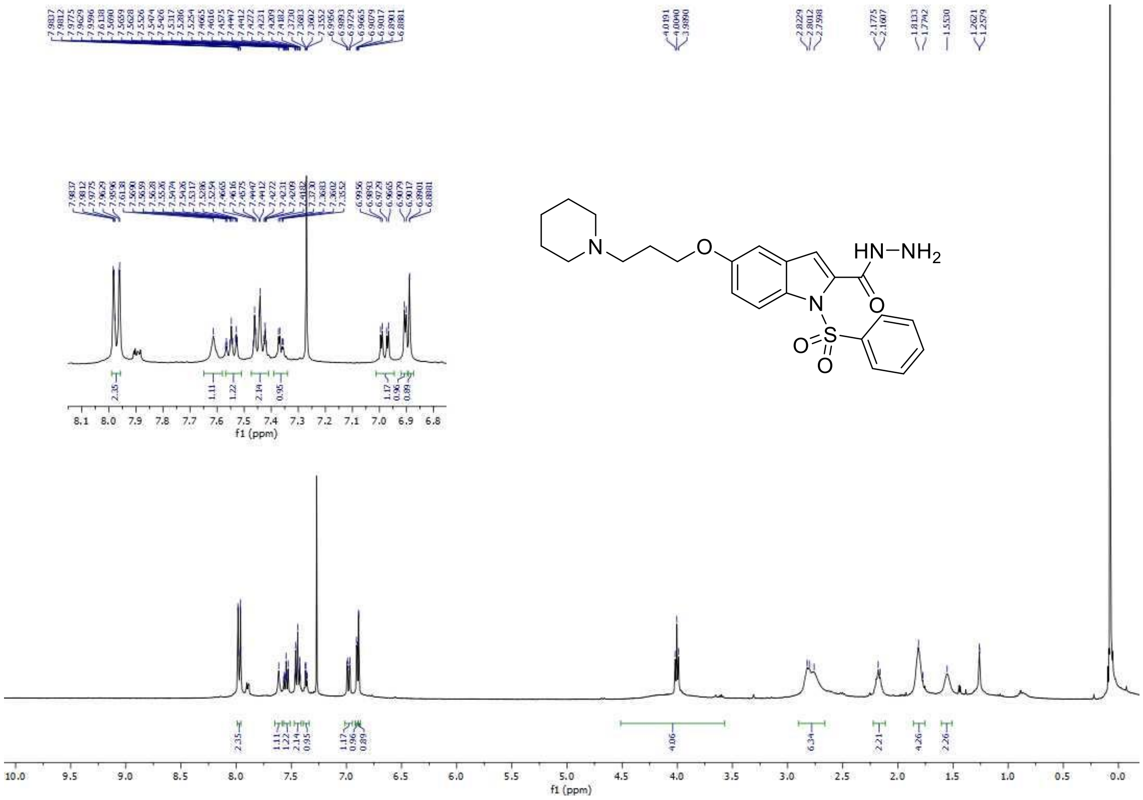

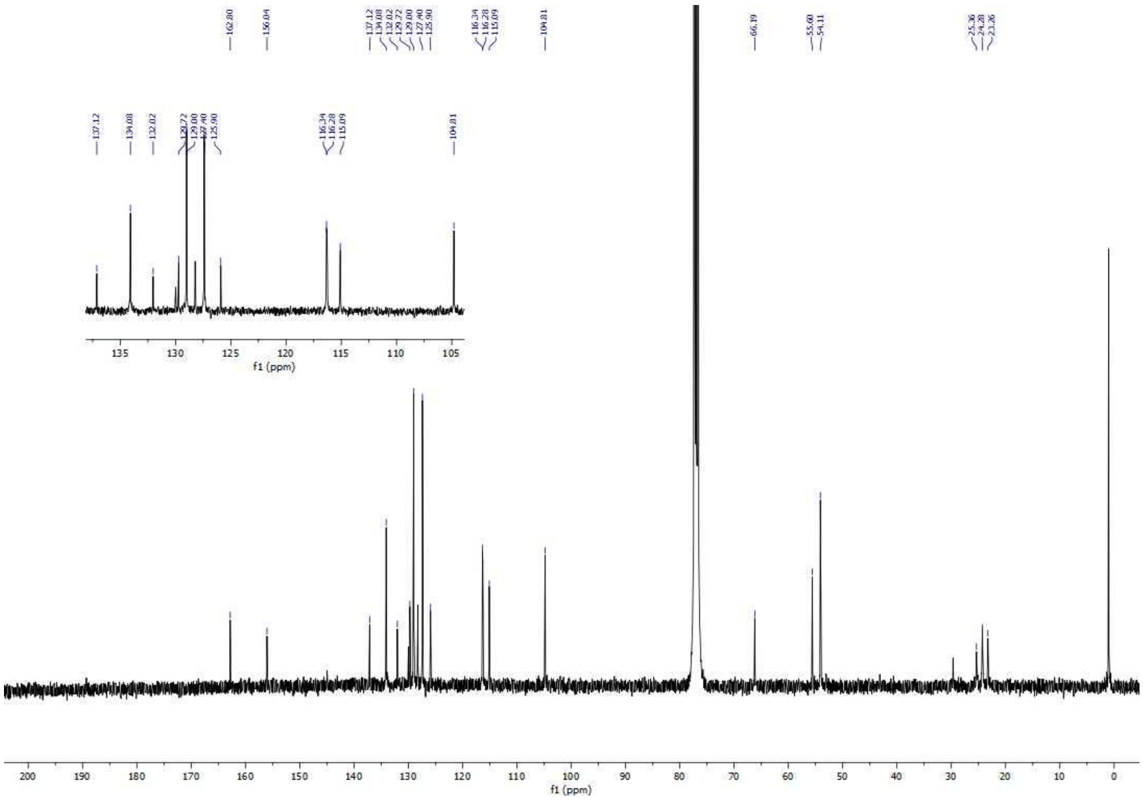

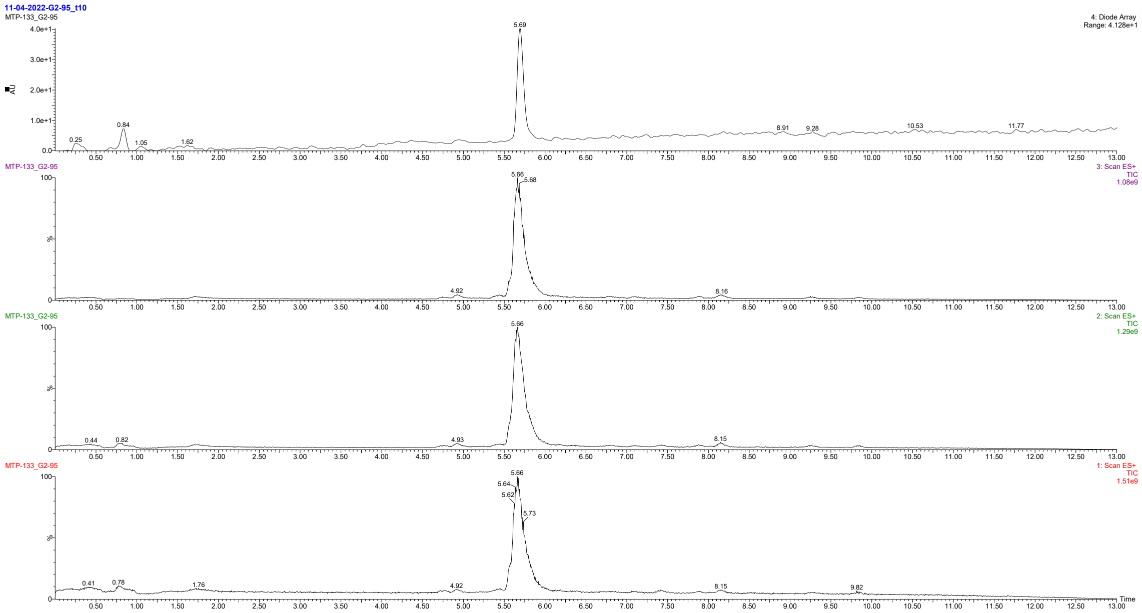

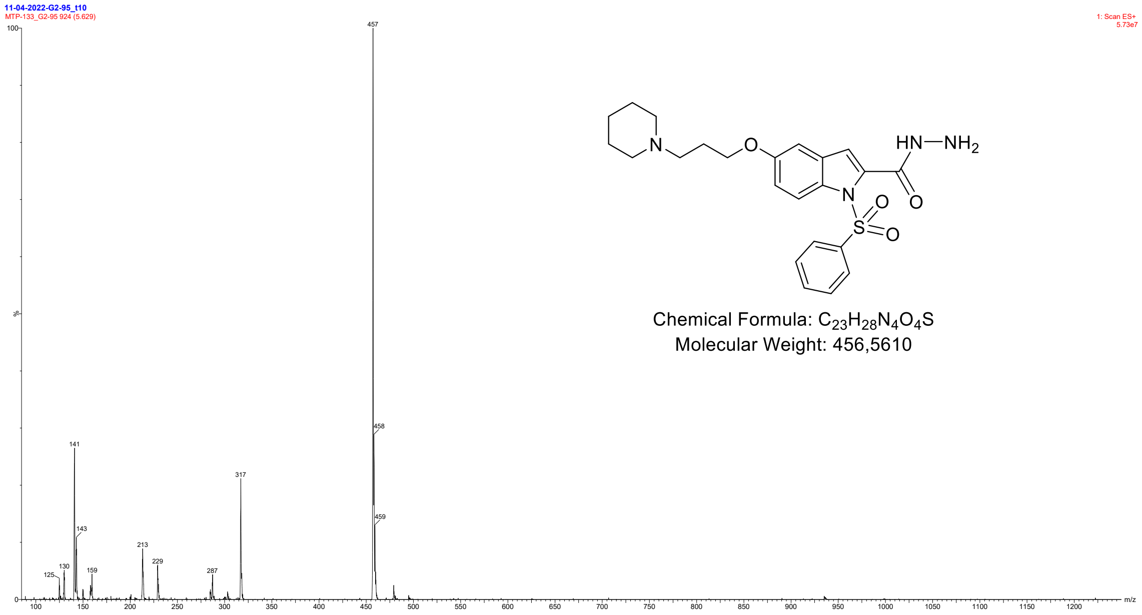

Chemical Formula: $C_{23}H_{28}N_4O_4S$

Molecular Weight: 456,5610

1-(Phenylsulfonyl)-5-(3-(4-(prop-2-yn-1-yl)piperazin-1-yl)propoxy)-1*H*-indole-2-carbohydrazide (4)

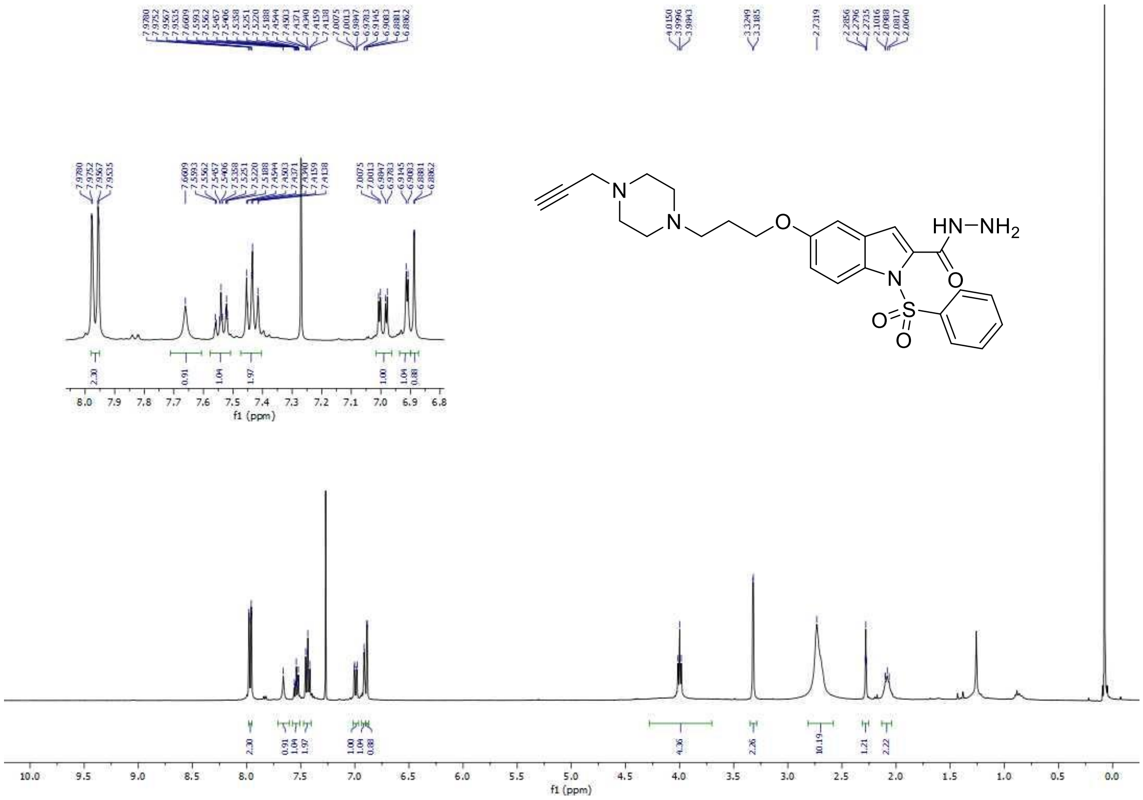

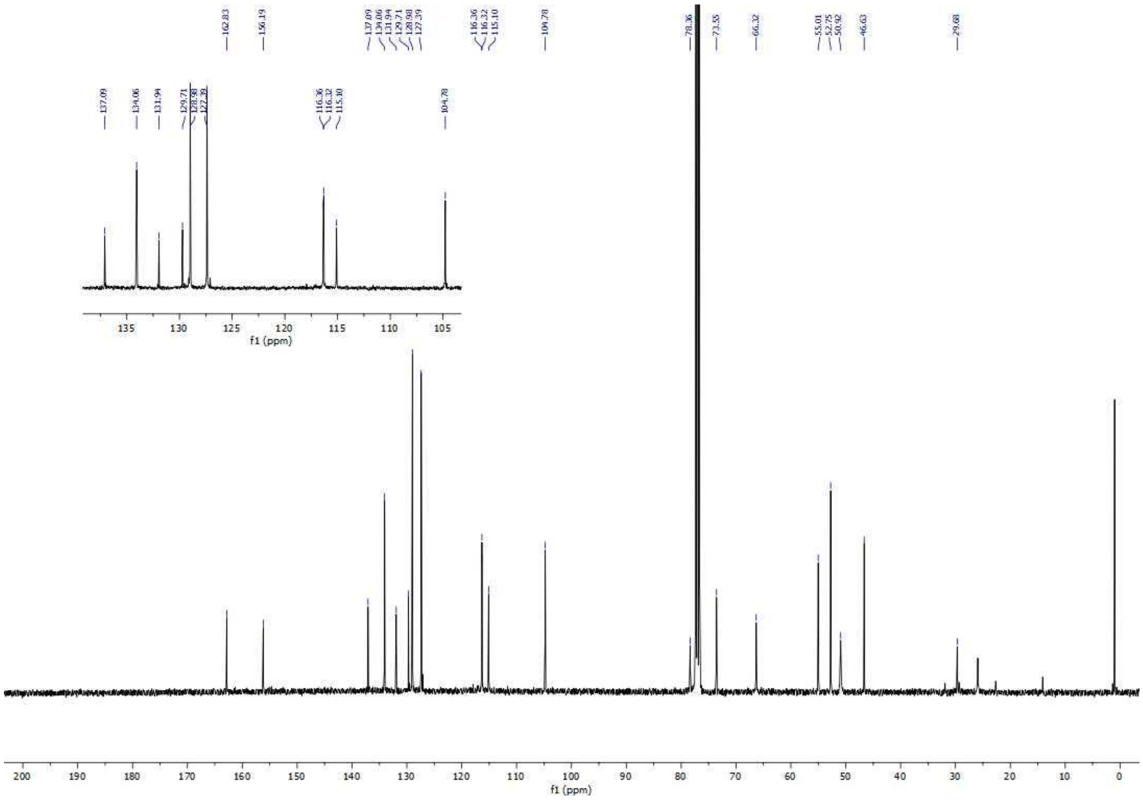

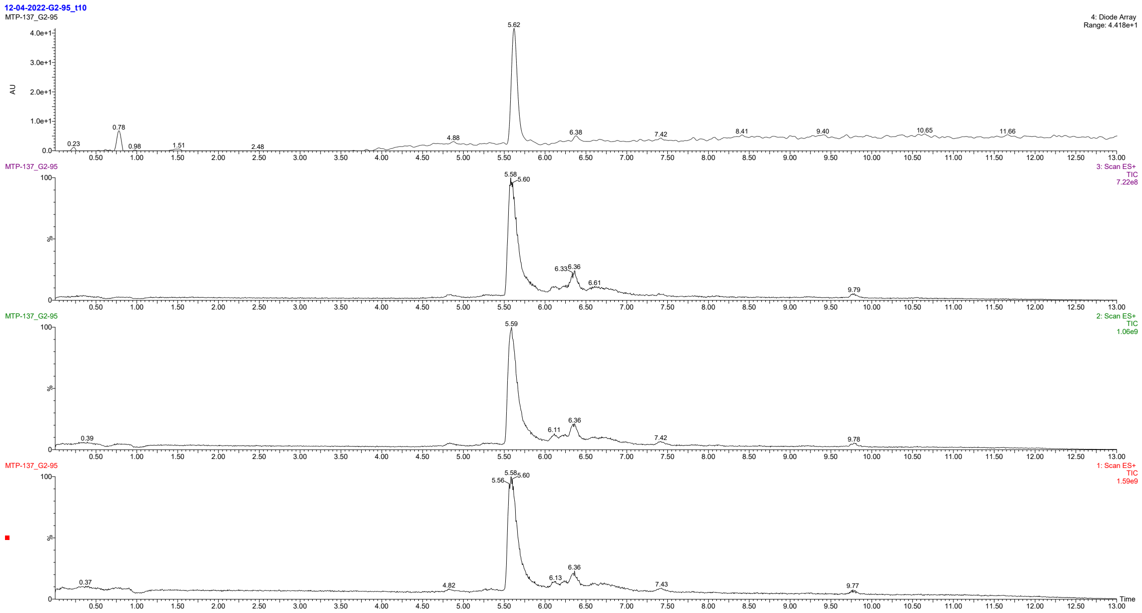

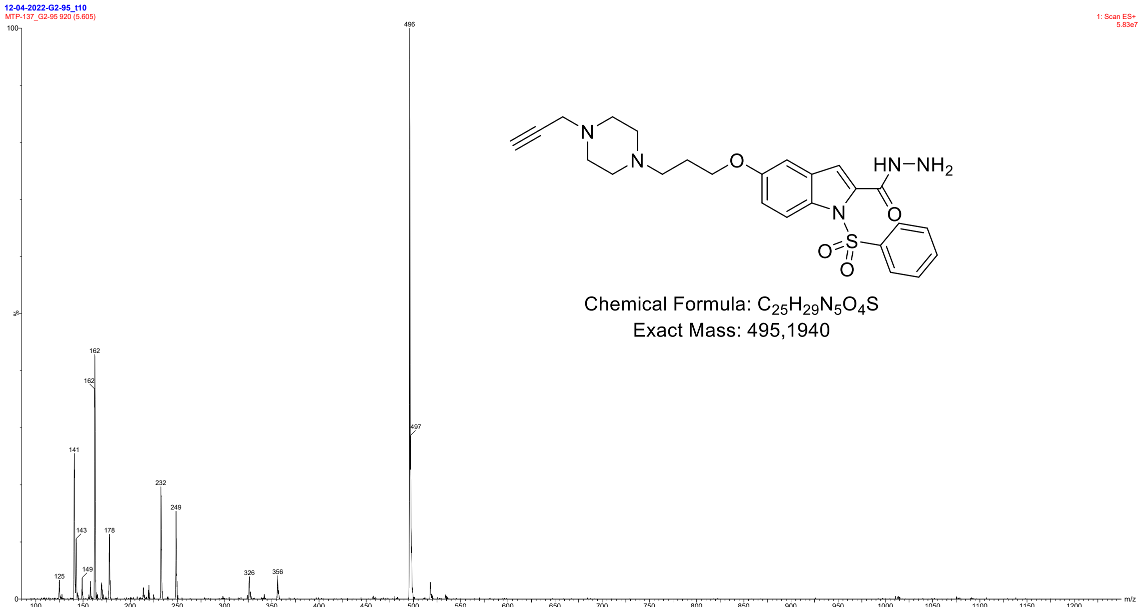

Chemical Formula: $C_{25}H_{29}N_5O_4S$
Exact Mass: 495,1940

N-(2-Aminophenyl)-5-(benzyloxy)-1-(phenylsulfonyl)-1H-indole-2-carboxamide (5)

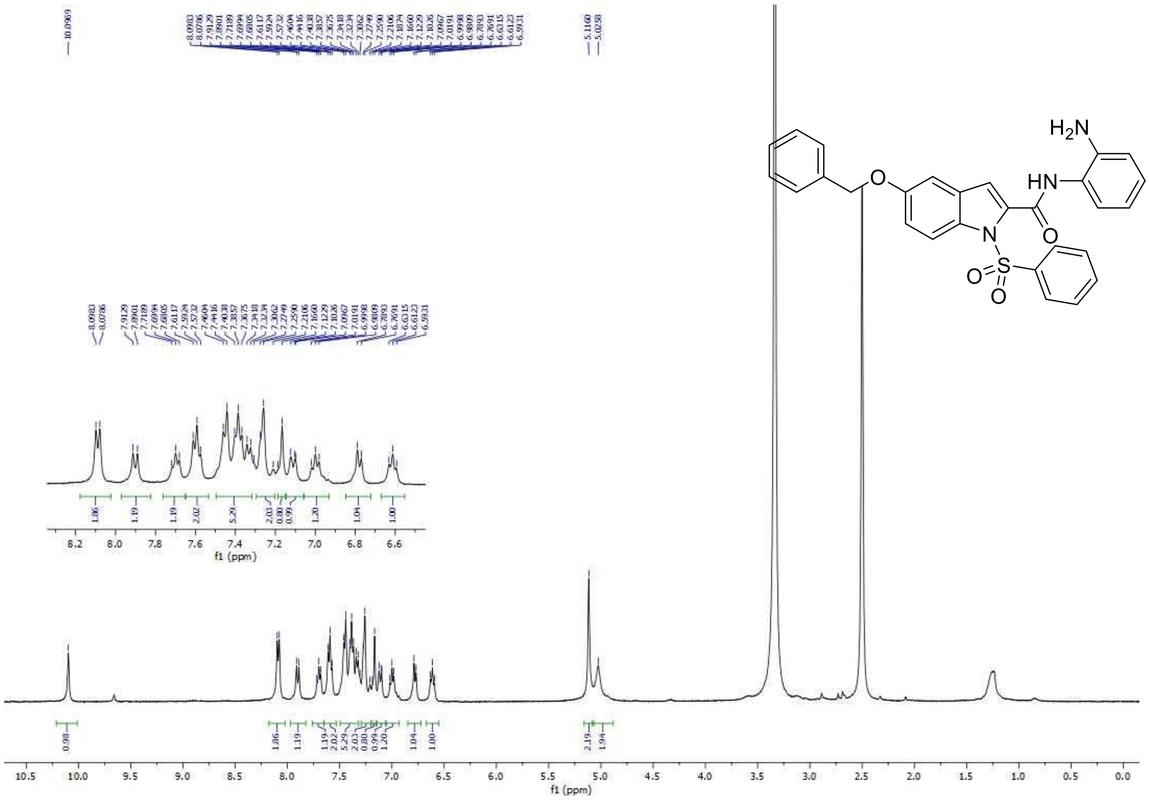

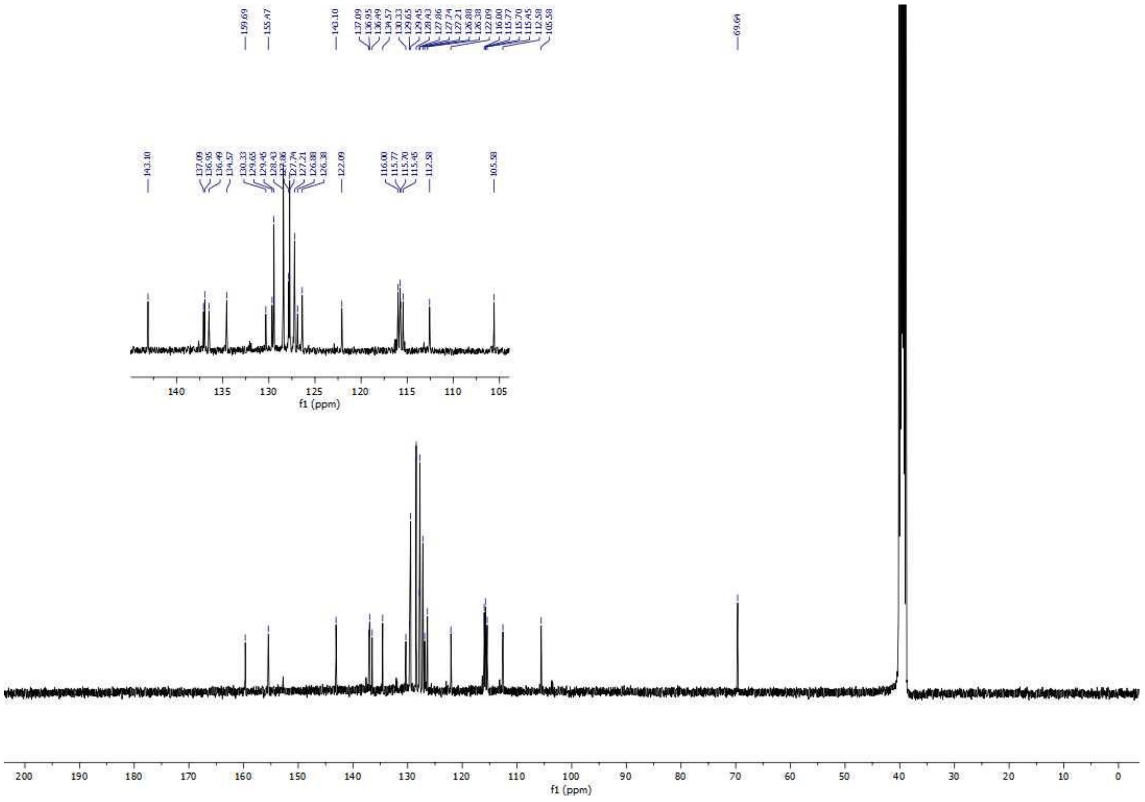

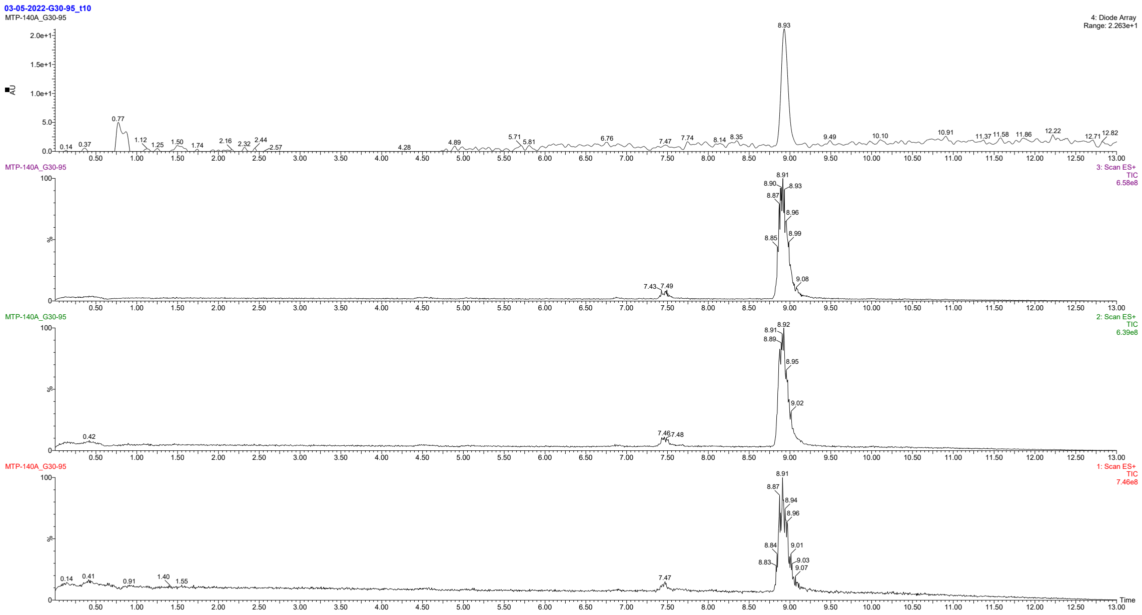

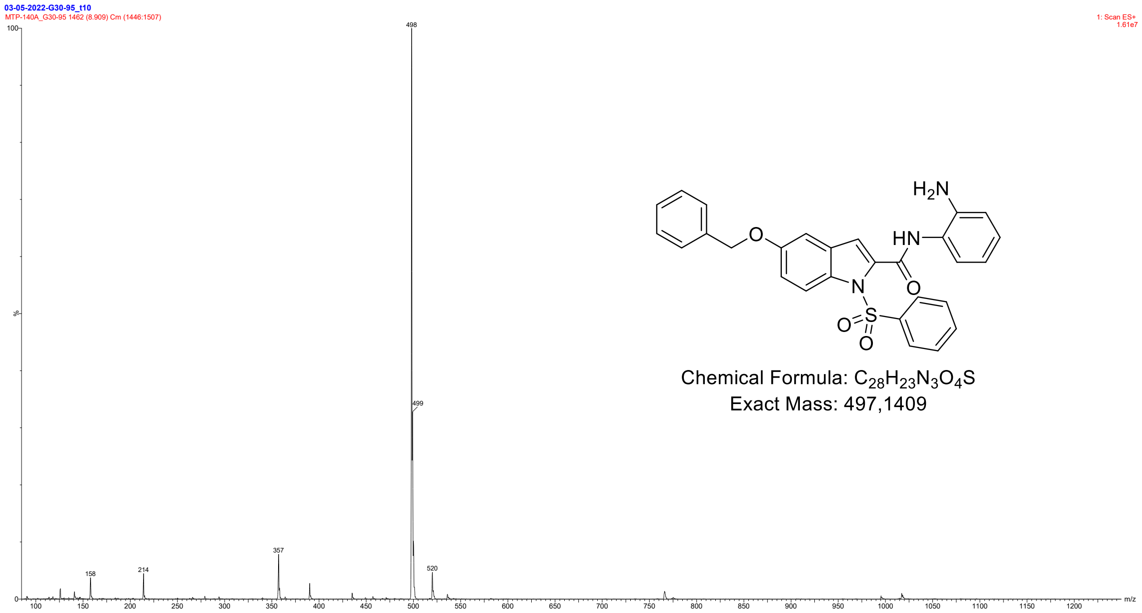

Chemical Formula: $C_{28}H_{23}N_3O_4S$

Exact Mass: 497,1409

N-(2-Aminophenyl)-1-(phenylsulfonyl)-5-(3-(piperidin-1-yl)propoxy)-1H-indole-2-carboxamide (6)

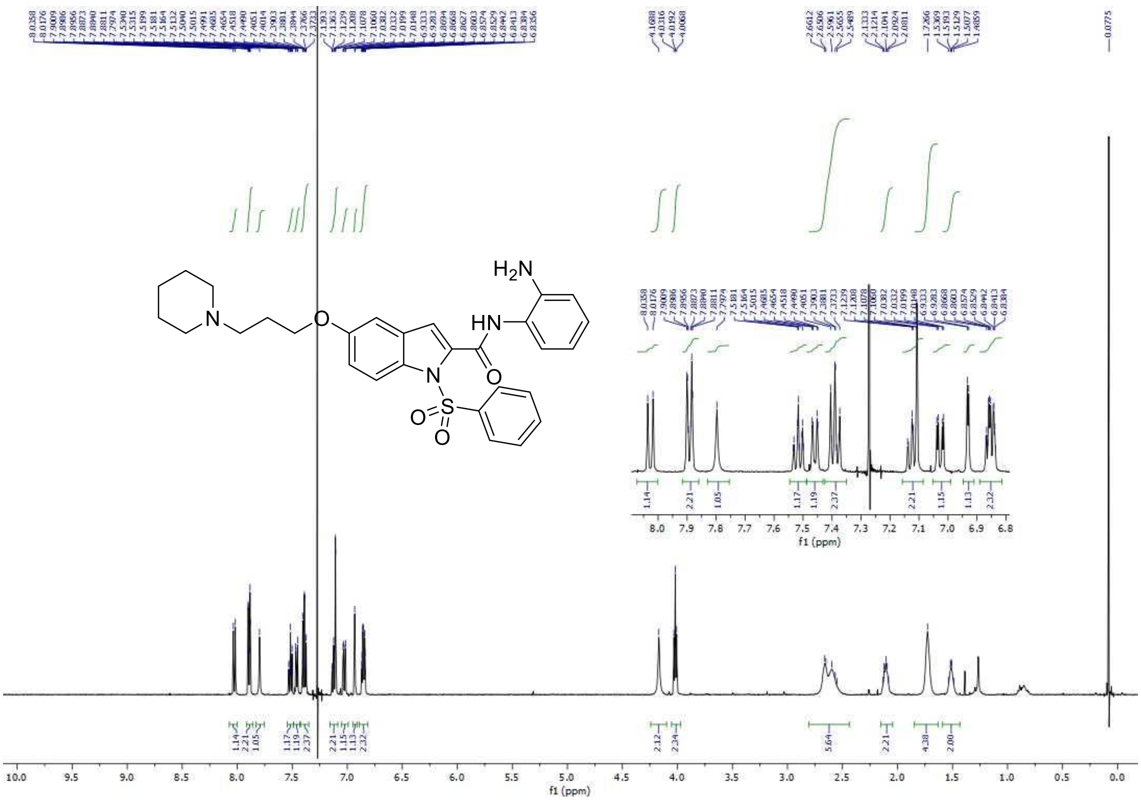

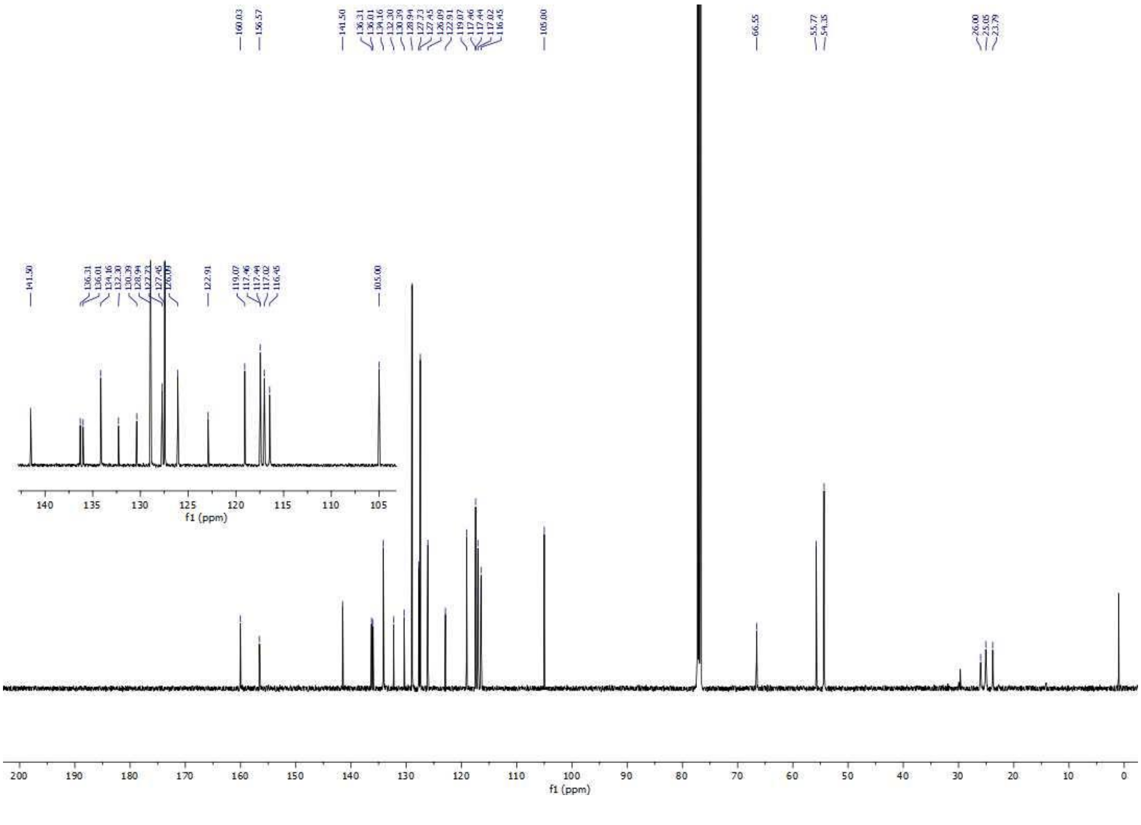

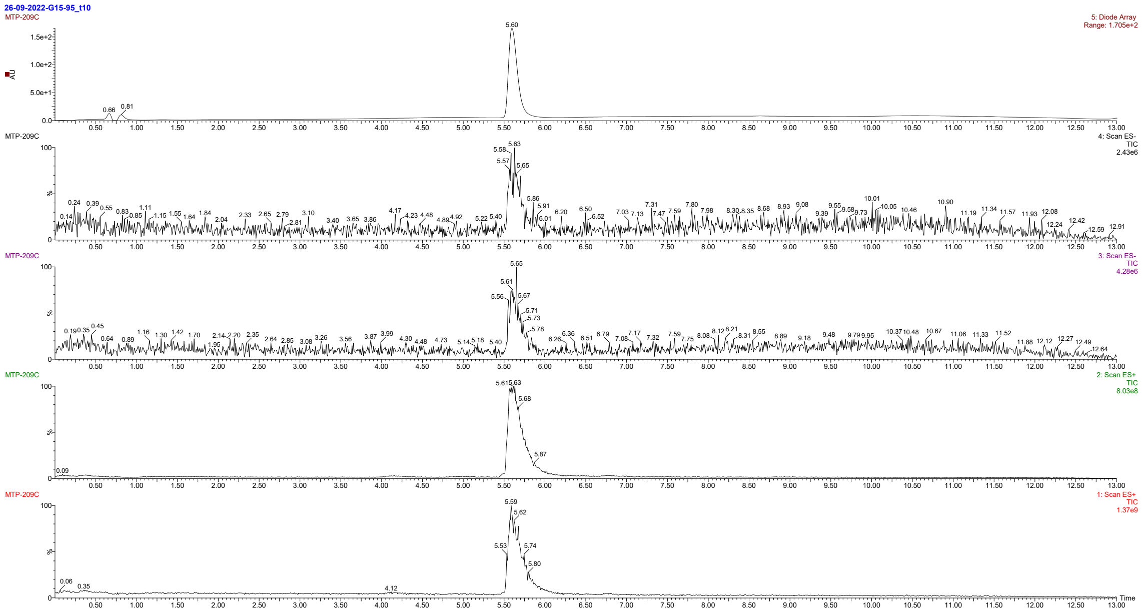

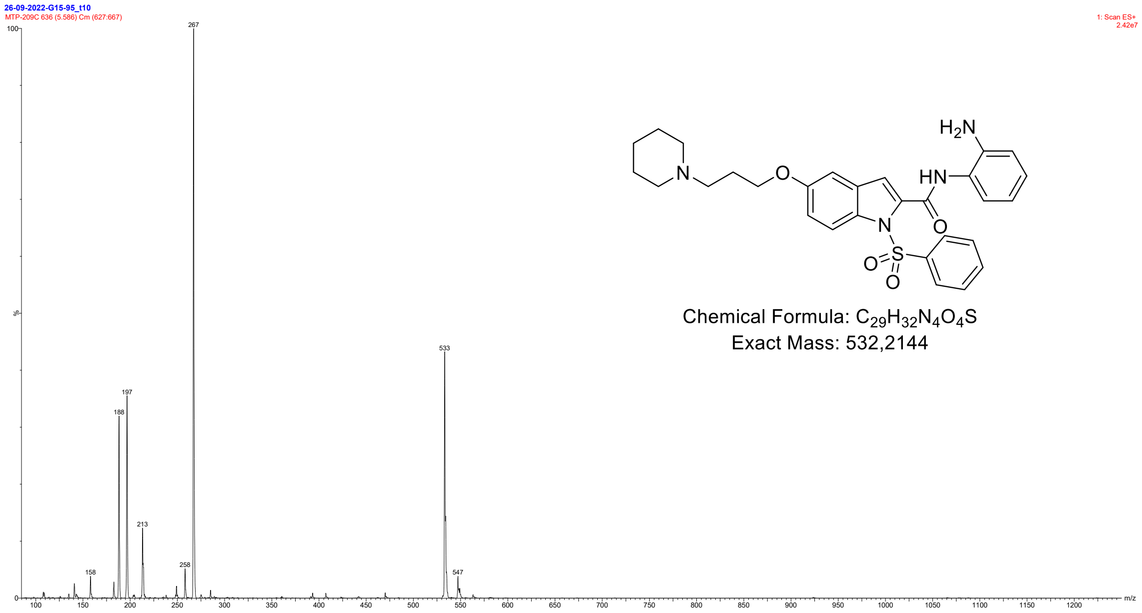

Chemical Formula: $C_{29}H_{32}N_4O_4S$

Exact Mass: 532,2144

***N*-(2-Aminophenil)-1-(phenylsulfonyl)-5-(3-(4-(prop-2-yn-1-yl)piperazin-1-yl)propoxy)-1*H*-indole-2-carboxamide (7)**

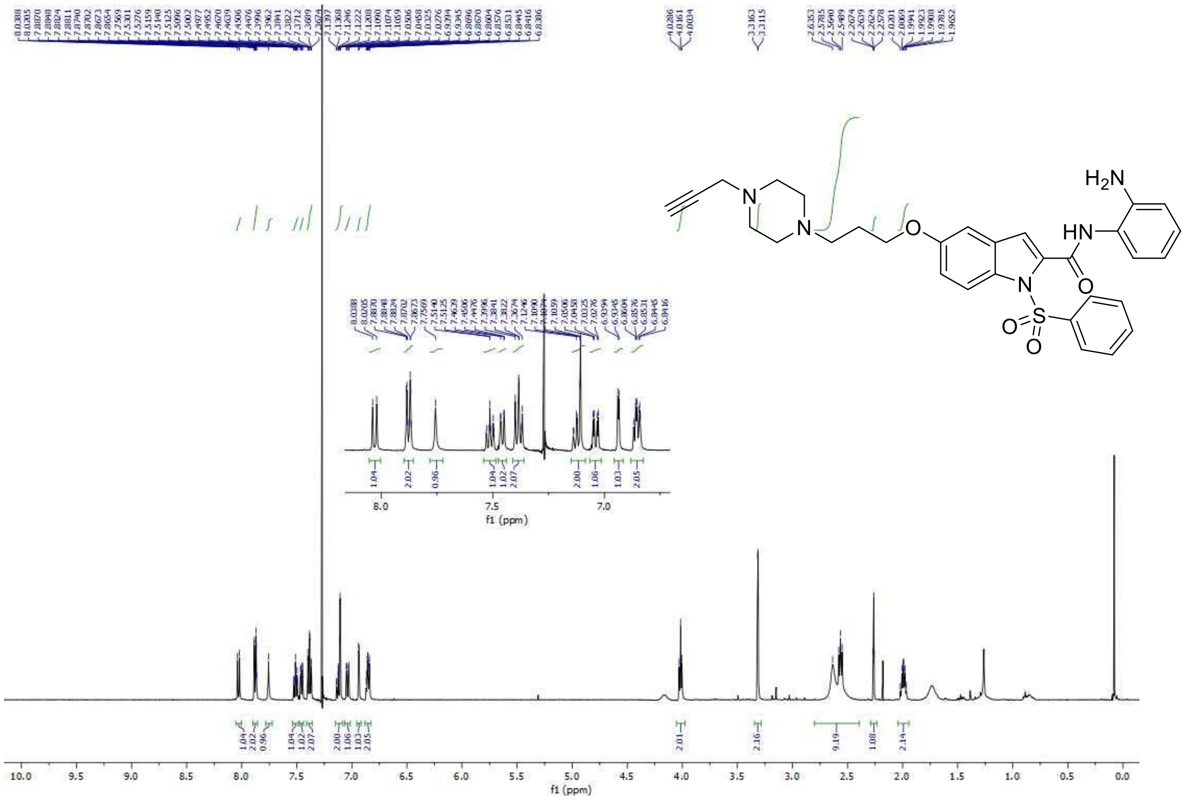

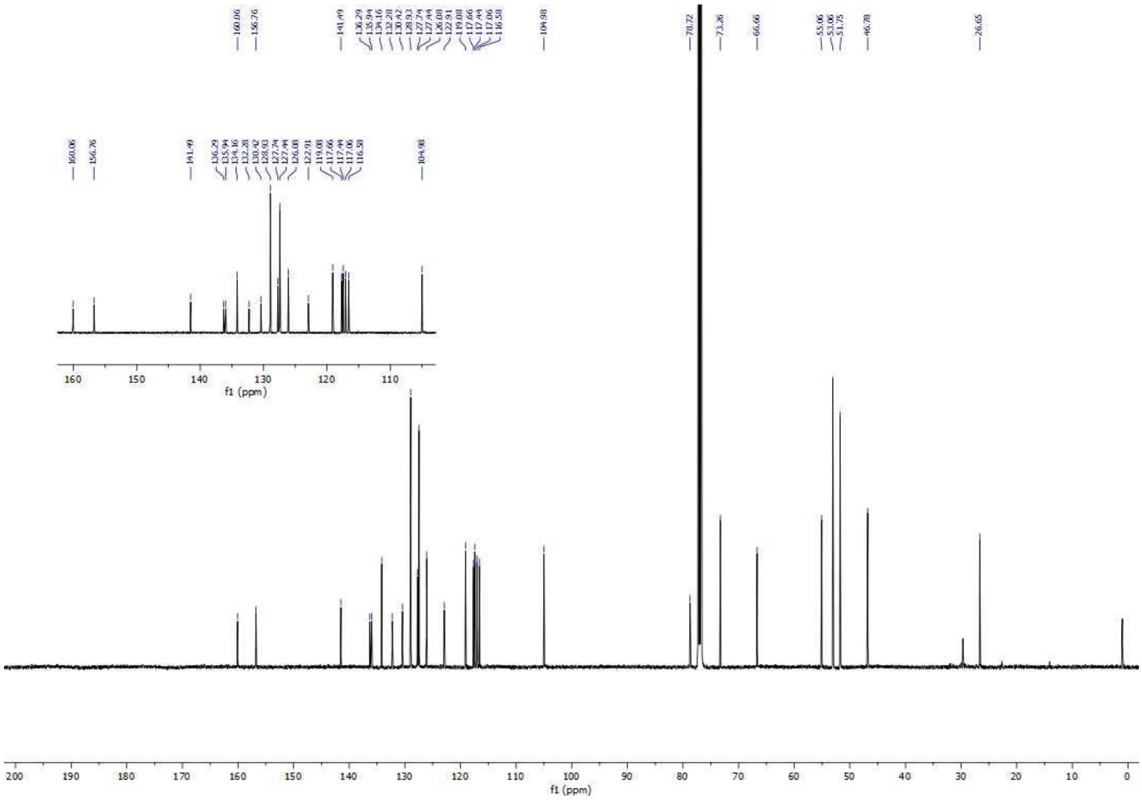

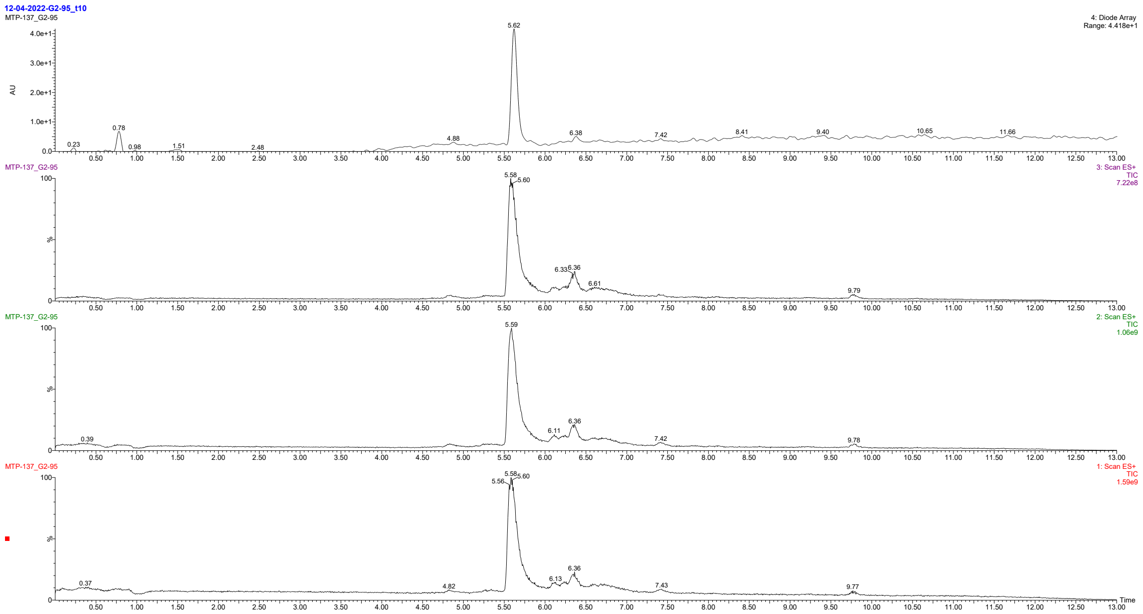
12-04-2022-G2-95_t10
MTP-137_G2-95
4: Diode Array
Range: 4.418e+1
AU
3: Scan ES+
TIC
7.22e8
2: Scan ES+
TIC
1.06e9
1: Scan ES+
TIC
1.59e9
Time

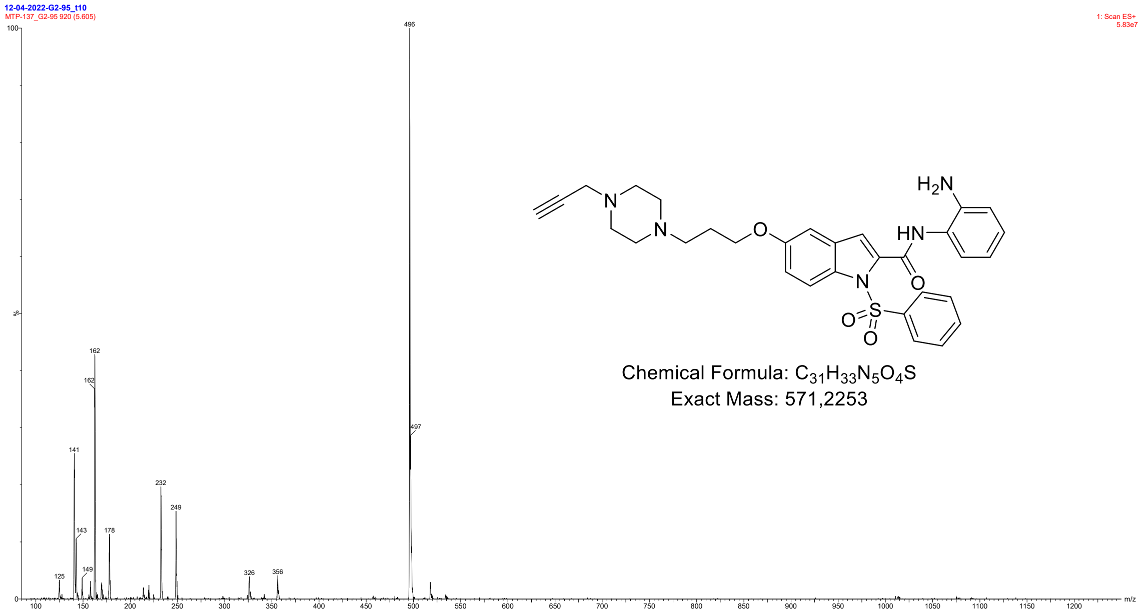
12-04-2022-G2-95_t10
MTP-137_G2-95 920 (5.605)
1: Scan ES+
5.83e7
496
497
162
141
232
249
178
143
149
125
326
356
m/z
H2N
HN
N
O
S
Chemical Formula: C31H33N5O4S
Exact Mass: 571,2253

2. Molecular docking of compound 2 into the *h*AChE, *h*BuChE, *h*HDAC1, *h*HDAC6 and *h*5-HT6R

Compound **2** is able to enter the enzyme tunnel of *h*AChE (Figure S1) and *h*BuChE (Figure S2) and interacts with the peripheral anionic site (PAS) but does not interact efficiently with the catalytic triad amino acids (CAS, *h*AChE: His447, Ser203, Glu;334 *h*BuChE: His438, Ser198, Glu325). This suggests that, although the compound can access the enzyme's gorge, it fails to establish the necessary interactions for effective binding and enzymatic inhibition.

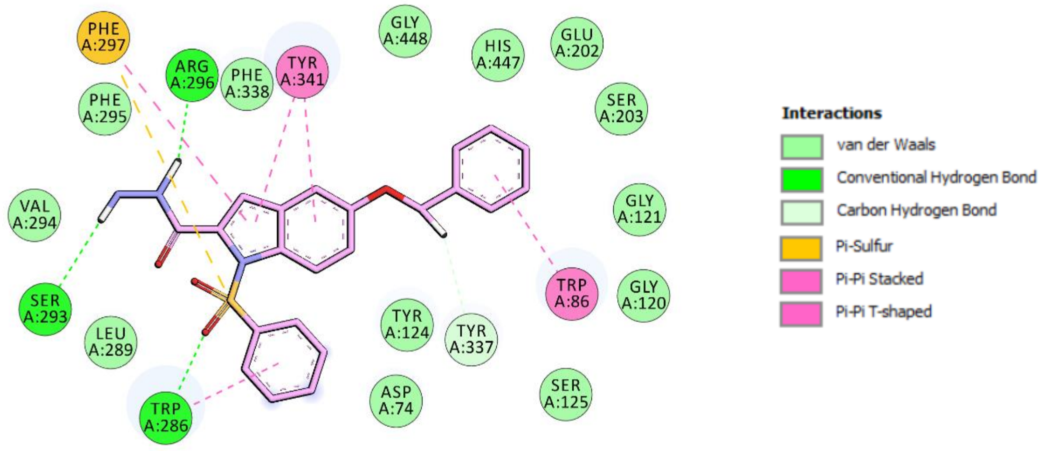

Figure S1. 2-D representation of the interactions of ligand **2** (pink) in the active site of AChE.

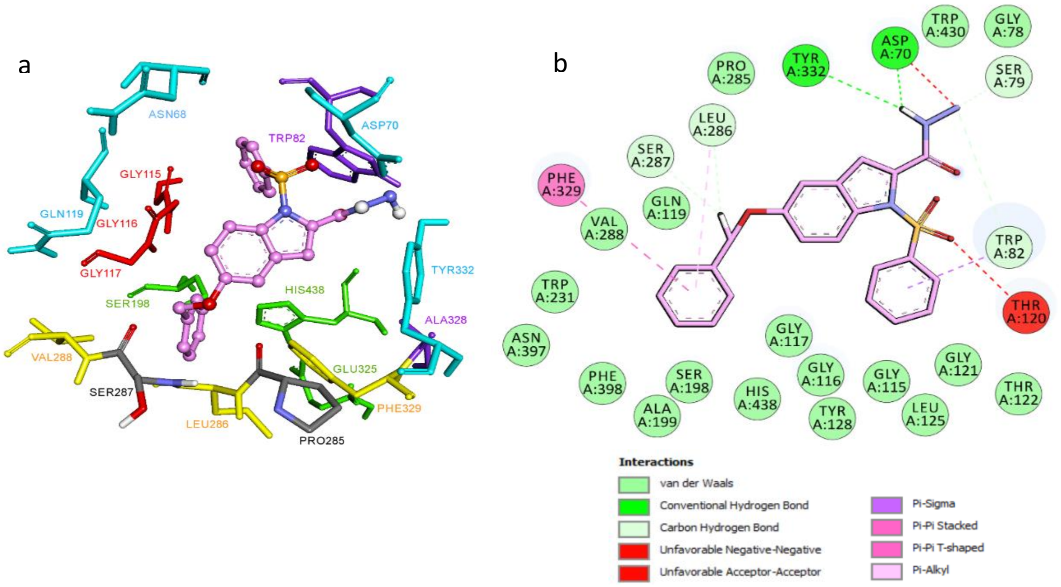

Figure S2. Proposed binding mode for compound **2** inside gorge cavity of hBuChE (PDB ID 4BDS). a) Compound **2** is colored in pink balls and sticks. Different subsites of the active site were colored: CAS in green, oxyanion hole in red, in violet choline binding site, acyl-binding pocket in yellow and PAS in blue. b) Interaction of **2** with binding pocket of hBuChE in 2D model.

Similarly, compound **2** remains at the entrance of the enzyme tunnels of HDAC1 (Figure S3) and HDAC6 (Figure S4) and does not interact with the active site. This indicates that it fails to reach or properly engage with the key residues required for effective binding and inhibition, potentially due to steric hindrance, suboptimal molecular flexibility, or insufficient interactions that drive deep binding into the active site.

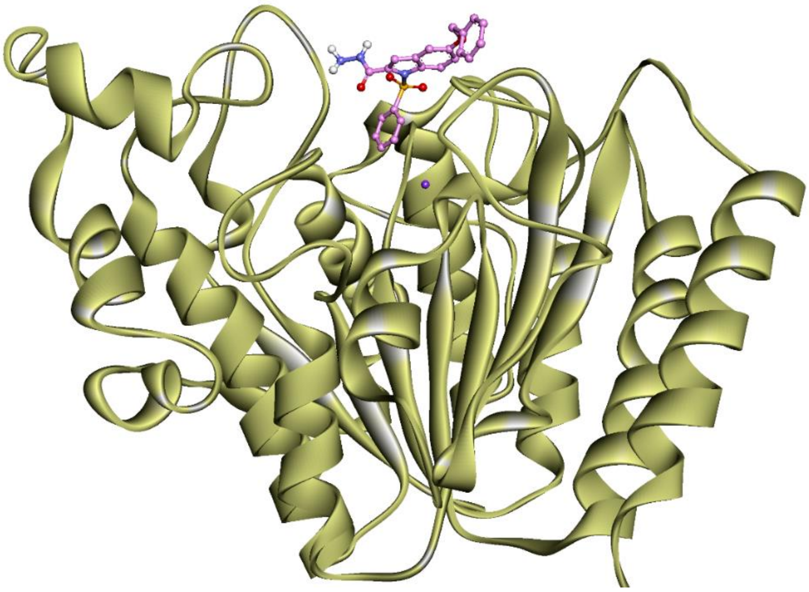

Figure S3. Proposed binding mode for compound **2** (balls and sticks, coloured in pink) with hHDAC1 (yellow) (PDB ID 4BKX). The Zn ion is depicted as a violet sphere.

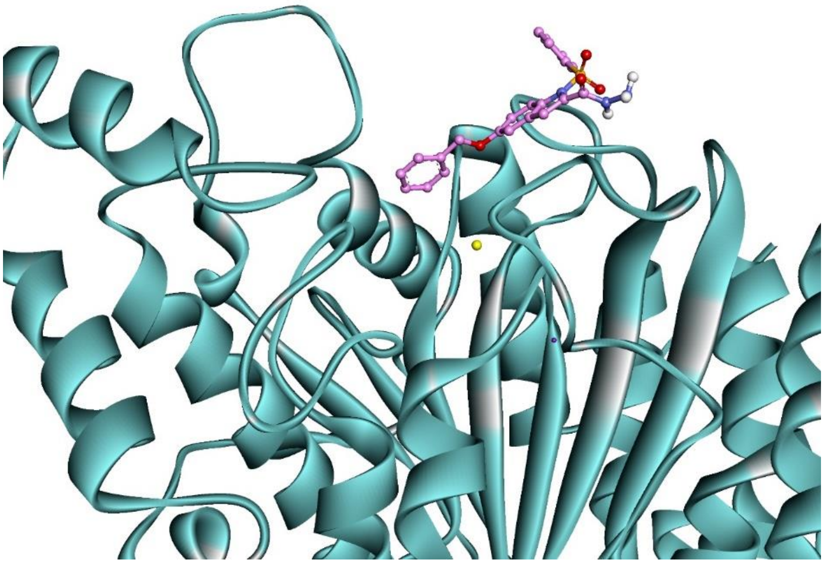

Figure S4. Proposed binding mode for compound **2** (balls and sticks, coloured in pink) with hHDAC6 (blue) (PDB ID 5EDU). The Zn ion is depicted as a yellow sphere.

Furthermore, compound **2**, although it enters the receptor tunnel of the 5-HT6R (Figure S5a) exhibits low binding affinity (-8.2 kcal/mol) and does not interact with the key amino acids crucial for ligand binding and receptor activation, such as Asp106 (Figure S5b). Due to this behaviour, it is unlikely to effectively modulate or inhibit the receptor's activity.

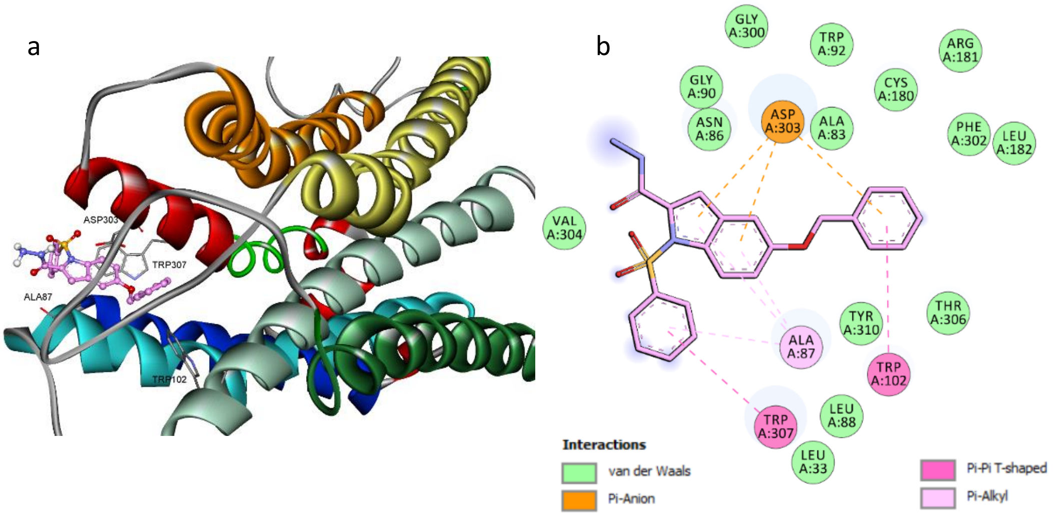

Figure S5. Predicted binding mode of compound **2** (pink) in the active site of the 5-HT6R. a) Compound **2** is docked into 5-HT6R. Residues identified as crucial for ligand binding are represented as thick sticks**. b)** Interaction of **2** with the binding pocket of 5-HT6R in 2D model.

In summary, the lack of effective binding to the key amino acids in the active sites or crucial interaction sites of AChE, BuChE, HDAC1, HDAC6, and 5-HT6 likely correlates with the observed low or absent inhibition in the experimental assays.

3. Molecular docking of compound 6 in *h*BuChE

According to the docking results, compound **6** exhibits high binding affinity (-10.3 kcal/mol) against BuChE and it is able to fit well in active site of the enzyme and interacts with important amino acid residues (Figure S6a). This compound could engage with the catalytic triad of BuChE via carbon hydrogen and van der Waals interactions with residues Hist438 and Ser198, respectively (Figure S6b). In addition, it formed essential connections with amino acid residues Asn68, Gln119 and Tyr332 in the peripheral site, as well as with residues Gly116 and Gly117 in the oxyanion hole (OH) of BuChE via van der Waals and carbon hydrogen interactions, respectively. The compound also linked with the acyl loop (ABP) of the target enzyme through a π-alkyl interaction with Leu286 and van der Waals interactions with Val288, Phe329. Furthermore, it formed hydrogen bonds with Ser287, while engaging in π-cation, π-sigma and π-alkyl interactions with residue Trp82 and in van der Waals interaction with Ala328, choline binding site (CBS). Finally, attractive charge interaction is established between the piperidinium moiety and Glu197 (Figure S6b).

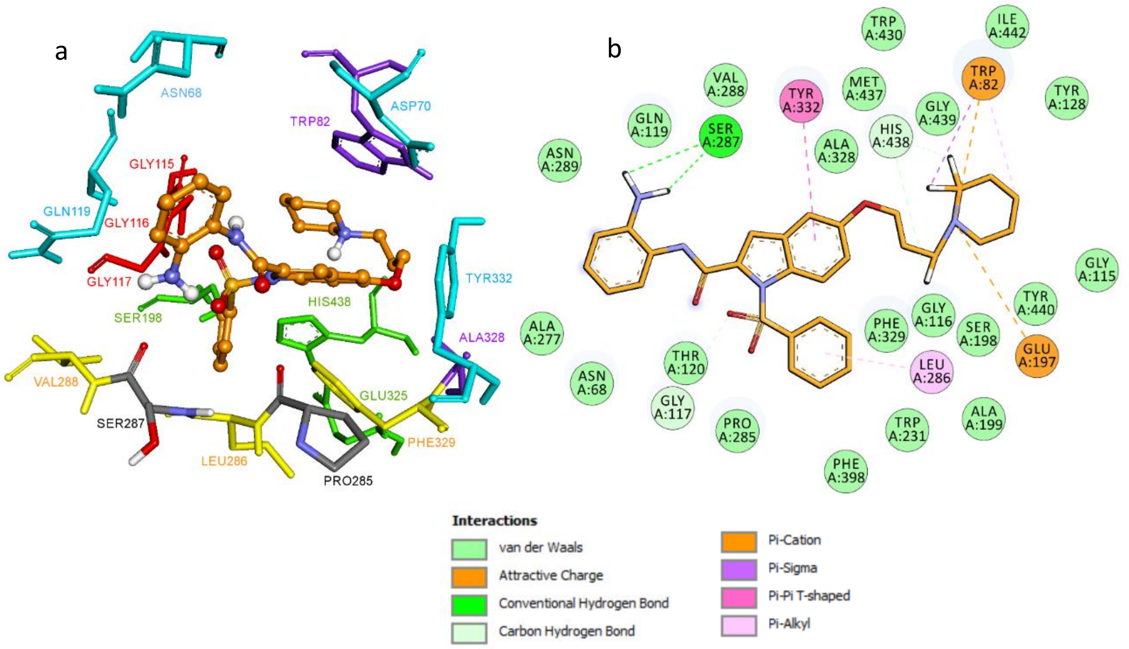

Figure S6. Proposed binding mode for compound **6** inside gorge cavity of *h*BuChE. a) Compound **6** is colored in orange. Different subsites of the active site were colored: CAS in green, OH in red, in violet CBS, ABP in yellow, and PAS in blue. b) Interaction of **19** with binding pocket of hBuChE in 2D model.

4. Molecular modelling compound MTP150 on HDAC1,6

To investigate the binding mode of compound **MTP150** in the active sites of HDAC1 and HDAC6, we conducted a molecular docking study. Using Autodock Vina[1], we docked the compound into the available crystal structures of HDAC1 (PDB ID: 4BKX) and HDAC6 (PDB ID: 5EDU). The crystal structures of HDAC enzymes feature an active Zn^{+2} ion at the bottom of the pocket, a hydrophobic channel leading to this Zn^{+2} ion, and a surface rim at the pocket entrance, known as the cap domain. HDAC6 has a distinct pocket formed by a loop and a helix. In this configuration, residue Trp496 on the loop interacts through van der Waals forces with residue His560 on the helix, causing the loop to extend towards the helix and creating the specific pocket. The cap domain differs significantly in both shape and properties between HDAC6 and HDAC1. Additionally, the channel in HDAC6 is broader and shallower compared to that of HDAC1, suggesting that larger and more rigid cap groups may preferentially bind to HDAC6.

Compound **MTP150** incorporates an α,β-unsaturated motif at the C3' position of the *N*(1)-sulfonamide aromatic ring, acting as a linker. This linker is attached to a hydroxamic acid group for Zn^{2+} chelation, and the structure is completed with a bulky moiety serving as the cap.

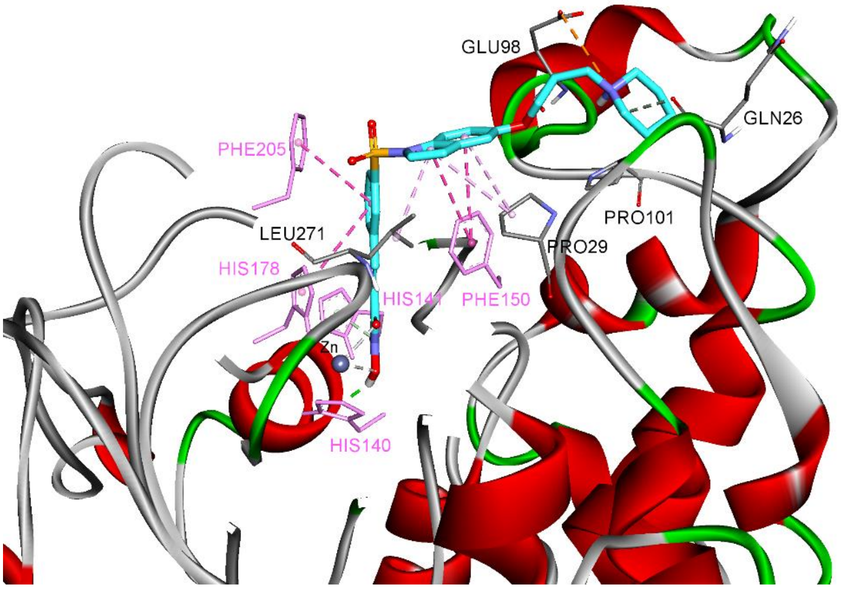

Figure S7. Compound **MTP150** in HDAC1. Binding energy: -9.8 kcal/mol. The ligand is shown as blue sticks, side chains of binding site residues as color code and flexible residues in pink. Green dashed lines indicate hydrogen bond interactions and grey dashed lines metal coordination. Zn ion is shown as a purple sphere.

As illustrated in Figure S7, compound **MTP150** is capable of penetrating deeply into the enzyme gorge, where the orientation of the hydroxamic group allows it to interact with the zinc ion (Figure S7) in a bidentate binding mode in which the hydroxy group and carbonyl oxygen bind the zinc center. The compound also engages in interactions with Asp176 and Asp264 (via van der Waals interactions), and with His178 (through π-π interactions). The coupling of these three amino acids with the compound is particularly significant due to their coordination with the zinc anion. Additionally, interactions occur with Phe150 (via π-π interactions), His140 (via hydrogen bonding), His141 (via π-donor hydrogen bond) and Tyr303 (through van der Waals interactions). It is noteworthy that the last three amino acids play a crucial role in the deacetylation mechanism, with the histidines being involved in charge transfer to the active site.
Moreover, the alkyl-piperidine-indole cap engages with the rim of the cavity, facilitating π-alkyl interactions with Pro29 and Leu271, π-π interactions with Phe150, alkyl interactions with Pro101, a carbon-hydrogen bond with Gln26 and attractive charge with Glu98. These interactions are further strengthened by van der Waals interactions involving Tyr24, Gly25, His28 and Asp99 (Figure 8S).

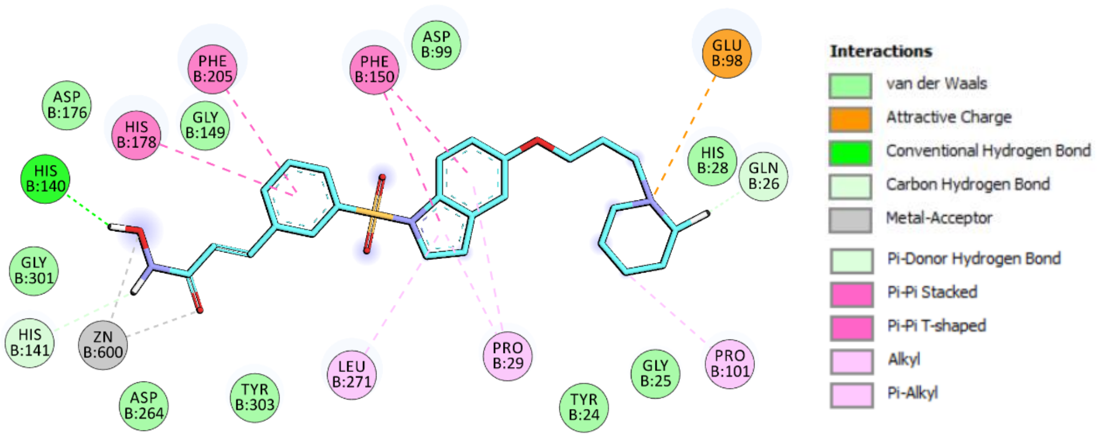

Figure S8. Interaction of compound **MTP150** with binding pocket of HDAC1 in 2D model.

The docking study in HDAC6 displayed that the hydroxamic group of compound **MTP150** chelated the Zn^{+2} ion in the bottom of the pocket in a monodentate fashion using its carbonyl group, currently engaging in a hydrogen bond with His610 (Figure S9). This residue is involved in the catalytic core of HDAC6 and form similar interactions with

well-known HDAC inhibitor trichostatin A. The hydroxamate OH group formed carbon hydrogen bond with Gly780 (Figure S9). Moreover, the NH of hydroxamic tail formed hydrogen bond with Tyr782. In the linker region, the benzene group formed π-π stacked interactions with Phe680, and with Phe620. With respect to the cap region, compound **MTP150** does not bind to the specific pocket (Trp496, Asn494 and His560), but instead the indole-ether-piperidine cap contacts the cavity rim where it can elicit π-π interactions with Phe679 and Phe680 and attractive charge interaction with Asp675 (Figure S10).

The docking scores calculated for compound **MTP150** in complex with HDAC1 and HDAC6 suggest good affinity towards both isozymes. However, a detailed analysis reveals distinct binding modes and coordination with the zinc ion, indicating potential differences in biological activity. In HDAC1, compound **MTP150** coordinates the zinc ion in a bidentate fashion, while in HDAC6, it binds only the zinc *via* the carbonyl group of the hydroxamic acid. Docking models indicate that in HDAC6, the interactions of the cap group with the enzyme surface hinder the proper approach of the metal-binding moiety to the zinc ion preventing bidentate coordination, resulting in a monodentate interaction which explains the lower inhibitory activity of **MTP150** against HDAC6 compared to HDAC1, while noting that docking provides a qualitative view of ligand–target interactions.

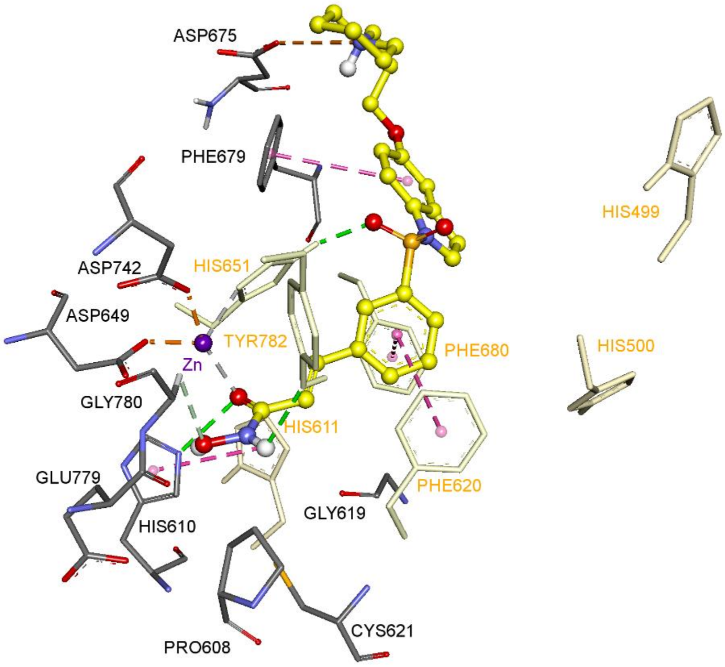

Figure S9. Predicted binding mode of compound **MTP150** in HDAC6. Binding energy: -9.8 kcal/mol. The ligand is shown as yellow ball and sticks, side chains of binding site residues as color code and flexible residues in the same color than ligand. Zn ion is shown as a purple sphere. Green dashed lines indicate hydrogen bond interactions and grey dashed lines metal coordination.

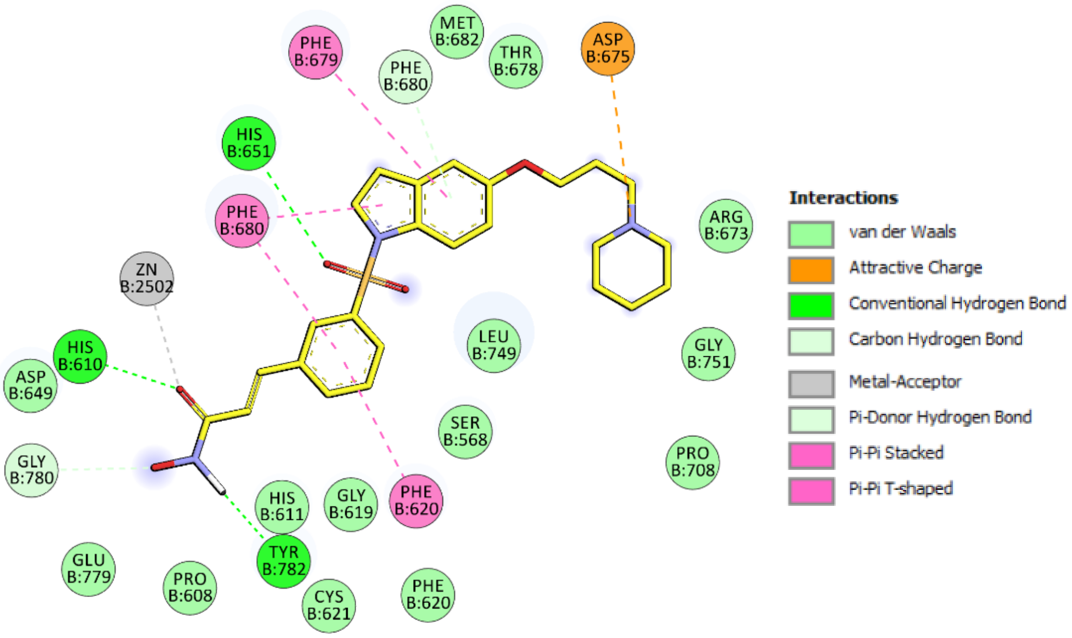

Figure S10. 2D representation of the amino acids in the HDAC6 binding site interacting with compound **MTP150**.

5. ADME of compound MTP150

The ADME (Absorption, Distribution, Metabolism, and Excretion) properties of compound **MTP150** were theoretically evaluated using the QikProp module from the Schrodinger suite (QikProp, Schrodinger, LLC, New York, NY, 2024). These calculations were performed in normal mode to assess the druggability of the compound. A comprehensive set of physically significant descriptors and pharmacologically relevant properties were predicted and subjected to detailed analysis, as summarized in Table S1. The findings indicate that compound **MTP150** does not violate Lipinski's rule of five[2] (ROF) or the QikProp rule of three[3,4] (ROT). Most of the predicted descriptors and properties fall within the acceptable QikProp thresholds, with the exception of the estimated number of hydrogen bonds accepted (accptHB) by the solute.

Aqueous solubility (QPlogS) of organic molecules significantly influences various ADME-related properties. For compound **MTP150**, the solubility values were within the acceptable range (QPlogS = -4.215; acceptable limits: -6.5 to 0.5). Additionally, the partition coefficient (QPlogPo/w), which is crucial for estimating absorption within the body, was also found to be within the recommended range (QPlogPo/w = 2.148; acceptable limits: -2.0 to 6.5) (Table S1).

Among the various properties evaluated, the predicted Blood-Brain Barrier (BBB) penetration (QPlogBB: acceptable range −3.0 to 1.2) is of particular importance, as it indicates the molecule's ability to traverse the blood-brain barrier, a critical requirement for Alzheimer's disease treatments. The predicted QPlogBB value for compound **MTP150** is -2.075, placing it within the optimal penetration range (see Table S1). Additionally, the Polar Surface Area (PSA), which measures the molecule's hydrogen bonding capacity, must remain below a certain threshold for CNS activity. For compound **MTP150**, the PSA is 113.587 Å^2, which is within the acceptable range. Furthermore, the compound exhibits moderate human oral absorption at 65.772 % (see Table S1).

The additional physicochemical descriptors generated by QikProp (Table S1) fall within the acceptable range for human use. Consequently, this study indicates that the designed compound possesses suitable pharmacokinetic properties, rendering it a promising candidate for use as a drug, including potential applications as a CNS-active agent.

Table S1. Physicochemical properties for compound **MTP150** calculated using Qikprop

MW	SASA	volume	donorHB	accptHB	QPlogPo/w	QPlogS
483.581	837.694	1496.244	2.000	11.450	2.148	-4.215

QPPCaco	PSA	QPlogBB	metab	QPlogKhsa	% HOA	ROF	ROT
29.292	113.587	-2.075	2	-0.099	65.772	0	0

MW: Molecular weight of the molecule (130.0-725.0). SASA: Total Solvent Accessible Surface Area, in square angstroms, using a probe with a 1.4 Å radius (limits 300.0-1000.0). volume: Total solvent-accessible volume, in cubic angstroms, using a probe with a 1.4 Å radius (limits 500.0-2000.0). donorHB: Estimated number of hydrogen bonds that would be accepted by the solute (limits: 2.0-20.0). accptHB: Estimated number of hydrogen bonds that would be donated by the solute (limits: 0.0-6.0). QPlogPo/w: Predicted octanol/water partition coefficient (limits -2.0-6.5). QPlogS: Predicted aqueous solubility. S, in mol/dm^3, is the concentration of the solute's saturated solution that is in equilibrium with crystalline solid (limits -6.5-0.5). QPPCaco: Predicted apparent Caco-2 cell permeability in nm/sec. Caco-2 cells is a model for the gut-blood barrier. QikProp predictions are for non-active transport. (< 25 poor, > 500 great). PSA: Van der Waals surface area of polar nitrogen and oxygen atoms (limits 7.0-200.0). QPlog BB: Predicted brain/blood partition coefficient (limits -3.0–1.2). metab: Number of likely metabolic reactions (limits 1-8). QPlogKhsa: Prediction of binding to human serum albumin (limits -1.5-1.5). HOA: Predicted qualitative Human Oral Absorption on 0 to 100% scale. ROF: Number of violations of Lipinski's Rule Of Five. ROT: Number of violations of Jorgensen's rule of three.

Experimental

Structure of protonated compounds **2**, **6**, and **MTP150** were built using Discovery Studio 2024 (DS). The molecular geometry of the ligand was energy-minimized using the adopted-based Newton–Raphson algorithm with the CHARMm force field[5] with a convergence criterion for the energy gradient 0.001 kcal (mol-Å)$^{-1}$.

The 3D structures of the enzymes were retrieved from the Protein Data Bank: human HDAC1 (PDB ID: 4BKX) and human HDAC6 in complex with trichostatin A (PDB ID: 5EDU). Because HDAC1 structure (4BKX) includes HDAC1 and metastasis-associated protein MTA1, this was excluded from de PDB structure and only HDAC1 coordinates were extracted from 4BKX. The coordinates of human ChE were also obtained from the Protein Data Bank: AChE (PDB ID: 1B41); BChE (PDB ID: 4BDS). In the case of 5-HT6R, the human 5-HT6 model in complex with serotonin- DNGs-scFv16 has been

retrieved from the PDB Repository (PDB ID: 7YS6) but only 5-HT6 coordinates were extracted from 7YS6 for docking studies.

Prior to molecular docking, receptor proteins were prepared removing water molecules, unwanted ligands, and small ions except zinc ion. Zinc was not removed because it is known to work as cofactor for some of these proteins and stabilize the structures. Proper bonds, bond orders, hybridization and charges were assigned using protein model tool in DS software package. CHARMm force field was applied using the receptor ligand interactions tool in DS software package.

The prepared protein and ligands were directly loaded into AutoDock tools (ADT), hydrogens and partial charges for both were calculated using Gasteiger charges. ADT is also used for generating input pdbqt files for Autodock Vina.

All molecular dockings were performed with flexible ligands and for HDAC1, HDAC6 and AChE systems, the incorporation of protein structural flexibility has been used by keeping the entire protein rigid and making flexible the residues involved in the catalytic channel. For HDAC1, the flexible residues were His140, His141, Phe295, Asp264, Tyr303 and Asp176. For HDAC6, the flexible residues were His499, His500, His611, His651, Phe620, Phe680 and Tyr782. For AChE, the flexible residues were Asp74, Tyr72, Thr75, Trp86, Tyr124, Trp286, Tyr337 and Tyr341.

Blind dockings by setting a grid box to include the entire protein surface were performed. For HDAC1, the grid box was built with a resolution of 1 Å and 50 x 54 x 58 points and it was positioned at the middle of the protein (x = -54.401; y = 11.302; z = -4.214). For HDAC6, a grid box of 44 x 52 x 46 points with grid points separated 1 Å, was positioned at the middle of the protein (x = -5.386; y = 3.841; z = 13.635). For AChE, a grid box of 60 x 60 x 72 points with grid points separated 1 Å, was positioned at the middle of the protein (x = -1.167; y = -37.715; z = 33.661). For BuChE, a grid box of 66 x 60 x 74 points with grid points separated 1 Å, was positioned at the middle of the protein (x = -136.0; y = 123.59; z = 38.56). For 5-HT6, a grid box of 40 x 64 x 40 points with grid points separated 1 Å, was positioned at the middle of the protein (x = 116.569; y = 163.459; z = 140.942).

6. References

(1) Trott O.; Olson, A. J. AutoDock Vina: improving the speed and accuracy of docking with a new scoring function, efficient optimization, and multithreading. *J. Comput. Chem.*, **2010**, 31, 455-461.

(2) Lipinski C. A.; Lombardo F.; Dominy B. W.; Feeney P. J. Experimental and computational approaches to estimate solubility and permeability in drug discovery and development settings. *Adv. Drug. Deliv. Rev.*, **2001**, 46(1-3), 3-26.

(3) Duffy, E. M.; Jorgensen, W. L. Prediction of properties from simulations: Free energies of solvation in hexadecane, octanol, and water. *J. Am. Chem. Soc.*, **2000**, 122, 2878-2888.

(4) Jorgensen, W. L.; Duffy, E. M. Prediction of drug solubility from Monte Carlo simulations. *Bioorg. Med. Chem. Lett.*, **2000**, 10, 1155-1158.

(5) Brooks, B. R.; Brooks III, C. L.; Mackerell, A. D.; Nilsson, L.; Petrella, R. J.; Roux, B.; Won, Y.; Archontis, G.; Bartels, C.; Boresch, S.; Caflisch, A.; Caves, L.; Cui, Q.; Dinner, A. R.; Feig, M.; Fischer, S.; Gao, J.; Hodoscek, M.; Im, W.; Kuczera, K.; Lazaridis, T.; Ma, J.; Ovchinnikov, V.; Paci, E.; Pastor, R. W.; Post, C. B.; Pu, J. Z.; Schaefer, M.; Tidor, B.; Venable, R. M.; Woodcock, H. L.; Wu, X.; Yang, W.; York, D. M.; Karplus, M. CHARMM: The biomolecular simulation program. *J. Comp. Chem.*, **2009**, 30, 1545-1615.
